# Longitudinal Patterns of Symptoms in Patients Undergoing Chemotherapy: A Secondary Analysis of a Cluster Randomized Clinical Trial

**DOI:** 10.1001/jamanetworkopen.2026.4996

**Published:** 2026-04-06

**Authors:** Roshan Paudel, Alexi A. Wright, Christine Cronin, Hajime Uno, Fiona Barrett, Gabriel Brooks, Hannah Hazard Jenkins, Sandra L. Wong, Don S. Dizon, Jessica Bian, Raymond U. Osarogiagbon, Deborah Schrag, Michael J. Hassett

**Affiliations:** 1 Department of Medical Oncology, Dana-Farber Cancer Institute, Boston, Massachusetts; 2 Geisel School of Medicine, Dartmouth-Hitchcock Medical Center, Lebanon, New Hampshire; 3 Department of Surgery, West Virginia University Cancer Center, Morgantown; 4 Emory University School of Medicine, Atlanta, Georgia; 5 Hematology and Oncology, Tufts Medical Center, Boston, Massachusetts; 6 Maine Health Care Center, Portland; 7 Baptist Cancer Center, Baptist Memorial Health Care, Memphis, Tennessee; 8 Department of Medicine, Memorial Sloan Kettering Cancer Center, New York, New York

## Abstract

**Importance:**

The longitudinal patterns of patient-reported outcomes and their association with mortality in routine oncology care are largely unexplored.

**Objectives:**

To characterize the longitudinal patient-reported outcomes among patients undergoing chemotherapy and evaluate their association with mortality.

**Design, Setting, and Participants:**

This secondary analysis assessed patients in the intervention arm of a cluster randomized clinical trial conducted by the Symptom Management Implementation of Patient Reported Outcomes in Oncology (SIMPRO) consortium. Patients with gastrointestinal, gynecologic, or thoracic cancer who started a new chemotherapy regimen and completed at least 1 symptom questionnaire within 180 days at medical oncology clinics at 6 hospitals (Northeast and Southern US) were assessed from September 1, 2019, to August 31, 2023. Data analysis was performed from March to September 2025.

**Exposure:**

Electronic health record–integrated symptom questionnaires along with patient education, decision support alerts, and clinical reports to facilitate symptom management.

**Main Outcomes and Measures:**

Outcomes were patient-reported symptoms, physical function, overall well-being, and mortality. Multivariable regression identified factors associated with mortality during the 180-day follow-up period with symptoms included as time-varying covariates.

**Results:**

Overall, 3735 patients (median [IQR] age, 67 [59-74] years; 2196 [59%] female) submitted 35 059 symptom questionnaires; 1710 (46%) were diagnosed with gastrointestinal, 1163 (31%) with thoracic, and 852 (23%) with gynecologic cancer. On multivariable analysis, moderate (hazard ratio [HR], 2.07; 95% CI, 1.34-3.20; *P* < .001) and severe (HR, 3.39; 95% CI, 2.20-5.22; *P* < .001) physical function deficits were associated with a higher hazard of death. Fatigue was the most common symptom reported (29 138 [83%]) followed by pain (19 959 [57%]). The prevalence of severe symptom reports decreased from 1440 of 30 660 reported symptoms (5%) in week 1 to 40 of 3528 reported symptoms (1%) in week 26, whereas moderate symptom reports decreased from 3522 of 30 660 symptoms (11%) to 265 of 3528 (8%). Other time-varying factors associated with higher hazard of death were moderate pain (HR, 1.43; 95%, CI 1.08-1.90; *P* = .01), severe pain (HR, 1.66; 94% CI, 1.21-2.26; *P* = .001), moderate dyspnea (HR, 1.31; 95% CI, 1.02-1.69; *P* = .04), severe dyspnea (HR, 1.62; 95% CI, 1.17-2.24; *P* = .004), moderate loss in appetite (HR, 1.40; 95% CI, 1.02-1.92; *P* = .04), and severe loss in appetite (HR, 2.27; 95% CI, 1.55-3.33; *P* < .001).

**Conclusions and Relevance:**

This secondary analysis of a cluster randomized clinical trial of patients with cancer characterized the burden of cancer-related symptoms and described normative experiences for patients receiving chemotherapy for 3 common types of cancer and found that mortality was associated with patients’ evolving health status. These findings may help inform programs to monitor and manage symptoms and to deliver targeted interventions that enhance outcomes.

**Trial Registration:**

ClinicalTrials.gov Identifier: NCT03850912

## Key Points

**Question:**

How do patient-reported symptoms manifest during a 180-day period, and are longitudinal symptoms associated with mortality in patients undergoing chemotherapy for a gastrointestinal, gynecologic, or thoracic cancer in routine oncology practice settings?

**Findings:**

In this secondary analysis of a cluster randomized clinical trial of patient-reported outcomes that included 3725 patients undergoing chemotherapy, pain, fatigue, and deficits in physical function and overall well-being were the most frequently reported symptoms. Moderate to severe deficits in physical function as well as moderate to severe pain, dyspnea, and decreased appetite were significantly associated with a higher hazard of mortality 180 days after starting chemotherapy.

**Meaning:**

These results suggest that recognizing which symptoms are associated with an increased hazard of death may help to better support patients with high symptom burden and increased risk of mortality.

## Teaser

This secondary analysis of a cluster randomized clinical trial examines the longitudinal patterns of symptoms reported by patients receiving chemotherapy for thoracic, gastrointestinal, and gynecologic cancers and assesses the association between longitudinal symptom reports and death.

## Introduction

Patients with cancer report a wide range of disease- and treatment-related symptoms. Prior studies have documented discrepancies between clinician- and patient-reported symptoms, with clinicians recording fewer symptoms and lower symptom severity than patients.^1,2^ Remote monitoring of symptoms improves quality of life, reduces symptom burden, decreases emergency department visits and hospitalizations, and even prolongs survival outcomes.^3,4,5,6^ Several large-scale implementation studies^7,8,9,10^ have demonstrated the utility of electronic patient-reported outcomes (ePROs) in routine practice. As a result, an increasing number of oncology practices seek to use ePROs to remotely monitor symptoms, physical function (PF), overall well-being (OWB), and other adverse events.^11,12^

The Symptom Management of Patient-Reported Outcomes in Oncology (SIMPRO) Research Center deployed electronic symptom management (eSyM), an electronic health record–integrated PRO system, at 6 US cancer centers to assess the effectiveness of ePRO monitoring for reducing unplanned acute care.^13^ Through a pragmatic, cluster randomized, stepped-wedge trial, eSyM was implemented at the Baptist Cancer Center, Dana-Farber Cancer Institute, Dartmouth-Hitchcock Medical Center, Brown University Health, Maine Medical Center, and West Virginia University Cancer Center, serving demographically diverse populations living in geographically dispersed communities. eSyM systematically prompts patients to respond to questions about a group of 12 common symptoms using the PRO-Common Terminology Criteria for Adverse Events items as well as respond to one question on PF and one on OWB via the patient portal.

With the exception of a few studies,^14,15^ routinely collected ePRO data have not been used extensively to characterize patients’ longitudinal symptom trajectories.^16,17^ Furthermore, the association between these longitudinal patient symptom patterns and overall survival is not well characterized because existing evidence demonstrating the prognostic value of PROs largely comes from baseline data.^18^ We examined the longitudinal patterns of symptoms reported by patients receiving chemotherapy for thoracic, gastrointestinal, and gynecologic cancers. We also assessed the association between longitudinal symptom reports and the hazard of death.

## Methods

### Data

We performed a secondary analysis of PRO data collected prospectively from individuals with gastrointestinal, thoracic, or gynecologic cancer initiating chemotherapy at one of the SIMPRO research sites. Patients with new chemotherapy plans were automatically assigned eSyM questionnaires after treatment initiation through their institution-specific patient portal. Patients’ symptom reports were solicited twice per week for 6 months or until chemotherapy treatment was discontinued, whichever came first.^13^ We included patients treated at a participating SIMPRO site after eSyM deployment who completed at least one symptom questionnaire. Patients were able to complete a symptom questionnaire through their patient portals using mobile devices or personal computers. Because eSyM was implemented using a stepped-wedge design, participating health systems spent different amounts of time in the control and intervention phases. We collected symptom data from patients between September 1, 2019, and August 31, 2023. Race was a required National Cancer Institute data element for the common data model, so we collected this information via the EHR as we did with other patient demographic information. Race categories included Asian, Black, White, and other (Native American, Native Hawaiian or Other Pacific Islander, other race, multiple races, or refusal to report) or unknown race. The study design and outcomes of the parent cluster randomized clinical trial are described in the trial protocol (Supplement 1).^27^ The parent trial was approved by the Western institutional review board, which waived informed consent because the intervention was considered to be part of routine clinical care. We report a planned secondary analysis of symptom data from the parent cluster randomized clinical trial, following the Consolidated Standards of Reporting Trials (CONSORT) guidelines for reporting results.

### Outcome and Measures

The eSyM questionnaire required patients to report on 12 symptoms, including anxiety, constipation, decreased appetite, diarrhea, dyspnea, fatigue, nausea, neuropathy, pain, rash, trouble drinking fluid, and vomiting, as well as PF and OWB. Although these symptoms were presented as required, patients could submit incomplete questionnaires, so submissions may not have included scores for all required symptoms. Participants had the option of reporting up to 18 additional symptoms. Free-text reports were not allowed, but patients could message their care teams via the patient portal. Symptom items were derived from a modified version of the PRO–Common Terminology Criteria for Adverse Events,^19^ a validated PRO measure, where the recall period was shortened from 7 days to 24 hours to focus on symptoms requiring active management vs those that had already resolved (this change had a minimal impact on the frequency of severe symptom reporting).^20^ For each symptom, patients reported occurrence, frequency, and interference, which were used to derive a composite numerical score of 0 (none), 1 (mild), 2 (moderate), or 3 (severe) based on prior work.^13,21^ Each questionnaire with at least 1 symptom score of 3 generated a clinical alert that prompted patients and clinicians to connect. PF and OWB were rated on 0- to 3-point scale, with 0 indicating no deficit and 3 indicating severe deficit. Distributions of symptom scores, PF, and OWB were assessed by week after initiation of the chemotherapy treatment plan. If a patient submitted 2 questionnaires in 1 week, both questionnaires were independently included in the analysis. A questionnaire response was considered complete if all 12 symptoms were reported; patients with incomplete questionnaires were excluded.

### Statistical Analysis

Descriptive statistics were used to compare demographic and clinical characteristics stratified by cancer type. We calculated the total number of patients completing questionnaires for each week during the 6-month period. We assessed the numbers and percentages by symptom severity. We further assessed the total number of symptom alerts generated per week. To characterize trends, we plotted the proportion of patients with symptoms scored 0 to 3 stratified by cancer type to visualize fluctuations during a 26-week period. Symptom scores 2 and 3 were collapsed into a moderate to severe category because scores of 3 were uncommon. Similarly, we plotted co-occurrences of the 6 most common moderate to severe symptoms to illustrate the patterns and extent of symptom co-occurrences.^22,23^

We fit a Cox proportional hazards regression model to estimate the hazard of death during the 180-day period from the questionnaire assignment date. Patients were administratively censored at 180 days after the questionnaire assignment date. The end of follow-up was defined as the date of death or the censoring date, whichever came first. For each patient, the follow-up time was calculated from the date of questionnaire assignment to the date of death or censoring. The model included baseline clinical, sociodemographic, and time-varying PRO data as covariates. The model was fit using the data in counting-process start and stop format with time-varying covariates as proposed by Therneau et al^24^ (eFigure 1 in Supplement 2). Multiple records per patient reflected time-split intervals, and robust variance estimates were used to obtain valid SEs.^25^ Hazard ratios (HRs) and 95% CIs were estimated. Significance was set at 2-tailed *P* < .05. Data analysis was performed from March to September 2025. All analyses were conducted using R, version 4.2.2 (R Foundation for Statistical Computing) and software packages, including survival^25^ and gtsummary.^26^

## Results

### Patient Characteristics

**Figure 1.**
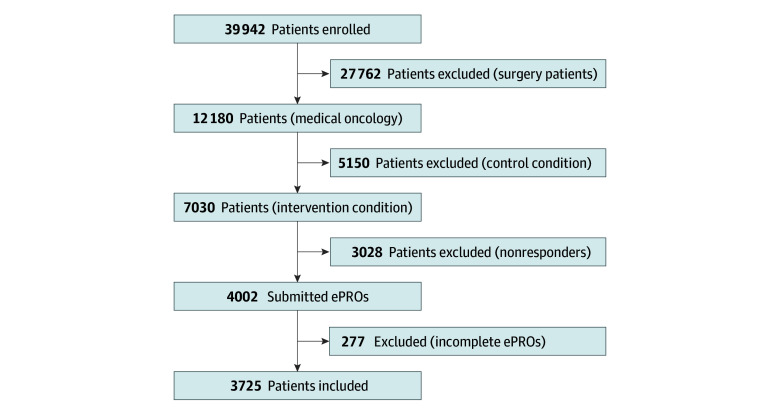
CONSORT Flow Diagram of the Study Participants ePRO indicates electronic patient-reported outcome.

We identified 3725 patients (median [IQR] age, 67 [59-74] years; 2196 [59%] female and 1528 [41%] male; 51 [1%] Asian, 353 [10%] Black, 3233 [87%] White, and 88 [2%] other or unknown race) who submitted 35 059 symptom questionnaires during the study period. A total of 277 patients with incomplete questionnaires were excluded from the analysis (Figure 1 and eFigure 5 in Supplement 2). Gastrointestinal cancer was the most common diagnosis with 1710 patients (46%), followed by 1163 (31%) with thoracic cancer and 852 (23%) with gynecologic cancers. A total of 1130 patients (30%) were receiving treatment with curative intent, 1076 (29%) with palliative intent, and 1519 (41%) had no treatment intent specified (Table 1). The median (IQR) number of questionnaires submitted per patient was 4 (1-12) (range, 1-124).

**Table 1.** Characteristics of Patients Undergoing Systemic Therapies Stratified by Cancer Type

Characteristic	No. (%) of patients^a^			
	Gastrointestinal cancer (n = 1710)	Gynecologic cancer (n = 852)	Thoracic cancer (n = 1163)	Overall (N = 3725)
Age, median (IQR), y	66 (57-73)	65 (57-72)	69 (62-75)	67 (59-74)
Sex				
Female	739 (43)	851 (100)	606 (52)	2196 (59)
Male	971 (57)	0	557 (48)	1528 (41)
Race				
Asian	20 (1)	16 (2)	15 (1)	51 (1)
Black	165 (10)	99 (12)	89 (8)	353 (10)
White	1482 (87)	714 (84)	1037 (89)	3233 (87)
Other or unknown^b^	43 (3)	23 (3)	22 (2)	88 (2)
Ethnicity				
Hispanic	34 (2)	10 (1)	12 (1)	56 (2)
Not Hispanic	1618 (95)	821 (96)	1095 (94)	3534 (95)
Unknown	58 (3)	21 (3)	56 (5)	135 (4)
Marital status				
Divorced	136 (8)	92 (11)	130 (11)	358 (10)
Married or civil union	1144 (67)	462 (54)	682 (59)	2288 (61)
Single	238 (14)	164 (19)	144 (12)	546 (15)
Widowed	141 (8)	106 (12)	169 (15)	416 (11)
Other or unknown	51 (3)	28 (3)	38 (3)	117 (3)
Insurance				
Dual eligible	49 (3)	26 (3)	59 (5)	134 (4)
Medicaid	81 (5)	50 (6)	43 (4)	174 (5)
Medicare	651 (38)	336 (39)	553 (48)	1540 (41)
Private	883 (52)	395 (46)	474 (41)	1752 (47)
Self-pay or uninsured	46 (3)	45 (5)	34 (3)	125 (3)
Employment				
Disabled	171 (10)	75 (9)	131 (11)	377 (10)
Full time	407 (24)	196 (23)	161 (14)	764 (21)
Not employed	150 (9)	90 (11)	91 (8)	331 (9)
Part time	42 (3)	31 (4)	28 (2)	101 (3)
Retired	823 (48)	393 (46)	658 (57)	1874 (50)
Other or unknown	117 (7)	67 (8)	94 (8)	278 (8)
Language				
English	1684 (98)	841 (99)	1153 (99)	3678 (99)
Other or unknown	26 (2)	11 (1)	10 (1)	47 (1)
Treatment goal				
Curative	484 (28)	310 (36)	336 (29)	1130 (30)
Palliative	464 (27)	151 (18)	461 (40)	1076 (29)
Not specified	762 (45)	391 (46)	366 (31)	1519 (41)
SIMPRO site				
Baptist Health Cancer Center	592 (35)	275 (32)	491 (42)	1358 (36)
Dana-Farber Cancer Institute	478 (28)	198 (23)	308 (26)	984 (26)
Dartmouth-Hitchcock Medical Center	129 (8)	90 (11)	105 (9)	324 (9)
Lifespan Cancer Institute	238 (14)	47 (6)	111 (10)	396 (11)
Maine Medical Center	167 (10)	217 (25)	105 (9)	489 (13)
West Virginia University	106 (6)	25 (3)	43 (4)	174 (5)

Abbreviation: SIMPRO, Symptom Management Implementation of Patient Reported Outcomes in Oncology.

^a^ Unless otherwise indicated.

^b^ Other race includes Native American, Native Hawaiian or Other Pacific Islander, other race, multiple races, or refusal to report.

### Symptom Prevalence

**Figure 2.**
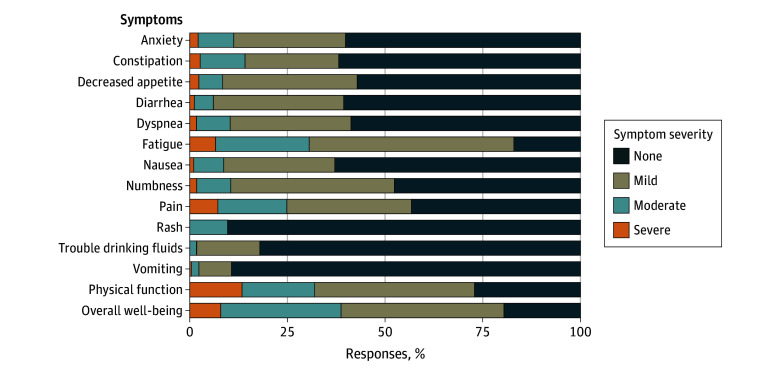
Bar Graph of the Distribution of the 12 Most Common Symptoms, Physical Function, and Overall Well-Being^a^ ^a^Data from the study’s 35 059 questionnaires.

Overall, fatigue and pain were the most frequently reported symptoms. When aggregated across all time points, 29 138 of 35 059 questionnaires (83%) included a score for fatigue; among these 35 059 questionnaires, 2322 (7%) reported severe fatigue, 8394 (24%) moderate fatigue, and 18 422 (53%) mild fatigue. Pain was reported in 19 959 of the 35 059 questionnaires of which 2534 (7%) included severe, 6172 (18%) moderate, and 11 253 (32%) mild pain. Nausea, dyspnea, and decreased appetite were somewhat prevalent, whereas vomiting was the least prevalent symptom (3665 [10%]) across all time points (Figure 2). We did not observe marked differences in symptoms by cancer type, except for dyspnea in patients with thoracic and dysuria in patients with gynecologic cancers (eFigures 6 and 7 in Supplement 2). Of the symptoms that were not prompted in the core eSyM questionnaire but for which participants could elect to respond, insomnia (6657 [19%]) and cough (5201 [15%]) were most frequently reported across all time points (eFigure 2 in Supplement 2).

### Questionnaire Response and Symptom Prevalence Over Time

There was a gradual decrease in the number of patients who submitted questionnaires over time. In week 1, 1886 patients submitted 2555 questionnaires, in week 13, 961 patients submitted 1358 questionnaires, and in week 26, 275 patients submitted 294 questionnaires (eTable in Supplement 2). The prevalence of severe symptom reports decreased from 1440 of 30 660 reported symptoms (5%) in week 1 to 40 of 3528 reported symptoms (1%) in week 26, whereas moderate symptom reports decreased from 3522 of 30 660 symptoms (11%) to 265 of 3528 (8%). In contrast, the frequency of mild symptom reports remained stable over time (8331 of 30 660 [27%] in week 1 and 903 of 3528 [26%] in week 26), and the frequency of no symptoms (score 0) increased over time (eTable in Supplement 2).

### Symptom Alerts Over Time

A symptom alert was generated whenever a questionnaire included at least 1 severe symptom report. In total, 4979 symptom alerts were generated during the 26-week period (4979 questionnaires [14%] and 1921 patients [51%]). The number of symptom alerts was highest during week 1 when 717 of 2555 questionnaires (28%) generated an alert. The prevalence of symptom alerts gradually decreased over time to 194 of 1314 completed questionnaires (15%) by week 8 and 24 of 294 completed questionnaires (8%) by week 26 (eTable in Supplement 2).

### Symptom Patterns

**Figure 3.**
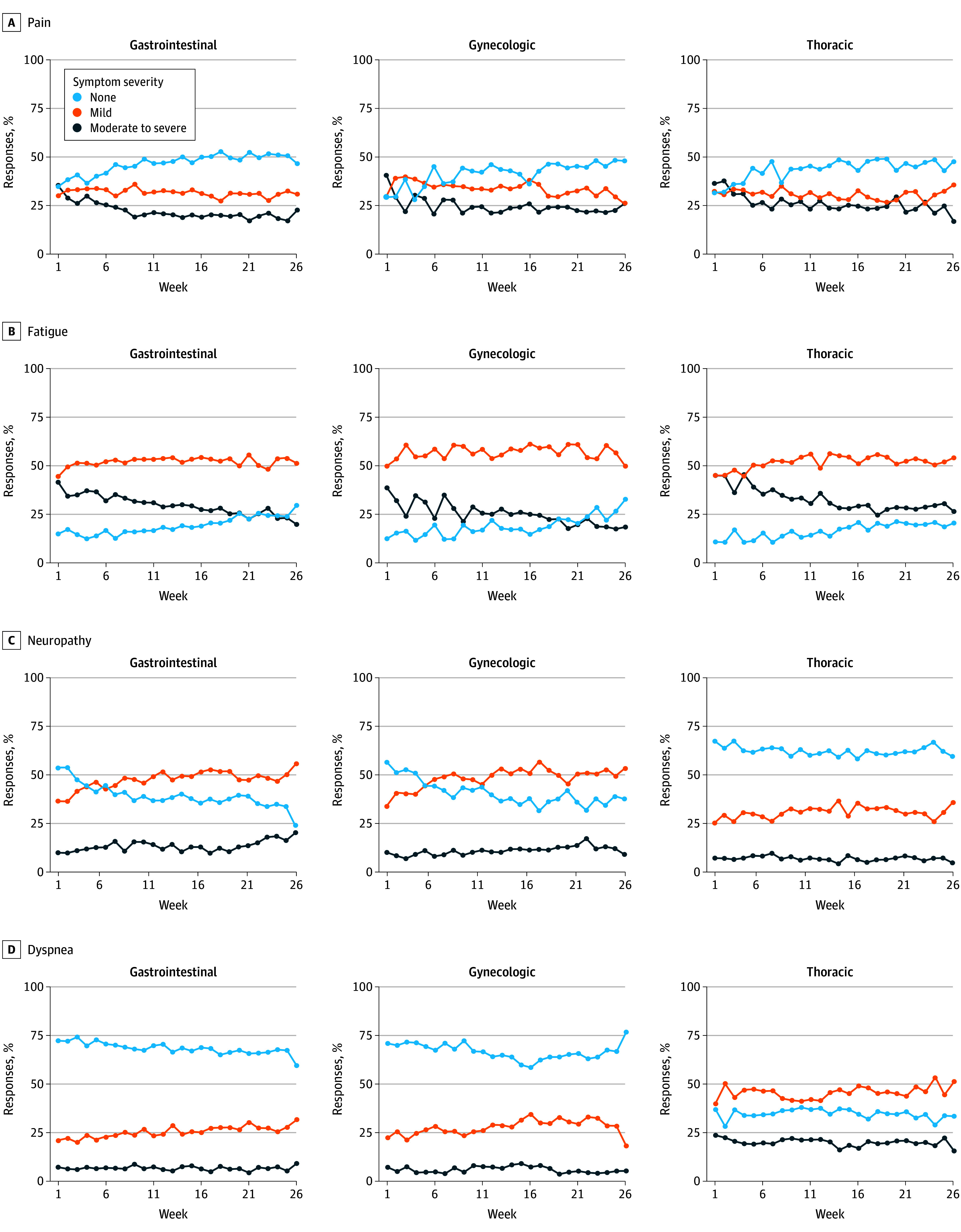
Line Graphs of Symptom Scores Stratified by Cancer Type for 4 Common Symptoms

We observed a gradual decrease in the proportion of moderate to severe symptoms reported over time across all 3 cancer types, but mild symptoms persisted. Moderate to severe anxiety, constipation, decreased appetite, nausea, pain, and vomiting improved over time, but we did not observe an improvement in fatigue, neuropathy, or dyspnea. Most questionnaires included reports of mild fatigue and neuropathy, with increasing numbers of patients reporting mild neuropathy over time. Reports of dyspnea were mild and largely stable among patients with gastrointestinal and gynecologic cancers, but patients with thoracic cancers reported more moderate or severe dyspnea consistently over time (Figure 3; eFigure 3 in Supplement 2).

### Co-Occurrences of Moderate to Severe Symptoms

Among the 6 most common symptoms, moderate to severe fatigue alone was reported in 2475 (15%) questionnaires, followed by pain (1996 [12%]) and constipation (1036 [6%]). Less than half (7055 [42%]) of questionnaires included a single moderate to severe symptom, with progressively fewer co-occurrences: 4723 (28%) including 2 symptoms and 2834 (17%) including 3 symptoms. The most prevalent moderate to severe symptom pair was fatigue-pain (1263 [8%]) followed by fatigue-dyspnea (697 [4%]) and fatigue-neuropathy (515 [3%]). The most common symptom triads included fatigue-pain-constipation (501 [3%]) followed by fatigue-pain-anxiety (469 [3%]) and fatigue-pain-dyspnea (442 [2.6%]). As the number of symptoms in a combination increased, the number of questionnaires reporting such combinations decreased (eFigure 4 in Supplement 2).

### Association of Longitudinal Patient-Reported Data and 180-Day Mortality

We fit a Cox proportional hazards regression model to assess the hazards of death within the 180 days after treatment initiation, treating patient-reported symptoms, PF, and OWB as time-varying and patients’ baseline characteristics as fixed covariates. We observed 551 all-cause deaths (15%) in the 180-day period. On multivariable analysis (Table 2), moderate PF deficit (HR, 2.07; 95% CI, 1.34-3.20; *P* < .001) and severe PF deficits (HR, 3.39; 95% CI, 2.2-5.22; *P* < .001) were associated with higher hazard of death. Other time-varying factors associated with higher hazard of death were moderate pain (HR, 1.43; 95%, CI 1.08-1.90; *P* = .01), severe pain (HR, 1.66; 94% CI, 1.21-2.26; *P* = .002), moderate dyspnea (HR, 1.31; 95% CI, 1.02-1.69; *P* = .03), severe dyspnea (HR, 1.62; 95% CI, 1.17-2.24; *P* = .004), moderate loss in appetite (HR, 1.40; 95% CI, 1.02-1.92; *P* = .04), and severe loss in appetite (HR, 2.27; 95% CI, 1.55-3.33; *P* < .001). Vomiting, regardless of severity, was also a significant risk factor, with HRs ranging from 1.72 (95% CI, 1.35-2.20) for mild to 3.41 (95% CI, 1.71-6.81) for severe vomiting. The presence of rash and neuropathy were associated with lower hazard of death. Baseline characteristics, including palliative treatment goal (HR, 3.20; 95% CI, 2.34-4.37; *P* < .001), increasing age (HR, 1.02; 95% CI, 1.00-1.03; *P* = .005), and Charlson Comorbidity Index of 1 (HR, 1.25; 95% CI, 1.01-1.56; *P* = .04) were associated with higher and female sex (HR, 0.64; 95% CI, 0.55-0.84; *P* < .001) with lower hazard of death (Table 2).

**Table 2.** Factors Associated With 180-Day Mortality in Multivariable Analysis

Characteristic	HR (95% CI)	*P* value
Time-varying factors		
Physical function (reference, no deficit)		
Mild deficit	1.24 (0.83-1.84)	.30
Moderate deficit	2.07 (1.34-3.20)	<.001
Severe deficit	3.39 (2.20-5.22)	<.001
Overall well-being (reference, no deficit)		
Mild deficit	1.46 (0.88-2.41)	.14
Moderate deficit	1.49 (0.87-2.54)	.14
Severe deficit	1.51 (0.85-2.67)	.16
Pain (reference, none)		
Mild	1.17 (0.89-1.53)	.28
Moderate	1.43 (1.08-1.90)	.01
Severe	1.66 (1.21-2.26)	.002
Fatigue (reference, none)		
Mild	0.98 (0.62-1.54)	.94
Moderate	1.22 (0.75-1.99)	.41
Severe	1.35 (0.79-2.29)	.27
Nausea (reference, none)		
Mild	1.03 (0.82-1.30)	.81
Moderate	0.88 (0.64-1.20)	.41
Severe	0.62 (0.37-1.06)	.08
Dyspnea (reference, none)		
Mild	1.27 (1.03-1.58)	.02
Moderate	1.31 (1.02-1.69)	.03
Severe	1.62 (1.17-2.24)	.004
Anxiety (reference, none)		
Mild	0.86 (0.68-1.07)	.18
Moderate	0.99 (0.76-1.29)	.94
Severe	1.03 (0.70-1.51)	.86
Diarrhea (reference, none)		
Mild	0.91 (0.75-1.10)	.33
Moderate	1.09 (0.79-1.51)	.60
Severe	0.89 (0.52-1.54)	.68
Constipation (reference, none)		
Mild	1.01 (0.81-1.26)	.90
Moderate	0.85 (0.65-1.10)	.23
Severe	1.0 (0.70-1.41)	.99
Decreased appetite (reference, none)		
Mild	1.24 (0.98-1.57)	.08
Moderate	1.40 (1.02-1.92)	.04
Severe	2.27 (1.55-3.33)	<.001
Vomiting (reference, none)		
Mild	1.72 (1.35-2.20)	<.001
Moderate	1.91 (1.30-2.80)	<.001
Severe	3.41 (1.71-6.81)	<.001
Trouble drinking fluids (reference, none)		
Mild	1.00 (0.80-1.25)	>.99
Moderate	1.38 (0.95-2.00)	.09
Rash (present)^a^	0.74 (0.55-0.99)	.04
Neuropathy (reference, none)		
Mild	0.75 (0.62-0.92)	.006
Moderate	0.64 (0.49-0.84)	.001
Severe	0.59 (0.36-0.97)	.04
Time-invariant factors		
Age	1.02 (1.01-1.03)	.005
Female sex	0.64 (0.53-0.78)	<.001
Race (reference, White)		
Asian	0.75 (0.33-1.73)	.50
Black	1.06 (0.71-1.58)	.78
Other or unknown	1.31 (0.68-2.54)	.35
Ethnicity		
Hispanic	0.83 (0.34-0.25)	.68
Non-Hispanic	0.90 (0.62-1.33)	.61
Marital status (reference, married or civil union)		
Single	1.01 (0.77-1.33)	.94
Divorced	0.79 (0.53-1.16)	.22
Widowed	1.04 (0.78-1.40)	.76
Other or unknown	0.63 (0.34-1.17)	.15
Employment status (reference, full time)		
Retired	0.75 (0.55-1.02)	.06
Part time	0.63 (0.30-1.34)	.23
Disabled	0.56 (0.37-0.85)	.006
Not employed	0.99 (0.67-1.47)	.96
Other or unknown	0.77 (0.53-1.12)	.21
Charlson Comorbidity Index (reference, 0)		
1	1.26 (1.01-1.56)	.04
2	1.01 (0.73-1.41)	.95
Treatment goal (reference, curative)		
Not specified	2.28 (1.67-3.12)	<.001
Palliative	3.20 (2.34-4.37)	<.001
Insurance (reference, Medicaid)		
Medicare	0.98 (0.57-1.68)	.95
Dual eligible	0.88 (0.45-1.71)	.72
Private	0.92 (0.55-1.53)	.74
Self-pay or uninsured	0.87 (0.41-1.85)	.71

Abbreviation: HR, hazard ratio.

^a^ Rash is binary (present or absent).

## Discussion

We report a comprehensive assessment of the frequency, severity, and co-occurrence of symptoms experienced by 3725 patients from 6 geographically diverse health systems who were receiving chemotherapy for gastrointestinal, thoracic, or gynecologic cancer. The prevalence of severe symptom reports decreased from 5% in week 1 to 1% in week 26, whereas moderate symptom reports decreased from 11% to 8%. As time passed after the start of chemotherapy, the frequency of moderate or severe symptoms decreased; relatively few severe symptoms were reported after the first 2 weeks of treatment. This finding could be from improvements in symptom control due to optimization of symptom management, modifications to chemotherapy regimens, or tumor responsiveness to chemotherapy. The persistence of mild symptoms, which were reported with a steady frequency over time, suggests that many patients experience symptoms that could impact their quality of life throughout their course of treatment. The eSyM intervention generated alerts for severe symptoms but not for mild to moderate symptoms. Early interventions for severe symptoms may have helped to prevent future occurrences of severe symptoms, but interventions targeting mild to moderate symptoms may be needed to address the symptom burden that persists throughout chemotherapy treatment.

Fatigue was the most frequently reported symptom, followed by pain, constipation, anxiety, neuropathy, nausea, and dyspnea. Although the frequency with which patients reported severe symptoms decreased over time for most symptoms, peripheral neuropathy increased as treatment progressed among patients treated for gynecologic and gastrointestinal cancers, potentially indicating ongoing treatment-related adverse events. As noted, fatigue was reported more frequently than any other symptom across all time points. Furthermore, mild fatigue remained common during the 26-week period, with most patients reporting mild fatigue. Moderate to severe fatigue was the most common symptom to co-occur with other symptoms, signifying its potential sentinel role as part of symptom clusters. The frequency of fatigue across all time points, its persistence during the 26-week period, and its co-occurrence with other symptoms reinforce the difficulty of effectively managing fatigue. Fatigue has been recognized as a major quality-of-life issue in studies of patients and practitioners^28^ and in patients undergoing systemic therapy for solid tumors and blood cancers.^29^ These findings highlight the continued need to develop and implement more interventions to help reduce fatigue and support patients receiving systemic treatment.

Our findings are consistent with prior studies that have reported on the temporality of pain, fatigue, nausea, and both PF and OWB in patients diagnosed with breast,^30^ lung,^31^ and ovarian cancers.^32^ For example, an analysis of patients with lung cancer in a cohort within the PRO-TECT trial^31^ found that pain was the most frequent and persistent symptom impacting patients’ activity level. A longitudinal study of patients with early-stage breast cancer similarly found considerable variability in the experience of fatigue.^30^ In this study, approximately 78% of women with elevated fatigue at baseline did not recover within 18 months after the completion of radiation therapy and/or chemotherapy.^30^ Lastly, a prospective cohort study of women with ovarian cancer found that more than half (57%) reported clinically significant fatigue after completion of primary treatment.^32^

Additionally, our results demonstrate that some, but not all, PROs are independently associated with increased hazard of death. Increasing impairment in PF and increasing severity in pain, dyspnea, decreased appetite, and vomiting were associated with increased hazard of death. We observed a progressive increase in the hazard of death for each step increase in these PROs, underscoring their importance as risk factors associated with mortality. Other studies have established the prognostic value of PROs in patients with cancer.^33,34,35^ In fact, a recent systematic review and meta-analysis found that higher baseline scores on global health status and quality of life as well as functional domains were associated with improved survival, whereas greater symptom burden was associated with inferior survival.^18^ Using longitudinal data collected in routine care, we fit time-varying Cox proportional hazards regression models to estimate mortality within 180 days using symptom inputs that reflected patients’ evolving health status throughout the course of treatment. This approach may be more robust and useful than historical approaches that relied on pretreatment data to estimate posttreatment outcomes.^36^

### Limitations

This study has several limitations that merit attention. First, eSyM was developed to provide active symptom management after treatment initiation; therefore, pretreatment symptom data were not collected. The absence of pretreatment data limits our ability to assess the extent to which baseline symptoms were associated with posttreatment symptom burden or whether symptoms recovered to baseline levels over time. Nevertheless, the longitudinal data show typical time windows for symptom improvement and peak symptom burden across 3 types of cancers. Second, symptom reporting was voluntary, with just half of all eligible patients reporting symptoms in this pragmatic trial.^37^ The symptom experiences of nonresponders and responders may differ. Third, we were unable to ascertain the effects of different chemotherapy regimens on symptom manifestation. Future studies will explore differences in symptom profiles across different chemotherapy treatment plans. Fourth, we could not account for extent of disease as a potential risk factor. Cancer staging information was frequently missing because it was not routinely captured across tumor types. Therefore, we included chemotherapy intent, which is a partial surrogate for localized or metastatic disease and found that it was associated with increased symptom burden as expected. Fifth, there was patient attrition after the start of chemotherapy, with fewer patients reporting symptoms over time. Other studies^10,38,39^ have similarly documented a gradual reduction in questionnaire completion rates over time. We cannot determine whether the attrition was related to decreasing symptom burden or other factors. Sixth, our data were limited to patients with gastrointestinal, gynecologic, and thoracic cancers, so the results may not generalize to other cancers.

## Conclusions

In this secondary analysis of a cluster trial of patients undergoing chemotherapy, we found that longitudinal PRO data are independently associated with an increased risk of death within 180 days. These results help set expectations for symptom recovery and identify patients whose symptoms deviate from expected patterns.

## Notes

Supplement 1.
Trial Protocol and Statistical Analysis Plan

Supplement 2. **eFigure 1.** An Illustration of Start and Stop Counting Process for Time-Varying Cox Proportional Hazards Model **eTable.** Frequencies and Percentages of Symptom Alerts and Composite Scores by Week **eFigure 2.** Distribution of 18 Optional Symptoms by Scores** **eFigure 3.** Symptom Scores Stratified by Cancer Type **eFigure 4.** Unique Co-Occurrences of Moderate to Severe Symptoms Across All Time Points **eFigure 5.** CONSORT SIMPRO **eFigure 6.** Distribution of 12 Required Symptoms, Physical Function and Overall Wellbeing by Severity for Patients with GYN Cancer (N=9474 Surveys) **eFigure 7.** Distribution of 12 Required Symptoms by Severity for Patients with Thoracic Cancer (N=9967 Surveys)

Supplement 3.
Data Sharing Statement
