# Supplementary material for: Longitudinal Patterns of Symptoms in Patients Undergoing Chemotherapy: A Secondary Analysis of a Cluster Randomized Clinical Trial
Source: JAMA Netw Open. 2026 Apr 6;9(4):e264996. doi: 10.1001/jamanetworkopen.2026.4996 (PMC13054620; doi:10.1001/jamanetworkopen.2026.4996)
Supplement: Supplement 1. — Trial Protocol and Statistical Analysis Plan [file jamanetwopen-e264996-s001.pdf]

PROTOCOL TITLE:

SIMPRO Research Center: Integration and Implementation of PROs for Symptom Management in Oncology Practice.

Sponsor and Coordinating Center:

Dana-Farber/Harvard Cancer Center (DF/HCC)
Department of Medical Oncology
Coordinating Center PI and Technology PI: Michael Hassett
Dana-Farber Cancer Institute
Boston, MA 02215
Email Address: Michael_Hassett@dfci.harvard.edu
Telephone Number: 617-632-4587

CO-STUDY CHAIRS:

Deborah Schrag MD, MPH
Chair, Department of Medicine
Memorial Sloan Kettering Cancer Center
New York City, NY 10065

Raymond Osarogiagbon MD
Director, Thoracic Oncology Research Group
Baptist Memorial Hospital
Covington, Tennessee 38019

Sandra Wong MD
Chair, Department of Surgery
Dartmouth College
Lebanon, NH 03756

VERSION NUMBER:

Version 9

DATE:

Version date: August 4, 2023

FUNDING:

National Cancer Institute, UM1CA233080.

ACRONYMS USED THROUGHOUT PROTOCOL:

eSyM	Electronic symptom management system
ePRO	Electronic patient-reported outcomes
EHR	Electronic health record
eSyM+	Assigned to use eSyM
eSyM-	Assigned not to use eSyM
Gyn	Gynecologic
GI	Gastrointestinal
SOP	Standard operating procedure
FTP	File transfer protocol
SASS	Research questionnaire
SIV	Site initiation visit
CTMS	Clinical trial management system
DF/HCC	Dana-Farber/Harvard Cancer Center
ODQ	DF/HCC Office of Data Quality
QI	Quality Improvement
NHSR	Not Human Subjects Research

Table of Contents

Contents

1.0 Schema 4
2.0 Background 5
3.0 Objectives 8
3.1 Hypothesis 8
4.0 Inclusion and Exclusion Criteria or Activity Population 9
5.0 Protocol Activities 9
6.0 Study-Wide Number of Subjects 31
7.0 Study-Wide Recruitment Methods 32
8.0 Multi-Site Research 32
9.0 Study Timelines 32
10.0 Study Endpoints and Statistical Analyses 32
11.0 Procedures Involved 36
12.0 Data Management and Confidentiality 36
13.0 Provisions to Monitor the Data to Ensure the Safety of Subjects 46
14.0 Future Use of Data 46
15.0 Withdrawal of Subjects 47
16.0 Risks to Subjects 47
17.0 Potential Benefits to Subjects 48
18.0 Vulnerable Populations 48
19.0 Sharing of Results with Subjects 48
20.0 Setting 48
21.0 Resources Available 48
22.0 Provisions to Protect the Privacy Interests of Subjects 49
23.0 Compensation for Research-Related Injury 49
24.0 Economic Burden to Subjects 49
25.0 Consent Process 49
26.0 Appendices 50

1.0 Schema

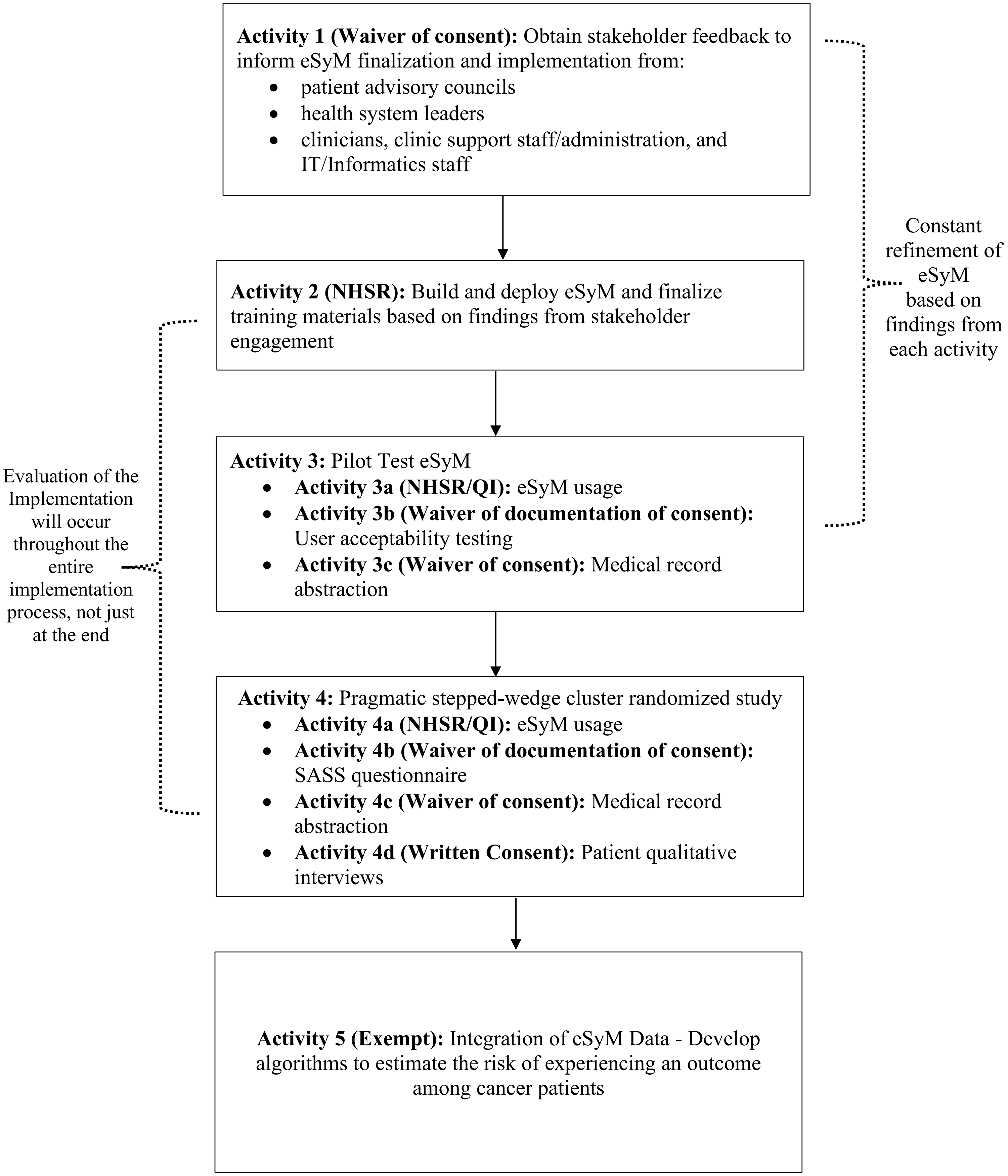
Activity 1 (Waiver of consent): Obtain stakeholder feedback to inform eSyM finalization and implementation from:
patient advisory councils
health system leaders
clinicians, clinic support staff/administration, and IT/Informatics staff
Constant refinement of eSyM based on findings from each activity
Activity 2 (NHSR): Build and deploy eSyM and finalize training materials based on findings from stakeholder engagement
Evaluation of the Implementation will occur throughout the entire implementation process, not just at the end
Activity 3: Pilot Test eSyM
Activity 3a (NHSR/QI): eSyM usage
Activity 3b (Waiver of documentation of consent): User acceptability testing
Activity 3c (Waiver of consent): Medical record abstraction
Activity 4: Pragmatic stepped-wedge cluster randomized study
Activity 4a (NHSR/QI): eSyM usage
Activity 4b (Waiver of documentation of consent): SASS questionnaire
Activity 4c (Waiver of consent): Medical record abstraction
Activity 4d (Written Consent): Patient qualitative interviews
Activity 5 (Exempt): Integration of eSyM Data - Develop algorithms to estimate the risk of experiencing an outcome among cancer patients

2.0 Background

Deficits in management of common symptoms cause substantial morbidity for cancer patients. In the United States, nearly 1.74 million people will be diagnosed with cancer in 2018.[1] While there will be approximately 600,000 cancer deaths, better treatment has contributed to lower mortality rates. However, the morbidity toll of cancer treatment remains immense.[1-5] Poor symptom control decreases quality of life, increases the need for emergency care,[6-8] and even deters some patients from receiving effective therapy.[9,10] For patients with cancer receiving chemotherapy, adverse symptoms add to distress. For surgical patients, poorly managed symptoms delay recovery and interfere with timely receipt of adjuvant treatments[11-13] and the return to usual activity levels. Patients are reluctant to “complain” or perceive that symptoms are unavoidable.[14] The raging US opioid epidemic has further complicated pain management for cancer patients.[15]

Because the health care delivery system is structured to be reactive and not proactive, there are missed opportunities to optimize symptom control. The current cancer care delivery system is not well-equipped to anticipate, monitor and prevent adverse symptoms before they escalate.[16] Typically, patients initiate outreach to address a problem which clinicians try to solve during office visits. Between these face-to-face encounters, communication is scarce and almost entirely patient initiated. For patients with chronic illness like cancer, this model is maladaptive. Many choose to endure their symptoms, hesitate to adjust medications, or are reluctant to mention adverse symptoms for fear of compromising the ability to receive treatment. Moreover, effective symptom control typically requires careful titration of combinations of pain, nausea, and bowel medications to achieve optimal equilibrium. Although many patients and their caregivers gain proficiency over time, others struggle to cope, particularly at treatment initiation or care transitions. Surgeons, medical oncologists, and oncology nurses have experience with symptom management, but they are often preoccupied by treatment decisions about cancer therapy during visits.[17] In some settings, palliative care physicians, rehabilitation specialists, and social workers partner with oncologists to support patients, or there are resources to teach self-management skills. However, in many centers, these resources are constrained or unavailable.

Growth in Internet access and proliferation of smartphones has created an opportunity to re-engineer cancer care delivery. Eighty-eight percent of adults in the US had web access and 77% had a smartphone in 2016. Although adoption is lower in the elderly and the poor, use is rising, and many use the internet to manage their health.[20,21] Mobile phones in general, and web access more generally, extend capacity for patient-clinician communication to optimize symptom management beyond the confines of a face-to-face encounter.[22] Patient engagement has been called the “blockbuster” drug[23,24] of the 21st century based on the recognition that motivated and activated patients have improved well-being and consistently achieve better health

	**Smartphone[18]**		**Web access[19]**	
Population	2010	2016	2010	2016
All adults	33%	77%	76%	88%
Age 65+	17%	54%	43%	64%
Rural	26%	67%	69%	81%
<$30,000 income	24%	64%	61%	79%
Black	29%	72%	68%	85%

outcomes.[25,26] Strong theoretical foundations from social cognitive theories of self-efficacy[27,28] and the chronic care model[29] support the importance of patient engagement[30-34] as a strategy to minimize the morbidity of cancer treatment.

Electronic symptom tracking and feedback is a promising strategy to improve symptom control. Electronic patient reported outcome (ePRO) monitoring of cancer symptoms has been shown to decrease symptom burden,[35] improve quality of life, reduce acute care[36] and even extend survival.[37] There is evidence to support two primary mechanisms of action. First, systematic reporting may activate patients to be more knowledgeable and effective at self-management.[38] This aligns with self-efficacy theory. Second, systematic reporting can trigger between-visit clinician actions that improve symptom control. This enhanced communication, facilitated by technology, could make health care more responsive to patient needs.[39,40]

Critical knowledge gaps will prevent successful implementation of ePRO systems in oncology practice. The NCI has invested in the development of measurement tools to facilitate symptom reporting such as the PRO-CTCAE (Patient-Reported Outcomes version of the Common Terminology Criteria for Adverse Events), a patient-reported outcome measurement system to capture symptomatic adverse events in cancer patients.[41-43] This item-bank enables symptom reporting and tracking using a consistent set of validated metrics for both clinical trials and routine care. The PRO-CTCAE, access to web-technology and the rapid proliferation of EHRs have created a context that is ripe for scaling a more proactive approach to cancer care delivery such as ePRO tracking. However, 4 critical knowledge gaps remain that will prevent successful implementation of ePROs to improve symptom management. They are:

1. **The ePRO effectiveness evidence base is limited to major cancer centers.** Evidence supporting the efficacy of ePRO symptom management stems largely from clinical trials and effectiveness studies conducted in large well-resourced cancer centers.[44,45] It is unknown whether the benefits found in these studies are generalizable to cancer care in rural, small and community-based settings, and evidence for their effectiveness and the adaptations necessary to make ePROs successful in these contexts is needed.

2. **ePRO systems are not fully integrated with EHRs.** While ePROs have demonstrated effectiveness without being fully embedded into the EHR,[46,47] integration would dramatically improve secure patient and clinician access to symptom reports and clinical utility of ePRO systems. Without integration, patients and clinicians must access separate systems to view symptom reports and cannot easily take actions such as ordering a medication or coordinating care with another relevant provider.[48] Full integration of ePRO systems into the EHR would facilitate secure two-way exchange of information and the ability to track symptoms, convey appropriately tailored educational materials, provide information about expected symptom profiles, send alerts, take actions, and coordinate care.

3. **ePRO systems have not leveraged demonstrably effective symptom coaching strategies.** Early ePRO research focused on feasibility, metric development and overcoming technological and regulatory obstacles. The efferent limb of the ePRO feedback loop has

received considerably less attention.[49,50] First, there has been limited work developing the information content that these systems deliver to help patients cope.[48,51] Where evidence-based symptom management tools exist, they are not fully leveraged.[52] Similarly, giving patients feedback about the extent to which their symptom profiles are typical or deviate from what is expected can provide reassurance or alert them to escalate treatment or seek help.

4. **Insufficient attention to implementation strategies will compromise the impact of ePROs.** There are myriad examples of effective health care interventions that do not realize their potential for impact because of insufficient attention to implementation.[53-55] ePRO systems require a shift in the traditional orientation of clinicians which typically confines symptom assessment to clinical encounters. There is inadequate knowledge about the implementation strategies that are facilitators of successful ePRO systems. How much training do patients require to engage in self-reporting? How much reinforcement is necessary? What level of training do clinic support staff, nurses, and clinicians require? What is the optimal design of dashboards to facilitate review of and acting on ePRO symptom reports? Established implementation science frameworks exist but have not been applied to ePRO systems.

A multi-disciplinary team of investigators from 6 health systems have formed the **S**ymptom Management **IM**plementation of **P**atient **R**eported Outcomes in **O**ncology **(SIMPRO)** Research Center. SIMPRO will use functioning ePRO prototypes to create and refine the **e**lectronic **sy**mptom **m**anagement system eSyM. eSyM is the name of the platform the team will refine, integrate, implement, and evaluate. eSyM addresses each of the 4 evidence gaps noted above by:

1. **Implementing** eSyM in cancer centers in small, rural, or community-based systems.
2. **Integrating** eSyM into the EHR of the predominant vendor used nationwide.
3. **Leveraging** evidence-based tools, patient engagement, and population management.
4. **Executing** this work using the Consolidated Framework for Implementation Research (CFIR, see figure below)[56] across all phases to maximize the chances that eSyM and similar systems achieve their intended goals and decrease the morbidity of cancer treatment at a population level.

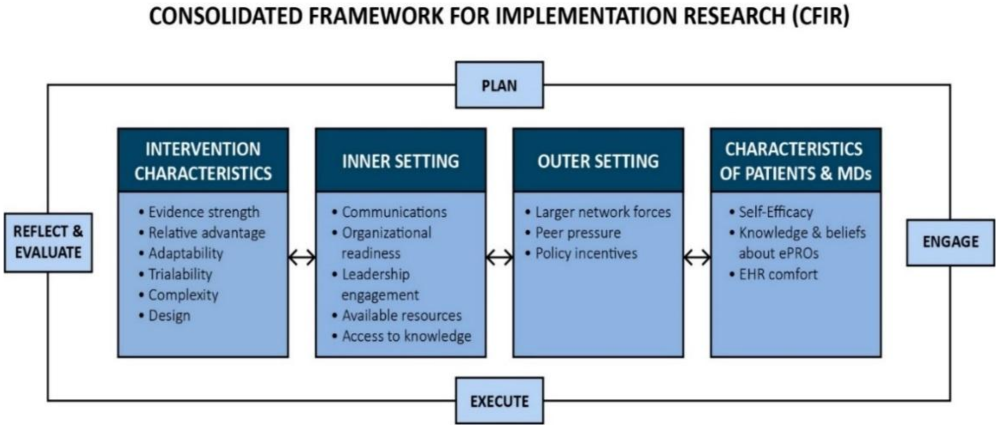

Using CFIR as a guide, we will utilize the plan-engage-execute-evaluate cycles across all aims.

3.0 Objectives

Overall Research Goals are: (1) to create and refine eSyM, a reporting and management system that integrates ePROs with the EHR; (2) to evaluate the impact of eSyM on patient outcomes, treatment delivery, and healthcare system utilization using a pragmatic cluster randomized study design; and (3) to undertake a systematic, deliberative approach to implementation to allow for the identification of barriers and facilitators that contribute to the adoption and sustainability of eSyM in routine oncology care.

Aim 1: Adapt existing ePRO symptom management systems and integrate them into the EHR and routine clinical workflow at six health systems. Specifically:

- Aim 1a. Obtain patient, clinician, staff, and leadership input on ePRO form and function
- Aim 1b. Refine the content and algorithms for self-management, alerts, and feedback
- Aim 1c. Develop ePRO training materials for patients, clinicians, and staff
- Aim 1d. Pilot an ePRO symptom manager at test and study sites and prepare an implementation strategy

Aim 2: Determine the effectiveness of an EHR-integrated ePRO symptom management system on health outcomes. Specifically:

- Aim 2a. Healthcare utilization, measured by the need for emergency and acute care
- Aim 2b. Impact on cancer care delivery, specifically chemotherapy treatment duration and delays
- Aim 2c. Patients' outcomes, indicated by levels of self-efficacy and symptom burden
- Aim 2d. Patients' satisfaction with their cancer care

Aim 3: Evaluate the facilitators and barriers to implementation of an EHR-integrated ePRO symptom management system from the patient, clinician, and organizational perspectives. Specifically:

- Aim 3a. Patient adoption (including program feedback and experiences via qualitative interviews), clinician utilization, and their perspectives on appropriateness and acceptability
- Aim 3b. The sustainability of ePRO symptom management within a health system
- Aim 3c. Penetration and scalability of ePROs for symptom management
- Aim 3d. Extent of adaptation of ePRO systems over the course of the implementation process

3.1 Hypothesis

Detailed hypotheses per aim can be found in the stats section of the protocol. Overall, investigators hypothesize that eSyM will enable patients to be more effective stewards of their own care and enable their clinicians to anticipate problems and intervene to manage symptoms before they escalate.

4.0 Inclusion and Exclusion Criteria or Activity Population

The eligibility criteria/activity population vary for each activity. See section 5.0 for details.

5.0 Protocol Activities

There are 5 protocol activities, each detailed in section 5.0.

- **Activity 1:** Obtain stakeholder feedback from patient advisory councils, health system leaders, clinicians, clinic support staff/administration, and IT/Informatics
- **Activity 2:** Build and deploy eSyM and finalize training materials based on findings from stakeholder engagement
- **Activity 3:** Pilot test eSyM
    - Activity 3a: eSyM usage
    - Activity 3b: User acceptability testing
    - Activity 3c: Medical record abstraction
- **Activity 4:** Pragmatic stepped-wedge cluster randomized trial
    - Activity 4a: eSyM usage
    - Activity 4b: SASS questionnaire
    - Activity 4c: Medical record abstraction
    - Activity 4d: Patient qualitative interviews
- **Activity 5:** Integration of eSyM data to develop algorithms to estimate the risk of experiencing an outcome, including, but not limited to, ED usage and hospitalization among cancer patients

<u>Activity 1:</u> Obtain stakeholder feedback from stakeholders, including (but not required) patient advisory councils, health system leaders, clinicians, clinic support staff/administration, and IT/Informatics

Brief description of activity: Before eSyM go-live, study team members from each site will solicit input via emailed survey, remote meetings and/or in-person meeting on the use of ePROs in oncology from stakeholders to obtain input regarding adaptation, anticipated challenges, and implementation (questions will be selected from the CFIR, AIM/IAM, NOMAD and CSAT Question Banks – see Appendix A G, J, and Y) and follow-up probes. The study team may adapt or create de novo questions as needed.

At least 30 days after eSyM go-live and on an ongoing basis, we will evaluate the implementation process at each of the sites with a focus on adoption, appropriateness, acceptability, sustainability, penetration, and scalability. We will do this using a combination of methods including evaluating medical record (and/or local cancer registry) reviews (see activities 3c and 4c), eSyM usage reports (see activities 3a and 4a), SASS questionnaire responses (see activity 4b), and feedback from emailed surveys and/or discussions with health system leadership, clinicians, clinic support staff, and informatics/IT staff (activity 1). Stakeholder questions will be selected from the CFIR, AIM/IAM, NOMAD and CSAT Question Banks – see Appendix A G, J, and Y) and follow-up probes. The study team may adapt or create de novo questions as needed.

Human Subjects Research Category (NHSR, exempt, expedited, full review): <u>Exempt</u> because the stakeholders at each site are acting in their normal business capacity and this is a negligible risk activity.

Informed Consent: <u>Waiver of informed consent</u>. Because eSyM will be implemented at each site *for use in routine clinical care and because this is a quality improvement (QI) activity*, it is necessary to consult each site's stakeholders during ePRO design and implementation. This activity is negligible risk.

Activity 1 Population:

- Age ≥ 18 years
- The potential stakeholders are patient advisory council members, health system leaders, clinicians, clinic support staff/administration, and IT/Informatics staff.

Number of subjects (per site and overall):

- Approximately 5-25 patient advisory council participants per site (30-150 patient advisory council participants overall)
- Approximately 4-10 health system leaders per site (24-60 health system leaders overall)
- Approximately 60 clinicians, clinic support staff/administration, and IT/Informatics staff per site. (360 clinicians, clinic support staff/administration, and IT/Informatics staff overall)

- We anticipate sending emailed surveys and/or presenting 2 separate times at approximately 6 meetings per site (a thoracic surgery, thoracic medical oncology, gynecological surgery, gynecological medical oncology, GI surgery, and GI medical oncology meeting). If 10 people participate from each setting, then we will collect data from approximately 120 people per site which totals 720 participants study-wide.

  ***Total number of stakeholder participants through surveys and meetings can be larger or smaller depending on availability.*

When, where, and how potential subjects will be recruited: The study PI at each site or his/her designee will send email surveys and/or meet with stakeholders individually, will get on upcoming meeting agendas, and/or will convene ad hoc meeting(s).

Materials that will be used to recruit subjects: See sample email in Appendix K and/or L. Each site may modify to meet their needs.

Duration of subject's participation in the study: If done via emailed survey (REDCap): The emailed survey will take approximately 15 minutes to complete. If interviewed in-person or remotely: Approximately 1 hour, the length of a meeting.

Duration anticipated to enroll all study subjects: We anticipate that it will take approximately three months prior to the go-live at each site to complete all initial stakeholder engagement. Implementation evaluation will occur throughout the five-year project period with a designated 1-year post-implementation survey/interview conducted at all sites.

Study design: Discussion guides and/or question slide decks will be used to facilitate stakeholder engagement.

Description of all research procedures being performed: The study PI (or designee) at each site will obtain feedback from the stakeholders at their site; feedback will be collected via emailed survey, and a meeting to be held one-on-one, in group settings, or remotely via writing, discussion, or handheld polling devices. All sites will be responsible for maintaining a list of stakeholders who will be invited to provide feedback.

The eSyM stakeholder interview guide (see appendix Y) may include selected items from CFIR, AIM, NOMAD, and CSAT (see appendices A, G, J, and Y). The eSyM questionnaire may include selected items from CFIR, AIM, NOMAD, and CSAT and will be sent stakeholders to be completed in REDCap. The study team may adapt or create de novo questions as needed.

In person and/or remote follow-up meetings will be conducted in group or one-on-one settings. Before any meetings may be conducted, all discussion facilitators must be trained by the Overall PI Deb Schrag or her designee on how to conduct the discussion; facilitators will be taught how to ask questions in a non-leading manner, how to respond to answers in a non-biased fashion, and how to ask appropriate follow up probe questions as/if needed. All discussion facilitators

will utilize the interview guide developed by the study team and probe as needed. A member of the team will take notes of all discussions and all discussions will be audio-recorded when possible. Clicker questions may also be used to collect feedback from stakeholders during larger group meetings. Feedback will also be ascertained through email and one-on-one discussions; written notes will be categorized and summarized for study analysis purposes.

All feedback collected will be submitted via REDCap or emailed to the coordinating center (Dana-Farber) for synthesis, summarization, and transcription as needed. Feedback may be collected in an identifiable fashion. For example, the CIO's comments may be attributed to "the CIO."

Monitor subjects for safety or minimize risks: Not applicable.

What data will be collected and how: Feedback and demographic information will be collected from stakeholders at each site regarding stakeholder views on adaptation, anticipated challenges, and implementation of ePRO (electronic patient reported outcomes).

Long-term follow-up: Stakeholders will be consulted and kept informed throughout the study period.

Activity 2: Build and deploy eSyM and finalize training materials

Brief description of activity: Using all of the feedback collected during pervious activities, the study team will finalize the content and build the eSyM system.

Human Subjects Research Category (NHSR, exempt, expedited, full review): Not Human Subjects Research.

Informed Consent: Not applicable.

Tasks that will be completed by the study team:

- Finalize eSyM's specifications:
    - prompt patients to report symptoms at user-defined intervals
    - track symptom profiles over time in via graphs
    - trigger delivery of symptom coaching in response to symptoms
    - alert patients to contact their clinicians in response to severe symptom reports
    - alert clinician about patients with severe symptoms
    - enable creation of dashboards that facilitate symptom burden monitoring of user-defined patient cohorts
    - use MyChart/Epic to securely access patient data
    - allow a proxy to report on behalf of a patient
    - eSyM will be integrated in the EHR and patient portal so that clinicians can expediently review and respond to ePROs with access to complete health records including medications, labs and visit notes with minimal disruption to workflow and with reliable record keeping
- Finalize the algorithms that control what eSyM does in response to user inputs.
- Finalize eSyM's patient-facing content.
- Finalize eSyM's clinician-facing content.
- Obtain necessary permissions or licensing agreements.
- The informatic team will finalize the eSyM build based on the specs provided by the study team.
- Each site may customize where allowed (example, where permissible, sites may brand their instance of eSyM with their own logos).
- Finalize eSyM training materials (see appendices AA through KK):
    - Patient-facing training materials (when and how to use eSyM).
    - Clinician-facing training materials (when and how to use eSyM).
    - Clinical staff-facing training materials (how to teach a patient to use eSyM).
- Depending on initial data and eSyM adoption rates, the study team (or a designee) may text, call, portal message and/or email patients to remind them to use eSyM.

Here is a sample of what the specifications for the build will look like:

Example of the Component Tools that Support eSyM for Diarrhea.

MODIFIED PRO-CTCAE SURVEY QUESTION

In the last 24 hours, how OFTEN did you have LOOSE OR WATERY STOOLS (DIARRHEA)?

0: Never | 1: Rarely | 2: Occasionally | 3: Frequently | 4: Almost constantly

DEPLOYMENT RULES & ADAPTATIONS

	MEDICAL ONOCLOGY	SURGERY
**Starting point**	1 day after chemo start	1 day after discharge
**Frequency**	2/week x 24 weeks	3/week x 2 weeks then 2/week x 2 weeks then 1/week x 4-8 weeks

SYMPTOM CLASSIFICATION (threshold for action – will depend on the symptom)

ACTION	FREQUENCY RESPONSE
**No intervention IF:**	= 0
**Symptom management advice IF:**	= 1 or 2 or 3
**Alert clinical team via InBasket IF:**	= 4

SYMPTOM EDUCATION FOR PATIENTS (selected examples)

Eat small meals that are easy to digest. Eat 5 or 6 small meals each day instead of 3 big meals. Choose foods that will help with diarrhea, such as applesauce, bananas, crackers, cream of wheat, eggs, toast, oatmeal, peanut butter, boiled potatoes, and rice.
Drink more each day. Drinking more won't stop the diarrhea, but it will help replace fluids you are losing to prevent dehydration. Most people who have diarrhea need 8 to 12 cups a day. Clear broth, water, tea, oral rehydration/electrolyte drinks (e.g., Pedialyte®), juice, and soda are good choices.
Take medications your team may have prescribed such as Imodium or Lomotil.
Avoid these foods: Some foods can make diarrhea worse. Don't have dairy, such as milk, cheese, and sour cream. Try "lactose-free" products instead. Don't eat spicy, greasy, or fried foods.
Call your cancer team if: You feel lightheaded, dizzy, or faint. These are symptoms of dehydration. You develop a fever of 100.5 F or higher. Your stool looks black or bloody. You are experiencing diarrhea that wakes you up from sleep at night.

SYMPTOM MANAGEMENT SUPPORT FOR CLINICIANS (selected examples)

Alert the clinician whenever a severe symptom is reported via InBasket.
Add the patient to a color-coded report of patients reporting symptoms in the last 7 days.
Symptom **responses visible in patient EHR** (e.g., snapshot report).

For a full list of symptoms, see the PRO-CTCAE bank (Appendices H & I); we will prioritize the symptoms below. PRO-CTCAE items may be modified, as needed (e.g., symptom lookback period):

eSyM Questionnaire Items		
	**Required**	**Optional**
**All Patients: Medical Oncology and Surgery**	Anxiety<br>Constipation<br>Fatigue<br>Pain<br>Poor Appetite<br>Nausea<br>Shortness of Breath<br>Trouble Drinking Fluids<br>Vomiting	Bleeding<br>Coughing<br>Difficulty Concentrating<br>Difficulty Sleeping<br>Difficulty Swallowing<br>Dizziness<br>Feeling Discouraged<br>Feeling Sad<br>Fever

	Overall Wellbeing<br>Physical Function	Hand-Foot Syndrome<br>Headache<br>Heart Palpitations<br>Heartburn<br>Itching<br>Mouth/Throat Sores<br>Swelling<br>Wheezing
**Medical Oncology**	Diarrhea<br>Numbness and Tingling<br>Rash	Painful Urination
**Surgery**	Painful Urination<br>Wound Discharge<br>Wound Redness	Diarrhea<br>Numbness and Tingling<br>Rash

<u>Here is a sample of what a patient-facing eSyM screen might look like:</u>

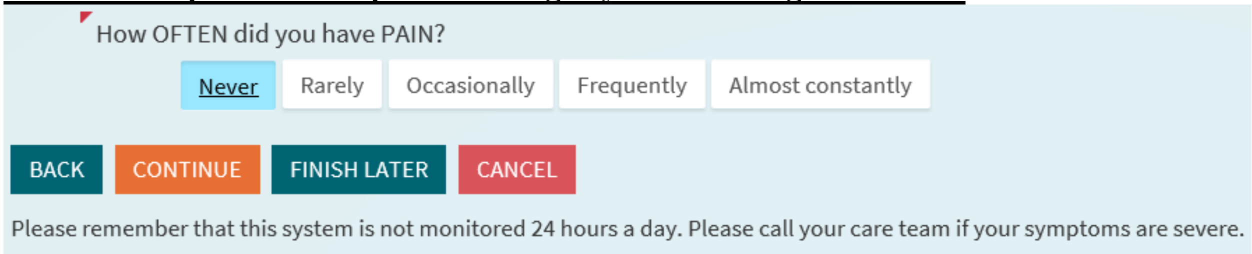

<u>Here is a summary of the eSyM functionalities that will be designed and built:</u>

**The main patient-facing tools will include the following (accessed via computer or app):**
1. **Alerts** → Reminders about when to complete PRO reporting<br>2. **Symptom Reporting** → Surveys that will allow patients to report outcomes for modified PRO-CTCAE items<br>3. **Visualizations** → Display previously reported PROs<br>4. **Education** → Evidence-based symptom management tools (see appendix T)
**The main clinician/staff-facing tools will include (accessed via computer or app):**
1. **Messaging** → Message notifying when critical PROs have been reported<br>2. **Visualizations** → Display previously reported PROs for a given patient, highlighting critical symptoms<br>3. **Reports** → Display patients who are enrolled in the program, view results of multiple patients, and identify patients who did not report PROs on schedule

<u>Activity 3: Pilot test eSyM</u>

Brief description of activity: eSyM will be UAT/pilot tested at up to 6 sites. The primary purpose of UAT/pilot testing is for the research team to observe patients, clinicians, and staff interacting with the new system, identify challenges, and iteratively refine the system, training materials, or clinic workflow prior to the launch of the full-scale pragmatic stepped-wedge cluster randomized trial.

SIV (Site Initiation Visit): The SIV, protocol, and eSyM training is critically important to the success of the pilot study. All involved staff (on the research team and clinical teams) will be required to complete training using the materials and methods developed via Activity 2. Training activities include:

- Training clinicians and clinic staff at each site on new clinic workflow SOPs.
- Training clinicians and clinic staff on how to get a patient set up with eSyM.
- Training each user audience (clinicians and patients) how to use their eSyM interface.
- Training informatics/IT staff at each site how to support and maintain eSyM.

*** See appendices AA through LL for study resources. Please note – training materials and project resources will be routinely updated and branded to meet site needs.*

Activity 3 Population:

- Age ≥ 18 years
- Priority population will be patients who meet one of the following:
    - Suspected thoracic cancer AND is inpatient following thoracic surgery.
    - Suspected gastrointestinal cancer AND is inpatient following gastrointestinal surgery.
    - Suspected gynecologic cancer AND is inpatient following gynecologic surgery.
    - Diagnosis of thoracic cancer AND scheduled to start a new treatment plan for thoracic cancer.
    - Diagnosis of gastrointestinal cancer AND scheduled to start a new treatment plan for gastrointestinal cancer.
    - Diagnosis of gynecologic cancer AND scheduled to start a new treatment plan for gynecologic cancer.
- Total population allowed to use eSyM:
    - Any patient at any participating site.

  ***Please note – Patients undergoing thoracic, gynecologic, or gastrointestinal surgery may not be diagnosed with cancer. These patients are still eligible for eSyM usage, questionnaire completion, and medical record abstraction.*

Activity 3 population will be operationalized as follows:

- To determine if a patient has a diagnosis of one of the above cancer types, use ICD-10 diagnosis codes: 15.0-16.99 (esophago-gastric) C17.0-C21.9 (small int. colorectal) C22.0-24.9 (hepatobiliary) C23-C25.9 (pancreas), C34-34.9: lung, C53.0-53.9 (cervix) C54-54.9 (uterine) and/or C56-57.9 (ovarian). When it comes time to execute, this list may be modified. CPT and procedure codes and EPIC's OPTIME operating room scheduling module will be used to determine if a patient is scheduled for a priority surgery.

Mode of Participation: Patients will have a *choice* of their preferred mode of eSyM participation and switching can be accommodated. Patients with a smart phone will be offered that approach first. Alternatives include participation in eSyM via any web-enabled device (laptop/tablet/desktop). Patients may designate a caregiver willing to elicit their symptoms and

report responses on their behalf (proxy reporting). This flexibility facilitates the intervention's reach to frail patients who may be those most likely to benefit. As needed, patients will also be offered eSyM training through in-person, phone, and/or virtual visits.

Number of subjects (per site and overall): For Activity 3, user acceptability testing (UAT) will be done with up to 390 patients from up to 6 participating sites. At the lead site (Dana-Farber), we anticipate conducting UAT with 90 participants distributed as follows: 15 patients from each cancer/type modality combination (15 thoracic surgical, 15 gynecologic surgical, 15 GI surgical, 15 thoracic medical oncology, 15 gynecologic medical oncology, and 15 GI medical oncology). We anticipate that at each of the 5 remaining participating sites, each site will conduct UAT with up to 60 participants distributed as follows: 10 patients from each cancer/type modality combination (10 thoracic surgical, 10 gynecologic surgical, 10 GI surgical, 10 thoracic medical oncology, 10 gynecologic medical oncology, and 10 GI medical oncology). Protocol does not mandate equal distribution of participants among participating sites. If a site is running behind or facing lower than anticipated accrual to the activity, then the other sites may enroll additional participants to make up the difference until the study wide UAT goal has been reached. The numbers referenced above are meant to serve as an estimate and sites may suspend or continue UAT as needed.

Materials that will be used to recruit subjects: None.

Duration anticipated to enroll all study subjects: We anticipate that it will take three months prior to eSyM launch at each site to enroll all UAT/pilot participants, but the actual duration may vary.

Monitor subjects for safety or minimize risks: The risk to participants is minimal with the primary risk being loss of confidentiality/privacy. To monitor the risk of loss of confidentiality/privacy, the informatics/IT team will routinely monitor eSyM and investigate inquiries from study teams. Furthermore, patients who report severe symptoms will be prompted to call their clinic immediately, and clinicians will receive in-basket notifications in the Epic EHR of the severe symptom report.

Activity 3a: eSyM Usage

Human Subjects Research Category (NHSR, exempt, expedited, full review): NHSR/QI
Because eSyM will be implemented at each site *for use in routine clinical care and because this is a quality improvement (QI) activity, this is not human subjects research*.

Informed Consent: Waiver of consent. When a patient becomes eligible for eSyM, he/she/they will receive an automated welcome message, which includes an electronic disclaimer that contains important information about the purpose of eSyM, how/when it should be used, and what it does and does not communicate to their care team (see Appendices N & Q).

Methods that will be used to identify potential subjects: Access to eSyM will be automated. EPIC will automatically deliver the invitation to use eSyM to all patients defined above (See Section: "Activity 3 population will be operationalized as follows").

Duration of subject's participation in the study: Per protocol, eSyM usage continues for up to 60-180 days from their trigger event (i.e., new chemotherapy treatment plan and/or surgery), but extended use of eSyM is at the discretion of the site and each patient participant.

What data will be collected and how:
- Participant responses to the symptom reporting questions within eSyM will be collected.
- Clinician responses to symptom reports will be collected.
- Data on eSyM usage by all user types will be collected.

Long-term follow-up: We will follow patients for 1-year after the trigger event (i.e., new chemotherapy treatment plan and/or surgery) for outcomes data captured via medical record abstraction.

Activity 3b: User Acceptability Testing (UAT)

Informed Consent: Waiver of documentation of consent. Before observing the patient using eSyM, the patient will be provided with a letter (see appendix O) with the elements of informed consent, as well as the option not to be observed. They will be notified that participation is completely voluntary and can be stopped at any time for any reason. This activity is minimal risk.

Methods that will be used to identify potential subjects: Under a HIPAA waiver, study staff will look in the scheduling views of the electronic medical record and scheduling systems to identify potential participants. Study staff may also query Epic, administrative/operations/billing databases, order entry databases, and/or cancer registry databases to identify potential participants. Study staff may also accept potential patient referrals from site clinicians. Purposive sampling based on data from the electronic medical record (e.g., demographics, cancer stage, number of recent hospitalizations, number of prescription drugs) will be used to ensure that perspectives of diverse patients are included. Ideally, sites will use the developed eSyM reporting workbench reports in Epic to automatically pull a patient list for UAT.

When, where, and how potential subjects will be recruited: The clinical/study staff will approach the patient to see if they would be interested in participating in UAT. If a patient is interested in participating, the site will present the participant with a study letter and then begin observations. Patients may be approached in-person, via phone, via email or through a virtual visit.

Description of procedures being performed for UAT: Surgical patients will be set up to use eSyM at the time of surgical discharge (or at the site's discretion). Medical oncology patients will be set up to use eSyM at the time of their first chemotherapy dose visit (or at the site's discretion). The study staff will observe the patient accessing eSyM, the patient being trained on how to use eSyM, the patient using eSyM to do their first symptom reporting session.

Duration of subject's participation in the study: Per protocol, UAT observation will last approximately 30 minutes or less.

What data will be collected and how: The study staff will write down their observations as well as staff/patient feedback (see appendix P).

Study design: User acceptability testing.

Long-term follow-up: None. UAT is a one-time 30-minute session.

Activity 3c: Medical record abstraction

Informed Consent: Waiver of consent to conduct medical record (and/or local cancer registry) reviews/Epic queries on all patients in the UAT/pilot to ascertain demographics and outcomes.

Methods that will be used to identify potential subjects: Automated and/or manual medical record (and/or local cancer registry) review will be done on all UAT/pilot patients.

When, where, and how potential subjects will be recruited: All participants in the UAT/pilot will undergo medical record (and/or local cancer registry) reviews. The medical record (and/or local cancer registry) reviews will be accomplished using both automated data extraction and manual data abstraction.

What data will be collected and how:

- Study-related health information and outcomes will be collected via medical record (and/or local cancer registry) review (automated and manual).
- Data will be submitted to the coordinating site (Dana-Farber) via REDCap or SFTP.

Study design: Medical record abstraction (both manual and automated).

Long-term follow-up: The patients' medical record may be reviewed for outcomes for up to 1-year after the trigger event (i.e., new chemotherapy treatment plan and/or surgery).

Activity 4: Pragmatic stepped-wedge cluster randomized trial

Brief description of activity: A multisite, pragmatic stepped-wedge cluster randomized trial will be conducted in order to determine the effectiveness of eSyM on health outcomes including: healthcare utilization, measured by the need for emergency and acute care; impact on cancer care delivery, specifically chemotherapy treatment duration and delays; patients' outcomes, indicated by levels of self-efficacy and symptom burden; and patients' satisfaction with their cancer care.

The trial involves patients in five ways:

1) Patients assigned to eSyM (eSyM+) will report their symptoms via the eSyM questionnaires delivered through MyChart. Patients are strongly encouraged to report their symptoms at home/between clinic visits with eSyM. Study teams and clinicians may elect to ask eSyM-eligible patients to complete eSyM questionnaires in the clinic via a table, computer, and/or mobile device if patients have difficulties reporting or decline to report at home. Site study teams should consult the central study team before they initiate this workflow.

2) A subset of eSyM+ patients will be asked to complete a research questionnaire called the "SASS Questionnaire (eSyM+ or eSyM+ Non-Responder version)" asking about their **S**elf-efficacy, **A**ttainment of information needs, **S**ymptom burden, and **S**atisfaction with care (see PROMIS, CAHPS, IAM/AIM question banks – Appendices C through G). The eSyM+ version is for patients who were assigned to eSyM and completed at least one eSyM questionnaire. The eSyM+ Non-Responder version is for patients who were assigned to eSyM but never completed an eSyM questionnaire. The questionnaires will stop being administered once a minimum of 1,980 total surveys have been received in accordance with the following breakdown:

**SASS Questionnaire** accrual numbers							
	Surgery			Medical Oncology			Totals
	eSyM+ Version	eSyM- Version	eSyM+ Non-Responder Version	eSyM+ Version	eSyM- Version	eSyM+ Non-Responder Version	
Site 1	75	75	15	75	75	15	330
Site 2	75	75	15	75	75	15	330
Site 3	75	75	15	75	75	15	330
Site 4	75	75	15	75	75	15	330
Site 5	75	75	15	75	75	15	330
Site 6	75	75	15	75	75	15	330
Totals	450	450	90	450	450	90	1,980

3) Select patients NOT assigned to eSyM (control group, eSyM-) will also be asked to complete the "SASS Questionnaire (eSyM- version)" (see Appendices U-X). Both Drug Therapy and Surgical Recover Experience eSyM- versions will not ask questions evaluating eSyM as the cohort will not be exposed to eSyM at the time of the survey. The

questionnaire will stop being administered once a minimum of 1,980 total surveys have been received in accordance with the above breakdown. ** *Total number of SASS participants through surveys can be larger or smaller depending on availability.*

4) A small subset of eSyM- and eSyM+ patients will be invited to take part in follow-up qualitative interviews. See table below for recommended recruitment accrual. The interviews will be conducted following a developed interview guide (Appendix RR). Patients may or may not have previously completed SASS or eSyM questionnaires. Interviews will continue until thematic saturation is reached, or until 100 interviews are completed, whichever is first.

**Patient Qualitative Interviews**	
**Site**	**Recommended Minimum Patient Interview Accrual**
Site 1	4
Site 2	4
Site 3	4
Site 4	4
Site 5	4
Site 6	4
**Total**	**24**

Total accrual can be larger or smaller depending on patient availability and interest. No more than 100 interviews will be conducted study-wide. Protocol does not mandate equal distribution of participants among sites.

5) Regardless of whether a patient is assigned to eSyM+, eSyM-, or eSyM+ Non-Responder, and regardless of whether a patient completes the SASS Questionnaire or not, we are requesting a waiver of consent and HIPAA waiver to conduct medical record (and/or local cancer registry) reviews/Epic data queries on all patients in the denominator (for the definition of "denominator", please see the section "how to operationalize the denominator" below) to ascertain demographics and outcomes. Because eSyM has QI implications and is part of routine clinical care, we must be able to compare responders with non-responders and eSyM+ with eSyM- and the combinations thereof to inform implementation beyond this initiative.

Informed Consent:

Activity 4a: eSyM Usage (eSyM+): NHSR/QI.
Because eSyM will be implemented at each site *for use in routine clinical care and because this is a quality improvement (QI) activity, using eSyM is not human subjects research, and therefore does not require consent.* When a patient becomes eligible for eSyM, he/she/they will receive an automated welcome message with a disclaimer that contains important information about the purpose of eSyM, how/when it should be used, and what it does and does not communicate to their care team (see Appendices N & Q).

Activity 4b: SASS QUESTIONNAIRE PARTICIPANTS (eSyM+ , eSyM-, and eSyM+ Non-Responder): Waiver of documentation of consent.
Eligible patients will receive a study letter in-person, by email, and/or by postal mail asking them to consider participating in a questionnaire that asks about their self-efficacy, attainment of information needs, symptom burden, and satisfaction with care (see Appendix Z). This invitation will include the elements of informed consent (see Appendix R). The study letter will notify participants that the questionnaire is completely voluntary and can be stopped at any time for any reason. The letter will be sent from the health system where the patient receives care and signed by the site PI. For example, a patient at Dartmouth will receive a letter/email inviting participation from Dartmouth signed by Dr. Sandra Wong and a patient from West Virginia University will receive a letter/email inviting participation from Dr. Hannah Hazard. The letter includes a statement that survey completion has no influence or impact on a patient's medical care. This activity is minimal risk.

Activity 4c: MEDICAL RECORD REVIEW: Waiver of consent.
Waiver of consent to conduct medical record (and/or local cancer registry) reviews/Epic queries on all patients in the "denominator" to ascertain demographics and outcomes regardless of whether a patient is assigned to eSyM+ or eSyM- and regardless of whether a patient completes the questionnaire or not. Because eSyM has QI implications and is part of routine clinical care, we must be able to compare responders with non-responders and eSyM+ with eSyM- and the combinations thereof to inform implementation beyond this trial. The medical record review meets all the conditions to obtain a waiver of consent (i.e., not FDA-regulated, involves no more than minimal risk, waiver will not adversely affect the rights and welfare of the subjects, and activity could not be practicably carried out without a waiver).

Activity 4d: PATIENT QUALITATIVE INTERVIEWS: Written informed consent.
Eligible patients will receive a study letter in-person, by email, by Epic MyChart portal message, and/or by postal mail inviting them to participate in a qualitative interview that asks about their experiences with and feedback about the eSyM program and/or their care experience. This invitation will include a written consent form (see Appendices QQ & SS), which will be signed by the participant. Informed consent can be obtained through wet ink (returned in-person or via snail mail) or e-consent through REDCap. The provided study letter and consent form will notify participants that the interview is completely voluntary and can be stopped at any time for any reason. The consent form will also request patient approval for their contact information (name,

email, address, and phone number) to be shared with the coordinating center (Dana-Farber Cancer Institute) for the purposes of conducting the interviews. All patient qualitative interviews will be conducted by the study team at the coordinating center, who will reach out to the patient once the signed consent form is received. The study letter and consent form will be sent from the health system where the patient receives care and signed by the site PI. For example, a patient at Dartmouth will receive a letter/email inviting participation from Dartmouth signed by Dr. Sandra Wong and a patient from West Virginia University will receive a letter/email inviting participation from Dr. Hannah Hazard-Jenkins. The letter includes a statement that survey completion has no influence or impact on a patient's medical care. This activity is minimal risk.

Methods that will be used to identify potential subjects: The denominator will be identified algorithmically; eSyM/MyChart/REDCap will be programmed so that the correct patients will automatically receive an invitation to use eSyM. When manual support is needed, under a HIPAA waiver, study staff will run reports in Epic, administrative/operations/billing databases, order entry databases, and/or cancer registry databases.

Under a HIPAA waiver, study staff will run reports in Epic as part of the eSyM registry and/or to identify patients meeting eSyM registry criteria to identify medical and surgical patients for eSyM+, eSyM-, eSyM+ Non-Responder SASS questionnaires, and patient qualitative interviews. Select eSyM- patients will be identified and surveyed before eSyM's launch at their site but after the registry has been created, ensuring that they have never been exposed to the program but would have been eligible to use it. Patients eligible to complete the eSyM- questionnaire will be identified using the patient registry and/or a site-developed Epic report to identify patients who had a qualifying surgery or new chemo start within the last 6 months. Patients eligible to complete the eSyM+ and eSyM+ Non-Responder questionnaires will be identified using the registry after eSyM has been launched at their site and 30-60 days after their surgery or new chemotherapy. The eSyM+ SASS questionnaire will be distributed to patients who were assigned to eSyM and completed at least one eSyM questionnaire. The eSyM+ Non- Responder SASS questionnaire will be distributed to patients who were assigned to the eSyM program but never completed an eSyM questionnaire. Sites may begin distributing eSyM+ and eSyM+ Non-Responder questionnaires as soon as they are able to do so after go-live and will continue until a minimum of 75 eSyM+ surveys and a minimum of 15 eSyM+ Non-Responder surveys have been collected for the medical oncology and surgery versions. Patients eligible to participate in the qualitative interviews will be identified using Epic reports and invitations will be sent to patients meeting eSyM program eligibility. Patients may or may not have previously completed SASS or eSyM questionnaires. Interviews will be conducted until thematic saturation is reached, or until 100 interviews have been completed, whichever is reached first.

In addition, under a HIPAA waiver, email and postal addresses will be obtained for all eSyM+, eSyM-, eSyM+ Non-Responder and qualitative interview patients. Email and postal addresses will be utilized to share the SASS questionnaire invitation through REDCap and/or mailing (see Appendix Z) and to send the interview invitations and consent forms via email and/or mailing (see Appendix SS and QQ).

Activity 4 Population:

- Age ≥ 18 years
- Priority population will be patients who meet one of the following:
    - Suspected thoracic cancer AND is undergoing thoracic surgery.
    - Suspected gastrointestinal cancer AND is undergoing gastrointestinal surgery.
    - Suspected gynecologic cancer AND is undergoing gynecologic surgery.
    - Diagnosis of thoracic cancer AND scheduled to start a new treatment plan for thoracic cancer.
    - Diagnosis of gastrointestinal cancer AND scheduled to start a new treatment plan for gastrointestinal cancer.
    - Diagnosis of gynecologic cancer AND scheduled to start a new treatment plan for gynecologic cancer.
- Total population allowed to use eSyM:
    - Any patient at any participating site.

  ***Please note – Patients undergoing thoracic, gynecologic, or gastrointestinal surgery may not be diagnosed with cancer. These patients are still eligible for eSyM usage, questionnaire completion, and medical record abstraction.*

Defining and operationalizing the denominator:

- To determine if a patient has a diagnosis of one of the priority cancer types, use ICD-10 diagnosis codes: 15.0-16.99 (esophago-gastric) C17.0-C21.9 (small int. colorectal) C22.0-24.9 (hepatobiliary) C23-C25.9 (pancreas), C34-34.9: lung, C53.0-53.9 (cervix) C54-54.9 (uterine) and/or C56-57.9 (ovarian). When it comes time to execute, this list may be modified. CPT and procedure codes as well as EPIC operating room scheduling lists will be used to determine if a patient is scheduled for a priority surgery.
- Participants assigned to eSyM+ may be trained to use eSyM by their local clinic/study staff. Surgical patients may be trained to use eSyM at the time of surgical discharge (or at the site's discretion patients may receive training in how to use eSyM at a preoperative visit). Med Onc patients may be trained to use eSyM at the time of their first chemotherapy dose visit or at the time of a pre-treatment visit for chemotherapy teaching (at the site's discretion). Epic reports may also be used to identify eligible patients who are assigned or will be assigned to eSyM so that training of the eligible patients can be done by study research staff via telephone or email before or after beginning treatment or having surgery.
- Participants in the eSyM- cohort will meet priority population criteria and be seen at a participating site between 1/1/2018 and the site's eSyM go-live date. Participants in the eSyM+ cohort will meet priority population criteria and be seen at a participating site after their eSyM go-live date through March 31, 2024.

Mode of Participation: Patients will have a *choice* of their preferred mode of eSyM participation and switching can be accommodated. Patients with a smart phone will be offered that approach first, however all patients will be notified that access to eSyM is available as long as they can access the secure patient portal (MyChart). eSyM can be accessed securely through the MyChart portal on any web-enabled device. Alternatives to smart phone reporting include

participation in eSyM via any web-enabled device (laptop/tablet/desktop). Patients may designate a caregiver willing to elicit their symptoms and report responses on their behalf (proxy reporting). This flexibility facilitates the intervention's reach to frail patients who may be those most likely to benefit. The influence of mode of participation and reliance on self- versus proxy-reporting will be analyzed in multivariable models.

The denominator is defined above. Automated and/or manual medical record (and/or local cancer registry) review will be done on all patients in the denominator.
Number of subjects (per site and overall): For Activity 4, there will be a *minimum* of 6,048 eSyM+ patient users. Prespecified eSyM- accrual numbers can be found in Appendix TT (SAP). eSyM users and control patients can be from any of the 6 participating sites. We anticipate that each of the 6 hospitals in the stepped wedge trial will have a *minimum* of 900 patients. Protocol does not mandate equal distribution of participants among participating sites. The entire statistical analysis section is predicated on a *minimum* N=6048. Because the goal is broad-based systems-level implementation, we anticipate having much a higher N which will enable us to examine effectiveness in subgroups of interest.

When, where, and how potential subjects will be recruited: Once the population definition has been programmed into eSyM/ MyChart, the system will automatically deliver the invitation to use eSyM to the patients in the eSyM+ group (see randomization schedule figure below under the title "stepped wedge rollout"). eSyM- patients will be identified using Epic query reports. Study staff will deliver the invitation to participate in the SASS Questionnaire to all patients in the denominator (eSyM+, eSyM-, and eSyM+ Non- Responder) using the registry before and after eSyM launch until at least 1,980 have participated. Because different patients are reachable by different methods, patients may be contacted through email, postal mail, by phone, or in clinic inviting them to use eSyM and/or participate in the SASS Questionnaire.

There are six versions of the SASS Questionnaire: surgical experience eSyM+, eSyM-, and eSyM+ Non-Responder and drug therapy eSyM+, eSyM-, and eSyM+ Non-Responder. All versions contain validated PRO items (including PROMIS and CAHPs) and validated computer and internet use items from PEW. The eSyM+ versions contain additional items to evaluate usability and satisfaction with eSyM. The eSyM+ Non-Responder versions include additional questions to evaluate why eligible eSyM patients did not respond to the eSyM questionnaires. The SASS Questionnaire may be administered through REDCap, by phone, email, postal mail, or in clinic. Multiple methods of collection are allowed in order to minimize barriers to participation. As needed, patients will also be offered eSyM training through in-person, phone, and/or virtual visits.

The patient qualitative interviews will follow the interview guide in Appendix RR. The interview will ask questions regarding patients' overall experience with the program, suggested improvements they would make to the eSyM symptom questionnaire or the overall program, and any potential or real barriers to accessing or using the program. The interviews may take place by phone, via Zoom/video conference, or in-person. Multiple methods of collection are allowed to minimize barriers to participation.

Materials that will be used to recruit subjects: See Appendices M and N for the message that will be sent to patients inviting them to participate in the questionnaire and/or eSyM, respectively. As a reminder, participating in the SASS questionnaire or eSyM survey will not influence a patient's medical care in any manner. The purpose of these questionnaires is to help care teams learn about the best way to help patients cope with symptoms between visits (for medical oncology) and after surgery (for surgical patients).

See Appendices SS and QQ for the message and consent form that will be sent to patients inviting them to participate in the qualitative interview. Participating in the qualitative interview will not influence a patient's medical are in any manner. The purpose of these interviews is to gather direct patient feedback for study research staff about eSyM to inform future optimizations to the program.

Duration of subject's participation in the study:

1) Per protocol, eSyM usage continues for up to 60-180 days but can continue indefinitely at the site's discretion.
2) The SASS Questionnaire is a one-time, 20-minute survey administered 30-180 days after surgery or first dose of chemotherapy.
3) The patient qualitative interview(s) will last approximately 30 (but no more than 120) minutes; they will be administered any time after eSyM assignment at a time that is convenient for the subject.
4) All eSyM- control and eSyM+ intervention patients will be followed for outcomes for up to 1-year after the trigger event (i.e., new chemotherapy treatment plan and/or surgery).

Duration anticipated to enroll all study subjects: We anticipate that it will take five years to complete this activity.

Study design: Hybrid effectiveness-implementation stepped-wedge cluster randomized trial.

Description of all research procedures being performed:

a) Epic, administrative/operations/billing databases, order entry databases, and/or cancer registry databases may be queried to identify denominator patients under a HIPPA waiver.
b) Patients assigned to eSyM+ will be set up to use eSyM by their local clinic/study staff. Surgical patients will be set up to use eSyM at the time of surgical discharge (or at the site's discretion). Medical oncology patients will be set up to use eSyM at the time of their first chemotherapy dose visit (or at the site's discretion).
c) Select eligible participants will receive a message inviting them to participate in the SASS questionnaire 30-days after surgery or first chemo dose (Appendix Z).
d) Participants who participate in the SASS questionnaire and indicate that they wish to receive a gift card will receive $15 gift card. Gift cards will be mailed or emailed according to patient preference. Please note, emailed gift cards will come directly from Amazon.com or sent by a study research staff member.

e) Participants who participate in the qualitative interviews and indicate that they wish to receive a gift card will receive a $15 gift card. Gift cards will be mailed or emailed according to patient preference. Please note, emailed gift cards will come directly from Amazon.com or sent by a study research staff member.
f) All denominator patients (eSyM- control and eSyM+ intervention) will undergo medical record (and/or local cancer registry) reviews. The medical record (and/or local cancer registry) reviews will be accomplished using both automated data extraction and manual data abstraction under a HIPAA waiver.

Monitor subjects for safety or minimize risks: The risk to participants is minimal with the primary risk being loss of confidentiality/privacy. To monitor the risk of loss of confidentiality/privacy, the informatics team will routinely monitor eSyM reports and data extracts and investigate inquiries from study teams. Furthermore, patients who report severe symptoms will be prompted to call their care team immediately, and clinicians will receive in-basket notifications in the Epic EHR of the severe symptom report. All interview participants from outside of Dana-Farber Cancer Institute will provide written consent to have their information shared with the Dana-Farber research staff members who will contact them to conduct the interviews.

What data will be collected and how:

- Participant responses to the symptom reporting questions within eSyM will be collected.
- Patient and clinician eSyM usage reports will be collected.
- Patient-reported self-efficacy, attainment of information needs, symptom burden, and satisfaction with care will be collected via the SASS Questionnaire.
- Patient feedback on the eSyM program and experiences with care team and symptom management via qualitative interviews.
- Study-related health information and outcomes will be collected via medical record (and/or local cancer registry) review (automated and manual).
- Hospital/clinic characteristics will be collected.
- All data collected for this study will be submitted to the coordinating center (Dana-Farber Cancer Institute).

Long-term follow-up: The patient's medical record may be reviewed for outcomes for up to 1-year after the trigger event (i.e., new chemotherapy treatment plan and/or surgery).

<u>Activity 5:</u> Integration of eSyM data to develop algorithms to estimate outcomes, including the risk of ED usage and hospitalization, among patients with suspected or confirmed cancer

Brief description of activity: Patients receiving treatment for metastatic cancer have high symptom burdens and high rates of adverse events, including emergency department (ED) use and hospital admissions, which can impact patients' quality of life.[57,58] Some of these adverse events may be preventable if providers can match patients with appropriate outpatient services. However, to achieve this goal, we must improve our ability to prospectively identify the patients

at increased risk for near-term adverse events and most likely to benefit from intensification of outpatient care.

Several studies have used EHR data to predict risk of death in cancer patients.[59,60] However, efforts to date have several limitations. They rely on computationally-intensive methods that are difficult to implement in routine clinical practice. Little work has been done to develop models that predict adverse events which are actionable in the near-term. Existing risk-prediction models are susceptible to racial biases; little research has evaluated whether these models' calibrations may have racial biases.[61] And few efforts have incorporated newer data sources, such as ePROs, into risk prediction models. ePROs, which are now frequently collected in routine clinical care, may improve predictions.[62]

We aim to develop methods to predict which patients are at the highest risk for undesirable outcomes, including hospitalization and ED use. These predictions will lay the foundation for future work to develop targeted interventions to intensify outpatient care and reduce preventable adverse events. Specifically, we will use SIMPRO data (including demographics, conditions, medications, encounters, ePROS, etc.) to develop predictive models that are built suing machine learning clinical risk prediction techniques. Through these efforts, we seek to address a number of important questions, including how ePRO data affect model performance; how clinical risk scores perform relative machine learning algorithms; and whether there are differences in model performance among different patient subgroups (to determine whether predictive model calibrations based on EHR data may be intrinsically biased).

Using data from six health systems will allow us to evaluate algorithms to predict adverse events in diverse clinical settings

Specific Aims:

1. Develop algorithms to estimate the risk of adverse events, including ED usage and hospitalization, among patients with suspected or confirmed cancer who are treated in community settings.
2. Assess whether inclusion of ePROs improves algorithm performance and how predictions from clinical risk scores compare to predictions from computationally intensive machine learning approaches
3. Assess model performance among historically under-represented populations, including Black patients, and assess for evidence of racial bias in model calibration.

Human Subjects Research Category (NHSR, exempt, expedited, full review): Exempt

Informed Consent: Waiver of consent (retrospective data review).

Activity 5 Population: All patient-level data collected in Activity 4 will be utilized. To date, the SIMPRO consortium has collected more than 50,000 survey responses and EHR data for more than 13,000 medical oncology and surgical oncology patients across these systems. These data

present an opportunity to develop and test algorithms to predict adverse events in patients receiving care for a suspected or confirmed cancer.

Number of subjects (per site and overall): All patient-level data collected in Activity 4 will be utilized.

When, where, and how potential subjects will be recruited: Not applicable. Only previously collected patient-level data will be utilized.

Materials that will be used to recruit subjects: Not applicable. Only previously collected patient-level data will be utilized.

Duration of subject's participation in the study: Not applicable.

Duration anticipated to enroll all study subjects: Not applicable.

Study design: Retrospective data analysis project.

Description of all research procedures being performed: We will use data previously collected for the SIMPRO project to develop and evaluate the performance of predictive models for adverse events among patients with suspected or confirmed malignancy. The only new procedures will include novel analyses of existing data.

Monitor subjects for safety or minimize risks: Not applicable, only deidentified datasets will be utilized for predictive modeling work.

What data will be collected and how: Not applicable, previously collected data will be utilized.

Long-term follow-up: Not applicable.

6.0 Study-Wide Number of Subjects

	Activity 1 – Stakeholder Engagement				Activity 2	Activity 3- Patient Engagement	Activity 4 - Patient Engagement	Activity 5 – Predictive Modeling Algorithms
	Patient Advisory Council	Health system leaders	Clinicians and staff	Follow-up	Build eSyM	UAT/Pilot study	Pragmatic stepped-wedge cluster randomized trial	Integration of eSyM data
	Exempt, waiver of consent				NHSR	3a) NHSR<br>3b) Waiver of documentation of consent<br>3c) Waiver of consent	4a) NHSR<br>4b) Waiver of documentation of consent<br>4c) Waiver of consent<br>4d) Written consent	Exempt, waiver of consent
Study-Wide Accrual	30-150[a]	24-60[a]	360[a]	720[a]	n/a	390	6,048 (**minimum**)[d] [1,980 of whom will complete the SASS Questionnaire and 48 patient interviews]	n/a
Accrual per site	5-25[a,c]	4-10[a,c]	60[a,c]	120[a,c]	n/a	90[c] at Dana-Farber, and 60[c] at each of the other participating sites.	1,008[c] (**minimum**)[d] at each of the six participating sites [330 of whom will complete the SASS Questionnaire and 8 taking part in patient interviews]	n/a
	GRAND TOTAL MAXIMUM ACCRUAL ACROSS ALL STUDY ACTIVITIES PER SITE = 5,000<br>GRAND TOTAL MAXIMUM ACCRUAL ACROSS ALL STUDY ACTIVITIES STUDY-WIDE = 30,000							

[a] Number can be larger or smaller depending on meeting attendance and availability of staff.
[b] Interviews/surveys will continue until thematic saturation is reached.
[c] Protocol does not mandate equal distribution of participants among participating sites.
[d]The entire statistical analysis section is predicated on *minimum* N = 6048. Because the goal is broad-based systems-level implementation, we anticipate having much higher N which will enable us to examine effectiveness in subgroups of interest.

7.0 Study-Wide Recruitment Methods

Each activity utilizes slightly different recruitment methods. See section 5.0 for details.

8.0 Multi-Site Research

This is a multi-site study. The lead site and coordinating center for this protocol is Dana-Farber Cancer Institute. The regulatory sponsors are Dr. Deborah Schrag MD MPH (Co-Grant PI and Study Chair) and Dr. Michael Hassett MD MPH (Co-Grant PI and Coordinating Center Site PI). **All participating sites are required to adhere to the Data Safety and Monitoring Plan (DSMP) for this study (see Appendix S).**

9.0 Study Timelines

The duration of an individual subject's participation in the study and the duration anticipated to enroll all study subjects varies by activity. See section 5.0 for details.

The estimated date for the investigators to complete primary analyses is September 30, 2023. Secondary analyses are estimated to be completed by August 31, 2024. The estimated date of study completion is December 31, 2024. Dates are subject to change.

10.0 Study Endpoints and Statistical Analyses

The detailed protocol statistical analysis plan (SAP) can be found in Appendix TT.

Analysis of activity 1 (stakeholder engagement):

Quantitative interviews will be scored using their established scoring metrics:

- The NOMAD instrument is a 23-item survey that measures implementation processes from the perspective of professionals directly involved in the work of implementing complex interventions in healthcare. [Appendix J]
- The AIM (Acceptability of Intervention Measure), IAM (Intervention Appropriateness Measure), and FIM (Feasibility of Intervention Measure) instruments are four-item measures of implementation outcomes that are often considered "leading indicators" of implementation success. [Appendix G]
- The CSAT (Clinical Sustainability Assessment Tool) instrument is a 49-item survey with 7 domains used be evaluators and researchers to determine the extent to which a practice is being implemented effectively. (Appendix Y)
- The ORCA (Organizational Readiness to Change Assessment) instrument is a 74-item survey with three domains, each item has been mapped to a CFIR construct, and will be scored according to Helfrich et al.'s (2009). [Appendix B]
- The OCM (Organizational Change Manager) instrument is a 60-item survey with 15 domains, each item has been mapped to a CFIR construct, and will be scored according to Gustafson et al.'s (2003). [Appendix B2]

Qualitative interviews (audio recordings and notes) will be evalutated for common themes. Results from the qualitative and quantitiative interviews and surveys will be used to inform eSyM, its content, its rollout, and the training materials.

Analysis of Activity 3 (UAT/Pilot study):
Study staff will observe patient/proxy, clinician and clinical support staff action steps and interactions with the system. Observations will be recorded and used to refine eSyM, its rollout, and the training materials.

Any patient data collected will be analyzed as per the analysis plan for the stepped-wedge cluster randomized trial described below.

Analysis of Activity 4 (stepped-wedge trial):

Additional details can be found in Appendix TT. Please reference Appendix TT for the most updated SAP.

Study Design: Type II Hybrid Effectiveness-Implementation Stepped Wedge Cluster Randomized Trial. The research team will partner with software developers at Epic to adapt working ePRO symptom management systems, one in surgical and one in medical oncology, and fully integrate them into the EHR at 6 health systems. After pilot testing, we will conduct a pragmatic stepped wedge cluster randomized trial to measure the effectiveness of the ePRO system on outcomes that matter to patients and clinicians. Throughout, we will evaluate the implementation process to optimize sustainability and generate actionable knowledge that facilitates scaling to other settings. The proposed study is a hybrid effectiveness-implementation type I trial according to the Curran schema.[131] The study meets PRECIS-2 criteria for pragmatic trials based on scores of 4 or higher in each domain.[132] Reports will adhere to the revised Criteria for Reporting the Development and Evaluation of Complex Interventions in healthcare (CReDECI2),[133] the CONSORT PRO[134] and cluster randomized extensions,[135] and the stepped wedge reporting guidelines proposed by Grayling.[136]

**The PICOTS FRAMEWORK.**[63]	
Populations	1) Adult patients with GI, GYN, or Thoracic cancer having a new treatment plan<br>2) Adult patients with suspected GI, GYN, or thoracic cancer having a priority surgery<br><br>***Patients were seen at the participating sites between January 1, 2018 and March 31, 2024. Dates are subject to change.***
Intervention	**eSyM**: A multi-component **e**PRO **Sy**mptom **M**anagement system based on **PRO-CTCAE** with:<br>1. Patient-facing components:<br>• Prompts for between-clinic visit symptom reporting and the ability to view profiles over time<br>• Evidenced-based education for self-management in response to symptom reports<br>• Alerts to contact the clinical team in response to severe or escalating symptoms

	2. <u>Clinician-facing components:</u><br>• View patient profiles in EHR flowsheet, accessible between or during visits, easily added to notes<br>• Receive alerts for severe symptoms in the EHR, same as for abnormal lab tests<br>• Dashboard functionality to track symptoms for groups of patients
Control	No eSyM symptom reporting
Outcomes	Emergency department visits culminating in discharge, hospitalizations, symptom burden, care satisfaction
Timing	4-year study of surgical and medical oncology patients each followed for up to 1 year after the trigger event (i.e. new chemotherapy treatment plan and/or surgery)
Setting	Six health care systems, all small cancer centers. All use an Epic EHR.
Design	A hybrid Type II effectiveness-implementation pragmatic cluster randomized trial, with stepped wedge rollout (6 steps), patient as the unit of analysis and closed (cross-sectional) cohort design

Randomization:

We will conduct a stepped wedge cluster randomized trial to determine whether ePROs are clinically effective. This stepped wedge design has 6 steps, and we randomize 6 sites (Table) to the steps.

Table: Six participating sites and region

Site	Region
WVU	Southern
Baptist	
Dartmouth	Northern
Maine	
DFCI/BWH/MGH	Urban
Lifespan	

To ensure that two sites with the same attribution will not be assigned to the same rollout step group (early MO/late surgery or late MO/early surgery), we employ a stratified randomization by region. As a result, each of the two groups has a site from each of the three regions (Northern, Southern, and Metropolitan). The stepped wedge design includes seven time-periods, including a run-in period (Figure). .

Figure: Study design

Group	Step	Run-in	Period 1	Period 2	Period 3	Period 4	Period 5	Period 6
Early MO /late surgery group	Step 1		MO	MO	MO	MO	MO	MO
								Surgery
	Step 2			MO	MO	MO	MO	MO
							Surgery	Surgery

	Step 3				MO	MO	MO	MO
						Surgery	Surgery	Surgery
Late MO /early surgery group	Step 4					MO	MO	MO
					Surgery	Surgery	Surgery	Surgery
	Step 5						MO	MO
				Surgery	Surgery	Surgery	Surgery	Surgery
	Step 6							MO
			Surgery	Surgery	Surgery	Surgery	Surgery	Surgery

MO: Medical oncology
Surgery: Surgical patients

<u>The sites were randomized on 11/6/18 as follows:</u>

Group	Step	Run-in	Period 1	Period 2	Period 3	Period 4	Period 5	Period 6
Early MO /late surgery group	Baptist		MO	MO	MO	MO	MO	MO
								Surgery
	Maine			MO	MO	MO	MO	MO
							Surgery	Surgery
	Dana-Farber/BWH				MO	MO	MO	MO
						Surgery	Surgery	Surgery
Late MO /early surgery group	Lifespan					MO	MO	MO
					Surgery	Surgery	Surgery	Surgery
	Dartmouth						MO	MO
				Surgery	Surgery	Surgery	Surgery	Surgery
	WVU							MO
			Surgery	Surgery	Surgery	Surgery	Surgery	Surgery

<u>Analysis of Activity 4d (Patient qualitative interviews):</u>
Qualitative interviews (audio recordings and notes) will be evalutated for common themes using NVIVO. Results from the qualitative interviews will be used to inform eSyM enhancements, program content, program rollout, and implementation strategies.

<u>Analysis of Activity 5:</u>

We will extract structured data from the six health systems participating in SIMPRO. These data include ePROs, demographics, diagnoses, treatment plans, medications, and inpatient and outpatient encounters. We will develop risk prediction models for adverse events, especially including, but not limited to hospitalizations and emergency department visits. Planned hospitalizations will be identified and excluded based on A) admissions within 24 hours of the

triggering surgery among surgical patients; and B) admissions associated with curative-intent surgeries among medical oncology patients with early-stage cancers. For models incorporating ePROs as predictors, the analytic cohort will include patients who completed at least one survey. For models excluding ePROs as predictors, the analytic cohort will include all patients in the database.

Data from at least one health system will be removed from the dataset to serve as an external test set. Among the remaining health systems, the population will be split at the patient level with 70% randomly assigned to a training cohort, 15% assigned a validation cohort and 15% assigned to an internal test set. To account for differences in clinical practice and demographics across health systems, randomization will be stratified by system. The same cohorts will be used to train and test all algorithms.

To determine the optimal machine learning model, we will evaluate multiple types of architectures, including logistic regression, random forests, gradient boosted machines, extreme gradient boosting, neural networks, and ensemble models. Hyperparameters will be tuned using the validation cohort and models iteratively trained until a final candidate model is identified. This model will then be evaluated on the held-out internal test set and the external test cohort, after which no further model training will be performed. These steps will be performed separately for the ePRO-responding cohort and the total cohort.

To develop clinical risk scores, we will select approximately 20 independent variables that can be easily abstracted from a patient's medical chart, such as diagnosis, treatment, comorbid conditions, recent hospitalizations, and demographics. Within the training cohort, we plan to use logistic regression with L1 regularization to select variables for inclusion and define the prediction model. The amount of regularization will be tuned using the validation cohort and models will be iteratively trained until a final candidate model is identified. Like above, these steps will be completed separately for the ePRO-responding and total cohorts. The area under the receiver operator characteristic curve (AUC-ROC) will be used to compare the performance (i.e., predictive ability) of different models.

11.0 Procedures Involved

The study design, study procedures and safety monitoring vary by activity. See section 5.0 for details.

12.0 Data Management and Confidentiality

DF/HCC uses a clinical trial management system (CTMS) called OnCore, which is managed by the Office of Data Quality (ODQ).

- **Activity 1** is research exempt from IRB review and involves engaging stakeholders. This will be achieved via emailed surveys or in group settings using discussion and handheld polling devices. We will collect basic demographic information. We will collect age group, gender, race, and ethnicity. We will NOT collect each participant's initials and date of birth. We will enter summary/batch accrual

information into Dana-Farber's CTMS OnCore for Activity 1. Individual registration is not feasible as OnCore mandates DOB.

- **Activity 2** is NHSR; registration in OnCore is not applicable.
- **Activities 3 and 4** will be largely automated. Epic will be programmed to identify and/or push eSyM out to applicable patient recipients. This is an implementation/QI study, not a traditional research study, so the consent/registration/intervention paradigm does not fit activities 3, 4a, 4b, and 4c. Activity 4d will include patient consent. Furthermore, activity 4 will accrue a *minimum* of 6048 participants. For these reasons, manual registration of each participant into OnCore before exposure to eSyM and/or the SASS questionnaire is not possible. Initials, date of birth, gender, race and ethnicity will be collected, and we will work with ODQ to provide ODQ with this data for all participants from all sites so that it can be imported into OnCore.
- **Activity 5** will involve a retrospective review of data collected in Activity 4. Registration in OnCore is not applicable.

Data security: PHI data will be collected using multiple applications: REDCap, eSyM/MyChart, Epic.

REDCap: For this study, data will be collected using the Partners instance of REDCap (redcap.partners.org). Consent for Activity 4D will also be collected using REDCap. In collaboration with the Harvard Catalyst | The Harvard Clinical and Translational Science Center, REDCap (Research Electronic Data Capture) is a free, secure, HIPAA compliant web-based application hosted by Partners HealthCare Research Computing, Enterprise Research Infrastructure & Services (ERIS). Vanderbilt University, with collaboration from a consortium of academic and non-profit institutional partners, develops this software application for electronic collection and management of research and clinical study data. Data collection is customized for each study or clinical trial by the research team with guidance from ERIS REDCap administrators. REDCap is built around HIPAA guidelines and is 21 CFR Part 11 capable.

eSyM (which will be built into Epic): Epic applications employ a variety of technical safeguards to protect the confidentiality, integrity, and availability of personal information including supporting Transport Layer Security (TLS)/Secure Sockets Layer (SSL) certificate technology and encryption.

To maximize data security, both REDCap and eSyM/Epic employ:

User Privileges. To ensure that users have access only to data and information that they are supposed to have within the application, user privileges are utilized within the software. Each user has their own account, and their user account will only have access to information that they themselves have created or to which administrators have granted them access.

Password-protection & Authentication. Both systems are password protected and implement authentication to validate the identity of end-users that log in to the system.

Auto-logout setting will automatically log a user out of the system if they have not had any activity (e.g., typing, moving the mouse) on their current web page for the set amount of time. This prevents someone else from accessing their account and their data if they leave a workstation without properly logging out or closing their browser window.

Logging and Audit Trail. Both systems maintain built-in audit trails that log all user activity and all pages viewed by every user.

Study specific procedure to maximize data security:

Controlled access: The REDCap administrator at the coordinating site (Dana-Farber) will set up all user accounts so that each user only has access to their own site's data.

Use of unique study ID numbers: REDCap automatically assigns unique study ID numbers to each new case.

Extensive training: All personnel involved in this study are required to complete and document completion of extensive protocol training. Furthermore, all project investigators and staff are required to have valid certification of human subjects' research training.

Quality control: The staff at the coordinating center (Dana-Farber) will be responsible for monitoring the data for completion, accuracy, and compliance.

SFA storage: All study files will be maintained on a MGB HIPPA-compliant SFA. Backup copies will also be saved on an encrypted external hard drive. The study PI and PM will oversee user privileges to each of these storage areas and perform regular user audits to ensure proper access is maintained to all study files.

Data collection and submission processes:

At each local site: Hard copies of applicable study documents (i.e., printed SASS questionnaires, qualitative interview consent forms, etc.) will be kept in local study files in locked cabinets. Local copies of study materials may be destroyed 5-years after the primary completion date or at the discretion of the local site PI and local site IRB. NOTE: Prior to conducting any patient qualitative interviews, the coordinating center will request that a copy of the signed patient ICF be transmitted to DFCI. See below for data transmission methods. As appropriate, electronic ICF may also be obtained using REDCap.

Data submission to the central coordinating site: Local sites will transmit data to the central coordinating site using multiple methods: (1) study data will be submitted via REDCap; for this study, we will use the Partners Healthcare instance of REDCap available here: https://redcap.partners.org/redcap/. Sites will log into REDCap, fill out CRFs and upload source documentation and other files, and submit. (2) data downloads from eSyM/Epic will be transmitted to the central site using secure file transfer protocol (FTP). We will use a secure FTP managed by the Epic EHR vendor or we will use Partner's Healthcare secure FTP available here: https://transfer.partners.org/courier/web/1000@/wmLogin.html or the Partners automated Diplomat SFTP platform. (3) When appropriate, sites may transmit data via email. When appropriate, the email can be encrypted by typing "Send Secure" in the email subject line. (4) Epic may share site-specific data reports with the coordinating center (5) Due to the size and complexity of this project, other methods may be used if they are preapproved by the Dana-Farber PI and IT.

Please note: All sites will transmit a limited data set to the coordinating center in accordance with the SIMPRO-developed Data Use Agreement, which has been agreed upon by all participating sites.

Summary of data collection:

**Data Sources & Representative Data Elements**						
**Epic Data**			**Patient survey**	**Collection/ abstraction**	**Derived data**	**Patient interview**
**Entered by the patient**	**Entered by a clinician**	**Recorded processes**				
PROs, medical history	Medical problems; chemo regimens	Hospital admissions; proportion of surveys completed	Quality of life; symptoms, self-efficacy	Cancer stage; disease status<br><br>Provider/hospital type & volume	Number of urgent clinic visits for symptom management; Comorbidity	Feedback on eSyM experiences (to be recorded and transcribed)

**Data Levels, Descriptions & Representative Data Elements**			
**Patient-encounter**	**Patient**	**Population**	**Healthcare system**
Patient features that could change/recur over time	Patient features that do not change over time	Feature of multiple patients	Feature of a site or provider
PROs<br>Disease status<br>Urgent clinic encounters<br>ED encounters<br>Hospitalizations<br>Chemotherapy plans<br>Health literacy	Date of birth/age<br>Race/ethnicity<br>Cancer type<br>Cancer diagnosis date<br>Surgery type and date	Proportion of patients reporting a serious (i.e., high grade) symptom<br>Proportion of patients responding to a survey	Urban/rural setting<br>Medical/surgical provider<br>High/low volume facility<br>Teaching hospital status

<u>Data collection from EPIC:</u>

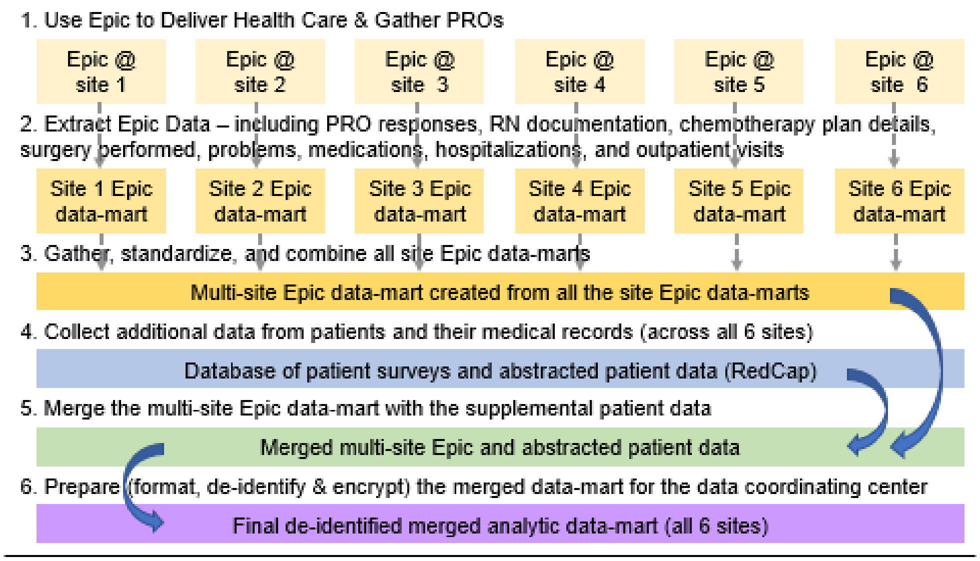

<u>Summary of Data Sharing:</u>

Each Data Provider, including DFCI/BWH/MGH, will provide the following DATA SET to DFCI on a quarterly basis:

<u>Activity 1 (Stakeholder Feedback):</u> Stakeholders include physicians, nurses, allied health care professionals, practice administrators, information technology support staff and administrators

- Email addresses for stakeholder participants (for REDCap administration ONLY)
- Basic demographic information from stakeholders (including # of years working for their organization, professional job category, gender, racial background, ethnicity, age group) collected via Clicker questions, REDCap, and/or email
- Audio recordings from UM1 focus groups and/or individual qualitative interviews
- Transcripts from UM1 focus groups and/or qualitative interviews
    - Meeting minutes from UM1 focus groups. Feedback may be collected in an identifiable fashion. For example, the CIO's comments may be attributed to "the CIO."
- Clicker-question responses from UM1 focus groups (i.e., NOMAD, CFIR, AIM items etc.)
- REDCap survey responses (i.e., NOMAD, CFIR, AIM Items, etc.)

<u>Activity 2 (Build & deploy eSyM)</u>

- NO data transfer required

Activity 3a (eSyM App Usage)

- Participant responses to the symptom reporting questions within eSyM (collected via Epic/MyChart)
- Participant MyChart activity including login dates, mode of usage, and number of messages sent to the care team
- Clinician responses to symptom reports (collected via Epic) including # of documented eSyM encounters and InBasket message responses
- Data on eSyM usage by all user types (collected via Epic/MyChart)

Activity 3b (eSyM User Acceptability Testing)

- Notes and observations taken during user acceptability testing

***No PHI will be collected during UAT*

Activity 3c (Medical Record Abstraction): This activity is necessary to understand how exposure to eSyM influences rates of acute care visits.

- Patient identifier - study ID # which may be an encrypted MRN (collected via Epic and encrypted as needed by site study team). Study teams at each site will encrypt actual MRNs and provide a study ID#. Suggested algorithms and code will be provided but this must be done at each site. Each site must separately store the "master key" that links real MRN to study ID#. Actual MRNs will not be released to the DFCI or shared with the NCI.
- Patient study site – identifying which hospital system and which clinic site a patient is seen at for those hospitals having more than one site.
- Patient zip code of residence (collected via Epic)
- Patient DOB – This may be encrypted. The year of birth is required. However, sites can elect to encrypt birth date and month. For example, a site may encrypt through an algorithm that: a) adds 4 months and 11 days to all birth dates that are in even number calendar years and subtracts 3 months and 16 days from all birth dates that are in odd number years. Each site is responsible for developing and deploying its own encryption file and storing it locally in secure fashion.
- Patient vital status
- Patient demographic information, including sex, race, ethnicity, language, marital status, employment status, and education level
- Patient eSyM inclusion information, including cancer diagnosis data and/or procedure data
    - Cancer diagnosis code (ICD-10 codes), date and description
    - Complete Epic problem list
    - Procedure code (CPT code), date, description, and goal
    - Cancer staging code
    - Treatment plan information, including start date, end date, intention
    - Medications list
- Patient dates of service including surgery, chemo administration, follow-up appointments, etc. (collected via Epic)
- Study-outcomes collected via Epic including:
    - Hospital readmission rates within 90 days for surgical patients

        - Information will include visit dates, discharge diagnosis codes
    - Hospital admission rates within 90 days of chemotherapy for medical oncology patients.
        - Information will include visit dates, discharge diagnosis codes
    - Emergency department visits (including those that culminate in admission versus those that culminate in discharge) for surgical and medical oncology patients
        - Information will include visit dates, discharge diagnosis codes
    - Dates of chemotherapy administration

Please note – Additional medical record data and outcomes will be collected via Epic as determined by the SIMPRO consortium. All requested items will still fall within the parameters of a limited data set

<u>Activity 4a (eSyM App Usage)</u>

- Participant MyChart activity including login dates, mode of usage, and number of messages sent to the care team
- Data on eSyM usage by all user types
- Clinician responses to symptom reports (collected via Epic) including # of documented eSyM encounters and InBasket message responses

<u>Activity 4b (SASS Questionnaire)</u>

- Patient contact information (email address and/or postal address) to be inputted into Partners REDCap for survey administration ONLY. There is no other use of patient emails as part of this study.
- Patient-reported self-efficacy, attainment of information needs, symptom burden, and satisfaction with care using validated metrics.

<u>Activity 4c (Medical Record Abstraction)</u>

- Patient identifier for all patients in the denominator cohort (eSyM users and non-users) - study ID # and/or encrypted MRN (collected via Epic and encrypted as needed by site study team)
- Patient study site ID for all patients in the denominator cohort (eSyM users and non-users)
- Patient zip code of residence (collected from Epic registration field)
- Patient DOB for all patients in the denominator cohort (eSyM users and non-users) – DOB can have encrypted birth month and day as described above under activity 3c.
- Patient vital status
- Patient demographic information, including sex, race, ethnicity, language, marital status, employment status, and education level
- Patient eSyM inclusion information, including cancer diagnosis data and/or procedure data
    - Cancer diagnosis code (ICD-10 codes), date and description
    - Complete Epic problem list
    - Procedure code (CPT code), date, description, and goal
    - Cancer staging code

  - Treatment plan information, including start date, end date, intention
  - Medications list
- Patient dates of service including surgery, chemo administration, follow-up appointments (collected via Epic) for all patients in the denominator cohort (eSyM users and non-users) Please see detail under Activity 3c.
- Study-outcomes collected via Epic including:
  - Hospital readmission rates within 90 days for surgical patients
    - Information will include visit dates, discharge diagnosis codes
  - Hospital admission rates within 90 days of chemotherapy for medical oncology patients.
    - Information will include visit dates, discharge diagnosis codes
  - Emergency department visits (including those that culminate in admission versus those that culminate in discharge) for surgical and medical oncology patients
    - Information will include visit dates, discharge diagnosis codes
  - Dates of chemotherapy administration

<u>Activity 4d (Patient Interviews)**</u>

- Name, phone number, and email addresses for consented patient participants (for interview scheduling purposes)
- Basic demographic and diagnosis information as well as eSyM history will be collected directly from patients
- Audio recordings from qualitative interviews
- Transcripts from qualitative interviews
  - Transcripts will be deidentified (e.g. names, treatment location, state, etc)

**NOTE: As Activity 4d is a consented protocol activity, identifiable information will be shared with the coordinating center. During the consenting process, enrolling participants will be made aware and attest through their written consent that their contact information will be shared with the coordinating center and that interviews will be conducted directly by the coordinating center, not the local study team. Participants will have the option to opt out at any time. After consent and upon completion of the qualitative interviews, audio recordings will be transcribed and interview transcripts will be deidentified prior to analyses, publications, or future data sharing activities

Please note – Additional medical record data and outcomes will be collected via Epic as determined by the SIMPRO consortium. All requested items will still fall within the parameters of a limited data set.

***Please note:** All data stored at the Dana Farber Cancer Institute (SIMPRO Coordinating Center) is kept under double password protected servers that routinely have audits to examine access.*

Investigators at all SIMPRO Data Provider institutions will have access to the full compiled program limited data set (e.g., all data explicitly listed above as well as any other data

collected and shared through this limited data set agreement). In addition, this complete data set will be shared with the designated study chair and team at Memorial Sloan Kettering Cancer Center.

DFCI will provide the following limited dataset to the National Cancer Institute (IMS):

- Stakeholder Data
  - Aggregate site participation #'s
  - Hospital and provider characteristics (as available)
  - Transcripts from UM1 focus groups and/or qualitative interviews
    - Meeting minutes from UM1 focus groups. Feedback may be collected in an identifiable fashion. For example, the CIO's comments may be attributed to "the CIO."
  - Stakeholder clicker-question responses from UM1 focus groups (i.e., NOMAD, CFIR, AIM items etc.)
  - Stakeholder REDCap survey responses (i.e., NOMAD, CFIR, AIM Items, etc.)
- Patient Data
  - Study ID for SASS survey respondents
  - Responses for each survey respondent
  - Patient demographics
    - Patient zip code
    - Age (interval linked to anchor date)
    - Sex, race, ethnicity, language, marital status, employment status, state of residence and education level
  - Enrollment
    - Procedure type and category
    - Tx plan and type
  - Clinical encounters and appointments
    - Encrypted date of procedure
    - Interval between surgery and hospital discharge
    - Interval between hospital discharge and eSyM symptom reports
    - Interval between hospital discharge and ED visits
    - Interval between hospital discharge and rehospitalizations
    - Encrypted date of new chemo and treatment administrations
    - Interval between new chemo and eSyM reports
    - Interval between new chemo and ED visits
    - Interval between new chemo and rehospitalizations
    - Hospital readmission and ED rates within 30 and 90 days of chemo/surgery
  - Cancer diagnoses
  - Comorbid condition diagnoses
  - Procedures performed during encounters
    - Laboratory data with encrypted date fields (i.e., intervals based on anchor date)
  - Cancer-directed medication administrations and/or orders

        - Medication list with encrypted date fields (i.e., intervals based on anchor date)
    - Symptom-directed medication administrations and/or orders
    - Vital signs data (body mass index (BMI) measurements only)

Please note – Additional medical record data and outcomes will be collected via Epic as determined by the SIMPRO consortium and may be shared with the NCI. All requested items will still fall within the parameters of a limited data set

Please note:

- Deidentified, patient level data will be compiled from the SIMPRO consortium (*Dana-Farber Cancer Institute, Maine Health, Dartmouth-Hitchcock Memorial Hospital, Baptist Memorial Health Care Corporation, Lifespan Cancer Institute/Rhode Island Hospital, West Virginia University Hospitals, Inc.)*
- The PI and project manager of each of the 6 SIMPRO sites will review all data to be shared with the NCI.

13.0 Provisions to Monitor the Data to Ensure the Safety of Subjects

Please see the Data Safety and Monitoring Plan (Appendix S).

14.0 Future Use of Data

This study does not involve any specimen collection/banking of any kind.
Personal health information will be collected as part of this study.

All data collected during this study will be stored and used for future research. Any personal identifiers will be removed so that the information cannot be linked back to a patient. Details on data use cases can be found below:

1) **Local site investigators utilizing SIMPRO data collected at their home institution (one site) –** Investigators should receive local site PI approval to obtain and analyze internal site data. IRB approval should be obtained locally as needed.
2) **SIMPRO investigators utilizing SIMPRO data –** All analyses are covered through this protocol. No additional approvals are needed.
3) **Investigators at SIMPRO sites utilizing SIMPRO data outside of the scope of the original grant -** Investigators, including those from collaborating institutions, can request the data collected from this study for new research. Requests must be sent to the Coordinating Center Site PI (Michael Hassett MD, MPH). All data requests and analysis plans must be approved by the coordinating center sIRB (WIRB) and local site IRBs, as required, through this protocol or stand-alone protocols.
4) **NCI and IMPACT collaborators utilizing SIMPRO data -** Per the SIMPRO-developed data use agreement, a limited data set will be shared with the National

Institutes of Health (National Cancer Institute) and IMPACT consortium member sites in accordance with funding requirements. Publications and presentations resulting from analyses using SIMPRO data will include appropriate credits and authorship as agreed to with SIMPRO investigators.

5) **External collaborators utilizing SIMPRO data** – Data may also be shared with outside non-profit academic investigators as well as with for-profit pharmaceutical investigators or commercial entities with whom Dana-Farber collaborates. Requests must be sent to the Coordinating Center Site PI (Michael Hassett MD, MPH). All data requests and analysis plans must be approved by the coordinating center and appropriate IRB approvals and data transfer agreements will be obtained.

The study letters inform the participant that data collected for this study may be used in the future. Participants will not be asked to provide additional informed consent for the use of de-identified information in future research.

There is no scheduled date on which the information and data that is being used or shared for this research will be destroyed, because research is an ongoing process. The eventual goal is to make deidentified SIMPRO data publicly available through collaborations with the NCI.

15.0 Withdrawal of Subjects

Symptom reporting via eSyM per protocol will continue for 60-180-days; after this period, the local site may decide whether to continue having the patient report symptoms via eSyM or not. Observational follow-up will continue for 1-year. If a patient submits a request in writing to be removed from eSyM, then their local site will deactivate their account. Any data collected up to that point will be kept and included in analyses. Patient status [denominator group, eSyM active in 60-90-day window, eSyM active beyond 60-90-day window, written request of withdrawal, 1-year observational window, or post-1-year observational window] will be captured via eSyM and REDCap CRF.

16.0 Risks to Subjects

There are risks to taking part in any research study. The primary risk of this study is loss of privacy or confidentiality. The risk of loss of privacy or confidentiality by using eSyM or taking part in this study is minimal. The study team has taken many steps to prevent any loss of privacy or confidentiality, including training of all clinic and research staff in best practices, rules, and regulations surrounding privacy and confidentiality, collecting research data using unique study ID numbers instead of names or other identifying information, and use of data collection systems that meet the NIH's data security standards.

17.0 Potential Benefits to Subjects

Using eSyM may or may not benefit participants. We hope that by using eSyM, patients are able to better manage their symptoms and the increased flow of information between a patient and their care team improves their experiences. We also hope the information learned from this research study will provide more information about how to best help patients, caregivers, and their care team work together during and between visits to achieve better symptom management in cancer patients.

18.0 Vulnerable Populations

This protocol does not involve vulnerable populations of prisoners and children. Prisoners and children are excluded.

Cognitively impaired adults may participate because the risk is negligible. This protocol only involves questionnaires, opinion surveys and symptom reporting via an internet enabled app (MyChart). Additionally, cognitive status is not reliably captured in the EHR and, as this project involves automated identification of patients, we will not be able to exclude patients prior to eSyM assignment based on cognitive status.

Pregnant women may participate because the risk to the woman and her fetus is negligible. This protocol only involves questionnaires, opinion surveys and symptom reporting via an internet enabled app (MyChart).

19.0 Sharing of Results with Subjects

Participants will be directed to clinicaltrials.gov for research study results.

20.0 Setting

This is a multi-site study that will take place at 6 sites in the US. Dana-Farber will serve as the coordinating site.

21.0 Resources Available

The feasibility of this study is based on estimates obtained from the 2016 analytic cases of the tumor registrar at each of the participating sites. Although there is some heterogeneity based on tumor type, there are more than adequate new cancer cases at each participating site to meet the study minimum accrual goals of 144 surgical and 144 medical oncology patients per year. The entire statistical analysis section is predicated on minimum accrual targets. Because the goal is

broad-based systems-level implementation, we anticipate having much higher accrual which will enable us to examine effectiveness in subgroups of interest. Additional patient accrual adds relatively little to the cost of executing the study given that we propose a limited number of surveys and limited manual record review. Ascertaining potential subjects, their outcomes and relevant covariates can be done through Epic. The hard work is implementation and changing clinical workflow. Once successfully implemented, we project that the incremental work of engaging more patients in eSyM will be minimal.

22.0 Provisions to Protect the Privacy Interests of Subjects

Please see sections 11 and 12 for details.

23.0 Compensation for Research-Related Injury

There is no compensation in the event of research related injury.

24.0 Economic Burden to Subjects

Costs that subjects may be responsible for because of participation in the research: Subjects participating in this study will be asked to complete online surveys and/or report their symptoms from home using an internet-enabled device. Participants will have to use their own hardware (e.g., smartphone, tablet, computer) and their own Wi-Fi or Data Plan which may cost them money; subjects are responsible for these costs. Devices and/or data plans will not be provided by the study.

Patients who participate in the SASS questionnaire and/or patient qualitative interviews will each be offered a $15.00 gift card as a thank you.

25.0 Consent Process

Each activity utilizes different consent methods. See section 5.0 for details.

26.0 Appendices

***All appendices may be branded with site-specific logos as needed; wherever applicable, the Dana-Farber Cancer Institute logo may be removed and replaced with the applicable site logo. In addition, all materials will regularly be reviewed and updated as eSyM content is revised*

Question Banks:
Appendix A: CFIR Qualitative Question Bank
Appendix B: ORCA Quantitative Question Bank
Appendix B2: OCM Quantitative Question Bank
Appendix B3: eHIQ Quantitative Interview Question Bank
Appendix C: CAHPS Question Bank for cancer surgery
Appendix D: CAHPS Question Bank for cancer drugs
Appendix E: CAHPS Question Bank supplemental questions
Appendix F: PROMIS Question Bank
Appendix G: AIM/IAM Question Bank
Appendix H: PROCTCAE Question Bank – English
Appendix I: PROCTCAE Question Bank – Spanish
Appendix J: NOMAD Question Bank

Recruitment/Consent Materials:
Appendix K: Stakeholder recruitment email 1 (informing development)
Appendix L: Stakeholder recruitment email 2 (evaluating implementation)
Appendix M: Patient Invitation to participate in research questionnaire
Appendix N: Patient Invitation to use eSyM
Appendix O: Model Study Letter for Activity 3b (UAT) (waiver of documentation of consent)
Appendix P: UAT Observation Guide
Appendix Q: eSyM Disclaimer (NHSR)
Appendix R: Model Study Letter for Activity 4b (SASS Questionnaire) (waiver of documentation of consent)
Appendix S: DSMP

Study References:
Appendix T1: eSyM Medical Oncology Patient-Facing Tip Sheets
Appendix T2: eSyM Surgical Patient-Facing Tip Sheets
Appendix U1: Patient-Facing SASS Questionnaire – MO eSyM+ Version
Appendix U2: Patient-Facing SASS Questionnaire – MO eSyM+ Non-Responder Version
Appendix V: Patient-Facing SASS Questionnaire – MO eSyM- Version
Appendix W1: Patient-Facing SASS Questionnaire – SO eSyM+ Version
Appendix W2 : Patient-Facing SASS Questionnaire – SO eSyM+ Non-Responder Version
Appendix X: Patient-Facing SASS Questionnaire – SO eSyM- Version
Appendix Y: Stakeholder Interview Guide
Appendix Z: Patient-Facing SASS Questionnaire Cover Letter

eSyM Training & Marketing Materials:
Appendix AA: eSyM Clinician User Guide
Appendix BB: Clinician-Facing eSyM Flyer
Appendix CC: Clinician-Facing FAQs Page
Appendix DD: Patient-Facing eSyM Flyer
Appendix EE: Patient-Facing eSyM Pamphlet
Appendix FF: Patient-Facing eSyM User Guide
Appendix GG: Patient-Facing FAQs Page
Appendix HH: Patient-Facing FAQs Page for eSyM Home Page
Appendix II: Site Staff eSyM User Guide
Appendix JJ: Patient eSyM Promo Video
Appendix KK: Provider eSyM Promo Video
Appendix LL: Project Website
Appendix MM: Other Resources Medical Oncology
Appendix NN: Other Resources Surgical
Appendix OO: Patient-Facing eSyM Additional Questions (Activity Level & Overall Wellbeing)
Appendix PP: Training Flipbooks (Patient & Staff-Facing)

Additional Appendices:
Appendix QQ: Model Informed Consent Form for Activity 4d
Appendix RR: Qualitative Patient Interview Guide
Appendix SS: Qualitative Patient Interview Intro Letter
Appendix TT: Statistical Analysis Plan (SAP)

27.0 References

1. Cancer Statistics. National Cancer Institute. https://www.cancer.gov/about-cancer/understanding/statistics. Published 2017. Accessed Accessed on 1/2/18.
2. Cleeland CS. Symptom burden: multiple symptoms and their impact as patient-reported outcomes. *Journal of the National Cancer Institute Monographs.* 2007(37):16-21.
3. Hofman M, Ryan JL, Figueroa-Moseley CD, Jean-Pierre P, Morrow GR. Cancer-related fatigue: the scale of the problem. *Oncologist.* 2007;12 Suppl 1:4-10.
4. Teunissen SC, Wesker W, Kruitwagen C, de Haes HC, Voest EE, de Graeff A. Symptom prevalence in patients with incurable cancer: a systematic review. *Journal of pain and symptom management.* 2007;34(1):94-104.
5. Temel JS, Pirl WF, Lynch TJ. Comprehensive symptom management in patients with advanced-stage non-small-cell lung cancer. *Clin Lung Cancer.* 2006;7(4):241-249.
6. Mayer DK, Travers D, Wyss A, Leak A, Waller A. Why do patients with cancer visit emergency departments? Results of a 2008 population study in North Carolina. *Journal of clinical oncology : official journal of the American Society of Clinical Oncology.* 2011;29(19):2683-2688.
7. Brooks GA, Abrams TA, Meyerhardt JA, et al. Identification of potentially avoidable hospitalizations in patients with GI cancer. *Journal of clinical oncology : official journal of the American Society of Clinical Oncology.* 2014;32(6):496-503.
8. Barbera LT, C; Dudgeon, D. Why do patients with cancer visit the emergency department near the end of life? *CMAJ.* 2010.
9. Berry DL, Blonquist TM, Hong F, Halpenny B, Partridge AH. Self-reported adherence to oral cancer therapy: relationships with symptom distress, depression, and personal characteristics. *Patient Prefer Adherence.* 2015;9:1587-1592.
10. Fish JA, Prichard I, Ettridge K, Grunfeld EA, Wilson C. Psychosocial factors that influence men's help-seeking for cancer symptoms: a systematic synthesis of mixed methods research. *Psycho-oncology.* 2015.
11. Nathan H, Yin H, Wong SL. Postoperative Complications and Long-Term Survival After Complex Cancer Resection. *Annals of surgical oncology.* 2017;24(3):638-644.
12. Hendren S, Birkmeyer JD, Yin H, Banerjee M, Sonnenday C, Morris AM. Surgical complications are associated with omission of chemotherapy for stage III colorectal cancer. *Dis Colon Rectum.* 2010;53(12):1587-1593.
13. Tevis SE, Kohlnhofer BM, Stringfield S, et al. Postoperative complications in patients with rectal cancer are associated with delays in chemotherapy that lead to worse disease-free and overall survival. *Dis Colon Rectum.* 2013;56(12):1339-1348.
14. Anderson KO, Richman SP, Hurley J, et al. Cancer pain management among underserved minority outpatients: perceived needs and barriers to optimal control. *Cancer.* 2002;94(8):2295-2304.
15. Kwon JH. Overcoming barriers in cancer pain management. *Journal of clinical oncology : official journal of the American Society of Clinical Oncology.* 2014;32(16):1727-1733.

16. Cohen E, Botti M. Cancer Patients' Perceptions of the Barriers and Facilitators to Patient Participation in Symptom Management During an Episode of Admission. *Cancer Nurs.* 2015;38(6):458-465.
17. Bainbridge D, Seow H, Sussman J, et al. Multidisciplinary health care professionals' perceptions of the use and utility of a symptom assessment system for oncology patients. *Journal of oncology practice.* 2011;7(1):19-23.
18. Mobile Fact Sheet. Pew Research Center Internet and Technology. http://www.pewinternet.org/fact-sheet/mobile/. Published 2017. Accessed Accessed on 1/4/18.
19. Internet/Broadband Fact Sheet. Pew Research Center Internet and Technology. http://www.pewinternet.org/fact-sheet/internet-broadband/. Published 2017. Accessed Accessed on 1/4/18.
20. Birkhoff SD, Smeltzer SC. Perceptions of Smartphone User-Centered Mobile Health Tracking Apps Across Various Chronic Illness Populations: An Integrative Review. *J Nurs Scholarsh.* 2017;49(4):371-378.
21. Carroll JK, Moorhead A, Bond R, LeBlanc WG, Petrella RJ, Fiscella K. Who Uses Mobile Phone Health Apps and Does Use Matter? A Secondary Data Analytics Approach. *Journal of medical Internet research.* 2017;19(4):e125.
22. Ernsting C, Dombrowski SU, Oedekoven M, et al. Using Smartphones and Health Apps to Change and Manage Health Behaviors: A Population-Based Survey. *Journal of medical Internet research.* 2017;19(4):e101.
23. Koh HK, Brach C, Harris LM, Parchman ML. A proposed 'health literate care model' would constitute a systems approach to improving patients' engagement in care. *Health affairs (Project Hope).* 2013;32(2):357-367.
24. Carman KL, Dardess P, Maurer M, et al. Patient and family engagement: a framework for understanding the elements and developing interventions and policies. *Health affairs (Project Hope).* 2013;32(2):223-231.
25. Hibbard JH, Mahoney E. Toward a theory of patient and consumer activation. *Patient Educ Couns.* 2010;78(3):377-381.
26. Coulter A, Ellins J. Effectiveness of strategies for informing, educating, and involving patients. *BMJ (Clinical research ed).* 2007;335(7609):24-27.
27. Bandura A. Self-efficacy mechanism in human agency. *American Psychological Association.* 1982; 37(2), 122-147.
28. Strecher VJ, DeVellis BM, Becker MH, Rosenstock IM. The role of self-efficacy in achieving health behavior change. *Health Educ Q.* 1986;13(1):73-92.
29. Coleman K, Austin BT, Brach C, Wagner EH. Evidence on the Chronic Care Model in the new millennium. *Health affairs (Project Hope).* 2009;28(1):75-85.
30. Survey Snapshot: Health Care Providers on the Problems of Patient Engagement Design. NEJM Catalyst. https://catalyst.nejm.org/problems-patient-engagement-design/. Published 2017. Accessed Accessed on 1/4/18.
31. Manary MP, Boulding W, Staelin R, Glickman SW. The patient experience and health outcomes. *The New England journal of medicine.* 2013;368(3):201-203.

32. McCabe C, McCann M, Brady AM. Computer and mobile technology interventions for self-management in chronic obstructive pulmonary disease. *Cochrane Database Syst Rev.* 2017;5:CD011425.
33. Posadzki P, Mastellos N, Ryan R, et al. Automated telephone communication systems for preventive healthcare and management of long-term conditions. *Cochrane Database Syst Rev.* 2016;12:CD009921.
34. Basch E, Deal AM, Kris MG, et al. Symptom Monitoring With Patient-Reported Outcomes During Routine Cancer Treatment: A Randomized Controlled Trial. *Journal of clinical oncology : official journal of the American Society of Clinical Oncology.* 2016;34(6):557-565.
35. Snyder CF, Blackford AL, Aaronson NK, et al. Can patient-reported outcome measures identify cancer patients' most bothersome issues? *J Clin Oncol.* 2011;29(9):1216-1220.
36. Abelson JS, Symer M, Peters A, Charlson M, Yeo H. Mobile health apps and recovery after surgery: What are patients willing to do? *Am J Surg.* 2017;214(4):616-622.
37. Basch E, Deal AM, Dueck AC, et al. Overall Survival Results of a Trial Assessing Patient-Reported Outcomes for Symptom Monitoring During Routine Cancer Treatment. *Jama.* 2017;318(2):197-198.
38. McCorkle R, Ercolano E, Lazenby M, et al. Self-management: Enabling and empowering patients living with cancer as a chronic illness. *CA: a cancer journal for clinicians.* 2011;61(1):50-62.
39. Fisch MJ, Chung AE, Accordino MK. Using Technology to Improve Cancer Care: Social Media, Wearables, and Electronic Health Records. *Am Soc Clin Oncol Educ Book.* 2016;35:200-208.
40. Mobasheri MH, Johnston M, Syed UM, King D, Darzi A. The uses of smartphones and tablet devices in surgery: A systematic review of the literature. *Surgery.* 2015;158(5):1352-1371.
41. Basch E, Iasonos A, McDonough T, et al. Patient versus clinician symptom reporting using the National Cancer Institute Common Terminology Criteria for Adverse Events: results of a questionnaire-based study. *The Lancet Oncology.* 2006;7(11):903-909.
42. Dueck AC, Mendoza TR, Mitchell SA, et al. Validity and Reliability of the US National Cancer Institute's Patient-Reported Outcomes Version of the Common Terminology Criteria for Adverse Events (PRO-CTCAE). *JAMA oncology.* 2015;1(8):1051-1059.
43. Basch E, Reeve BB, Mitchell SA, et al. Development of the National Cancer Institute's patient-reported outcomes version of the common terminology criteria for adverse events (PRO-CTCAE). *Journal of the National Cancer Institute.* 2014;106(9).
44. Basch E, Snyder C, McNiff K, et al. Patient-reported outcome performance measures in oncology. *Journal of oncology practice.* 2014;10(3):209-211.
45. Snyder CF. Using patient-reported outcomes in clinical practice: a promising approach? *Journal of clinical oncology : official journal of the American Society of Clinical Oncology.* 2014;32(11):1099-1100.
46. Basch E, Snyder C. Overcoming barriers to integrating patient-reported outcomes in clinical practice and electronic health records. *Annals of oncology : official journal of the European Society for Medical Oncology.* 2017;28(10):2332-2333.

47. Snyder CF, Herman JM, White SM, et al. When using patient-reported outcomes in clinical practice, the measure matters: a randomized controlled trial. *Journal of oncology practice.* 2014;10(5):e299-306.
48. Roess A. The Promise, Growth, and Reality of Mobile Health - Another Data-free Zone. *The New England journal of medicine.* 2017;377(21):2010-2011.
49. Jensen RE, Snyder CF. PRO-cision Medicine: Personalizing Patient Care Using Patient-Reported Outcomes. *Journal of clinical oncology : official journal of the American Society of Clinical Oncology.* 2016;34(6):527-529.
50. Hughes EF, Wu AW, Carducci MA, Snyder CF. What can I do? Recommendations for responding to issues identified by patient-reported outcomes assessments used in clinical practice. *The journal of supportive oncology.* 2012;10(4):143-148.
51. Cortez NG, Cohen IG, Kesselheim AS. FDA regulation of mobile health technologies. *The New England journal of medicine.* 2014;371(4):372-379.
52. Brundage M, Blackford A, Tolbert E, et al. Presenting comparative study PRO results to clinicians and researchers: beyond the eye of the beholder. *Quality of life research : an international journal of quality of life aspects of treatment, care and rehabilitation.* 2017.
53. O'Connor S, Hanlon P, O'Donnell CA, Garcia S, Glanville J, Mair FS. Barriers and facilitators to patient and public engagement and recruitment to digital health interventions: protocol of a systematic review of qualitative studies. *BMJ Open.* 2016;6(9):e010895.
54. Snyder CF, Smith KC, Bantug ET, et al. What do these scores mean? Presenting patient-reported outcomes data to patients and clinicians to improve interpretability. *Cancer.* 2017;123(10):1848-1859.
55. Jensen RE, Snyder CF, Basch E, Frank L, Wu AW. All together now: findings from a PCORI workshop to align patient-reported outcomes in the electronic health record. *J Comp Eff Res.* 2016;5(6):561-567.
56. Damschroder LJ, Aron DC, Keith RE, Kirsh SR, Alexander JA, Lowery JC. Fostering implementation of health services research findings into practice: a consolidated framework for advancing implementation science. *Implement Sci.* 2009;4:50.
57. Halpern MT, Zhang F, Enewold L. Hospitalizations following cancer diagnosis: National values for frequency, duration, and charges. *Journal of Clinical Oncology.* 2020;38(15_suppl):12039-12039.
58. Meira D, Lavoura P, Ferreira D, et al. Impact of hospitalization in the functionality and quality of life of adults and elderlies. *European Respiratory Journal.* 2015;46(suppl 59):PA3547.
59. Parikh RB, Manz C, Chivers C, et al. Machine Learning Approaches to Predict 6-Month Mortality Among Patients With Cancer. *JAMA Netw Open.* 2019;2(10):e1915997.
60. Sahni N, Simon G, Arora R. Development and Validation of Machine Learning Models for Prediction of 1-Year Mortality Utilizing Electronic Medical Record Data Available at the End of Hospitalization in Multicondition Patients: a Proof-of-Concept Study. *J Gen Intern Med.* 2018;33(6):921-928.
61. Obermeyer Z, Powers B, Vogeli C, Mullainathan S. Dissecting racial bias in an algorithm used to manage the health of populations. *Science.* 2019;366(6464):447-453.

62. Parikh RB, Schnall J, Liu M, et al. Augmenting machine learning algorithms to predict mortality using patient-reported outcomes in oncology. *Journal of Clinical Oncology.* 2021;39(15_suppl):1510-1510.
63. Thompson M, Tiwari A, Fu R, Moe E, Buckley DI. In: *A Framework To Facilitate the Use of Systematic Reviews and Meta-Analyses in the Design of Primary Research Studies.* Rockville (MD)2012.
64. Ariti C. Walter W Stroup, Generalized linear mixed models, modern concepts, methods and applications. Stroup Walter W , Generalized linear mixed models, modern concepts, methods and applications . CRC Press: Boca Raton, 2012; pound59.99 p. 555. *Statistical methods in medical research.* 2017;26(2):1043-1044.
65. Schafer JL. Multiple imputation: a primer. *Stat Methods Med Res.* 1999;8(1):3-15.
66. Hemming K, Taljaard M. Sample size calculations for stepped wedge and cluster randomised trials: a unified approach. *Journal of clinical epidemiology.* 2016;69:137-146.
67. Eldridge SM, Costelloe CE, Kahan BC, Lancaster GA, Kerry SM. How big should the pilot study for my cluster randomised trial be? *Statistical methods in medical research.* 2016;25(3):1039-1056.
68. Campbell MK, Mollison J, Grimshaw JM. Cluster trials in implementation research: estimation of intracluster correlation coefficients and sample size. *Statistics in medicine.* 2001;20(3):391-399.
69. Yost KJ, Eton DT, Garcia SF, Cella D. Minimally important differences were estimated for six Patient-Reported Outcomes Measurement Information System-Cancer scales in advanced-stage cancer patients. *Journal of clinical epidemiology.* 2011;64(5):507-516.
70. Temel JS, Greer JA, El-Jawahri A, et al. Effects of Early Integrated Palliative Care in Patients With Lung and GI Cancer: A Randomized Clinical Trial. *Journal of clinical oncology : official journal of the American Society of Clinical Oncology.* 2017;35(8):834-841.
71. Chen CX, Kroenke K, Stump TE, et al. Estimating minimally important differences for the PROMIS(R) Pain Interference Scales: results from three randomized clinical trials. *Pain.* 2017.
72. Analyzing CAHPS Survey Data. Agency for Healthcare Research and Quality. https://www.ahrq.gov/cahps/surveys-guidance/helpful-resources/analysis/index.html. Published 2016. Accessed Accessed on 1/3/18.
73. Xie T, Waksman J. Design and sample size estimation in clinical trials with clustered survival times as the primary endpoint. *Statistics in medicine.* 2003;22(18):2835-2846.
74. Valderas JM, Kotzeva A, Espallargues M, et al. The impact of measuring patient-reported outcomes in clinical practice: a systematic review of the literature. *Quality of life research : an international journal of quality of life aspects of treatment, care and rehabilitation.* 2008;17(2):179-193.
75. Weiner BJ, Lewis CC, Stanick C, et al. Psychometric assessment of three newly developed implementation outcome measures. *Implementation science : IS.* 2017;12(1):108.
76. Fetters MD, Curry LA, Creswell JW. Achieving integration in mixed methods designs-principles and practices. *Health services research.* 2013;48(6 Pt 2):2134-2156.

77. Ritchie J SL. *Qualitative data analysis for applied policy research.* London: Routledge: Analyzing Qualitative Data.; 1994.
78. Pope C, Ziebland S, Mays N. Qualitative research in health care. Analysing qualitative data. *BMJ (Clinical research ed).* 2000;320(7227):114-116.
79. Richie JP SL. *Qualitative Data Analysis for Applied Policy Research in The Qualitative Researchers Companion.* London: Sage Publications 2002.
80. Creswell JW. *Qualitative Inquiry & Research Design: Choosing among Five Approached.* Sage Publications; 2013.
81. Damschroder LJ, Lowery JC. Evaluation of a large-scale weight management program using the consolidated framework for implementation research (CFIR). *Implementation science : IS.* 2013;8:51.
82. Wong SL, Revels SL, Yin H, Stewart AK, McVeigh A, Banerjee M, Birkmeyer JD. Variation in hospital mortality rates with inpatient cancer surgery. Ann Surg. 2015;261(4):632-6. PMID: 24743604.
83. Siegel RL, Miller KD, Jemal A. Cancer Statistics, 2017. CA Cancer J Clin. 2017;67(1):7-30. Epub 2017/01/06. PMID: 28055103.
84. Janjan N. Palliation and supportive care in radiation medicine. Hematol Oncol Clin North Am. 2006;20(1):187-211. PMID: 16580563.
85. Fleishman SB. Treatment of symptom clusters: pain, depression, and fatigue. J Natl Cancer Inst Monogr. 2004(32):119-23. PMID: 15263052.
86. Hernandez-Boussard T, Graham LA, Desai K, Wahl TS, Aucoin E, Richman JS, Morris MS, Itani KM, Telford GL, Hawn MT. The Fifth Vital Sign: Postoperative Pain Predicts 30-day Readmissions and Subsequent Emergency Department Visits. Ann Surg. 2017;266(3):516-24. Epub 2017/06/29. PMID: 28657940.
87. Kenzik KM, Ganz PA, Martin MY, Petersen L, Hays RD, Arora N, Pisu M. How much do cancer-related symptoms contribute to health-related quality of life in lung and colorectal cancer patients? A report from the Cancer Care Outcomes Research and Surveillance (CanCORS) Consortium. Cancer. 2015;121(16):2831-9. PMID: 25891437.
88. Levit L, Balogh E, Nass S, Ganz PA, editors. Delivering High-Quality Cancer Care: Charting a New Course for a System in Crisis. Washington, DC; 2013.
89. Gilligan T, Coyle N, Frankel RM, Berry DL, Bohlke K, Epstein RM, Finlay E, Jackson VA, Lathan CS, Loprinzi CL, et al. Patient-Clinician Communication: American Society of Clinical Oncology Consensus Guideline. J Clin Oncol. 2017;35(31):3618-32. PMID: 28892432.
90. Mearis M, Shega JW, Knoebel RW. Does adherence to National Comprehensive Cancer Network guidelines improve pain-related outcomes? An evaluation of inpatient cancer pain management at an academic medical center. J Pain Symptom Manage. 2014;48(3):451-8. PMID: 24439844.
91. Coolbrandt A, Wildiers H, Aertgeerts B, Van der Elst E, Laenen A, Dierckx de Casterle B, van Achterberg T, Milisen K. Characteristics and effectiveness of complex nursing interventions aimed at reducing symptom burden in adult patients treated with chemotherapy: a systematic review of randomized controlled trials. Int J Nurs Stud. 2014;51(3):495-510. PMID: 24074939.

92. Neo J, Fettes L, Gao W, Higginson IJ, Maddocks M. Disability in activities of daily living among adults with cancer: A systematic review and meta-analysis. Cancer Treat Rev. 2017;61:94-106. PMID: 29125982.
93. Valuck T, Blaisdell D, Dugan DP, Westrich K, Dubois RW, Miller RS, McClellan M. Improving Oncology Quality Measurement in Accountable Care: Filling Gaps with Cross-Cutting Measures. J Manag Care Spec Pharm. 2017;23(2):174-81. PMID: 28125364.
94. Patel K, Thoumi A, Nadel J, O'Shea J, McClellan M. Transforming oncology care: payment and delivery reform for person-centered care. Am J Manag Care. 2015;21(5):388-93. PMID: 26167705.
95. Smith G, Bernacki R, Block SD. The role of palliative care in population management and accountable care organizations. J Palliat Med. 2015;18(6):486-94. PMID: 25723619.
96. Mitchell SA, Chambers DA. Leveraging Implementation Science to Improve Cancer Care Delivery and Patient Outcomes. J Oncol Pract. 2017;13(8):523-9. PMID: 28692331.
97. Kotronoulas G, Kearney N, Maguire R, Harrow A, Di Domenico D, Croy S, MacGillivray S. What is the value of the routine use of patient-reported outcome measures toward improvement of patient outcomes, processes of care, and health service outcomes in cancer care? A systematic review of controlled trials. J Clin Oncol. 2014;32(14):1480-501. Epub 2014/04/09. PMID: 24711559.
98. Velikova G, Booth L, Smith AB, Brown PM, Lynch P, Brown JM, Selby PJ. Measuring quality of life in routine oncology practice improves communication and patient well-being: a randomized controlled trial. J Clin Oncol. 2004;22(4):714-24. Epub 2004/02/18. PMID: 14966096.
99. Basch E, Abernethy AP. Supporting clinical practice decisions with real-time patient-reported outcomes. J Clin Oncol. 2011;29(8):954-6. Epub 2011/02/02. PMID: 21282536.
100. Basch E, Deal AM, Dueck AC, Scher HI, Kris MG, Hudis C, Schrag D. Overall Survival Results of a Trial Assessing Patient-Reported Outcomes for Symptom Monitoring During Routine Cancer Treatment. Jama. 2017;318(2):197-8. Epub 2017/06/07. PMID: 28586821.
101. Donovan JL, Hamdy FC, Lane JA, Mason M, Metcalfe C, Walsh E, Blazeby JM, Peters TJ, Holding P, Bonnington S, et al. Patient-Reported Outcomes after Monitoring, Surgery, or Radiotherapy for Prostate Cancer. N Engl J Med. 2016;375(15):1425-37. PMID: 27626365.
102. Grenda TR, Revels SL, Yin H, Birkmeyer JD, Wong SL. Lung Cancer Resection at Hospitals With High vs Low Mortality Rates. JAMA Surg. 2015;150(11):1034-40. PMID: 26267440.
103. Reames BN, Krell RW, Ponto SN, Wong SL. Critical evaluation of oncology clinical practice guidelines. J Clin Oncol. 2013;31(20):2563-8. PMID: 23752105.
104. Brooks GA, Jacobson JO, Schrag D. Clinician perspectives on potentially avoidable hospitalizations in patients with cancer. JAMA Oncol. 2015;1(1):109-10. PMID: 26146663.
105. Brooks GA, Chen EJ, Murakami MA, Giannakis M, Baugh CW, Schrag D. An ED pilot intervention to facilitate outpatient acute care for cancer patients. Am J Emerg Med. 2016;34(10):1934-8. PMID: 27412915.

106. Brooks GA, Li L, Sharma DB, Weeks JC, Hassett MJ, Yabroff KR, Schrag D. Regional variation in spending and survival for older adults with advanced cancer. J Natl Cancer Inst. 2013;105(9):634-42. PMID: 23482657.
107. Brooks GA, Li L, Uno H, Hassett MJ, Landon BE, Schrag D. Acute hospital care is the chief driver of regional spending variation in Medicare patients with advanced cancer. Health Aff (Millwood). 2014;33(10):1793-800. PMID: 25288424.
108. Attai DJ, Sedrak MS, Katz MS, Thompson MA, Anderson PF, Kesselheim JC, Fisch MJ, Graham DL, Utengen A, Johnston C, et al. Social media in cancer care: highlights, challenges & opportunities. Future Oncol. 2016;12(13):1549-52. PMID: 27025657.
109. Han PK, Dieckmann NF, Holt C, Gutheil C, Peters E. Factors Affecting Physicians' Intentions to Communicate Personalized Prognostic Information to Cancer Patients at the End of Life: An Experimental Vignette Study. Med Decis Making. 2016;36(6):703-13. PMID: 26985015.
110. Korir A, Mauti N, Moats P, Gurka MJ, Mutuma G, Metheny C, Mwamba PM, Oyiro PO, Fisher M, Ayers LW, et al. Developing clinical strength-of-evidence approach to define HIV-associated malignancies for cancer registration in Kenya. PLoS One. 2014;9(1):e85881. PMID: 24465764.
111. Shelburne N, Adhikari B, Brell J, Davis M, Desvigne-Nickens P, Freedman A, Minasian L, Force T, Remick SC. Cancer treatment-related cardiotoxicity: current state of knowledge and future research priorities. J Natl Cancer Inst. 2014;106(9). PMID: 25210198.
112. Venook AP, Niedzwiecki D, Lenz HJ, Innocenti F, Fruth B, Meyerhardt JA, Schrag D, Greene C, O'Neil BH, Atkins JN, et al. Effect of First-Line Chemotherapy Combined With Cetuximab or Bevacizumab on Overall Survival in Patients With KRAS Wild-Type Advanced or Metastatic Colorectal Cancer: A Randomized Clinical Trial. Jama. 2017;317(23):2392-401. PMID: 28632865.
113. Sanoff HK, Carpenter WR, Sturmer T, Goldberg RM, Martin CF, Fine JP, McCleary NJ, Meyerhardt JA, Niland J, Kahn KL, et al. Effect of adjuvant chemotherapy on survival of patients with stage III colon cancer diagnosed after age 75 years. J Clin Oncol. 2012;30(21):2624-34. PMID: 22665536.
114. Sanoff HK, Carpenter WR, Freburger J, Li L, Chen K, Zullig LL, Goldberg RM, Schymura MJ, Schrag D. Comparison of adverse events during 5-fluorouracil versus 5-fluorouracil/oxaliplatin adjuvant chemotherapy for stage III colon cancer: a population-based analysis. Cancer. 2012;118(17):4309-20. PMID: 22294436.
115. 2010 Census Urban and Rural Classification and Urban Area Criteria: U.S. Department of Commerce; 2015 [Accessed on 1/1/18]. Available from: https://www.census.gov/geo/reference/ua/urban-rural-2010.html.
116. Murray C. Life Expectancy & Probability of Death Data Visualization: Institute for Health Metrics and Evaluation; 2016 [Accessed on 1/6/18]. Available from: http://vizhub.healthdata.org/le/.
117. Rurality in the United States: Housing Assistance Council; 2011 [Accessed on 12/31/17]. Available from: http://www.ruralhome.org/storage/research_notes/Rural_Research_Note_Rurality_web.pdf.

118. Yen PY, McAlearney AS, Sieck CJ, Hefner JL, Huerta TR. Health Information Technology (HIT) Adaptation: Refocusing on the Journey to Successful HIT Implementation. JMIR Med Inform. 2017;5(3):e28. Epub 2017/09/09. PMID: 28882812.
119. Institute of Medicine Roundtable on Environmental Health Sciences RM. The National Academies Collection: Reports funded by National Institutes of Health. In: Merchant J, Coussens C, Gilbert D, editors. Rebuilding the Unity of Health and the Environment in Rural America: Workshop Summary. Washington (DC): National Academies Press (US), National Academy of Sciences; 2006.
120. About Rural Health Care National Rural Health Association; 2018 [Accessed on 12/30/17]. Available from: https://www.ruralhealthweb.org/about-nrha/about-rural-health-care.
121. Blake KD, Moss JL, Gaysynsky A, Srinivasan S, Croyle RT. Making the Case for Investment in Rural Cancer Control: An Analysis of Rural Cancer Incidence, Mortality, and Funding Trends. Cancer Epidemiol Biomarkers Prev. 2017;26(7):992-7. Epub 2017/06/11. PMID: 28600296.
122. Singh R, Goebel LJ. Rural Disparities in Cancer Care: A Review of Its Implications and Possible Interventions. W V Med J. 2016;112(3):76-82. PMID: 27301159.
123. Clinical Research Resources & Offices: Dana-Farber / Harvard Cancer Center; 2018 [Accessed on 1/6/18]. Available from: http://www.dfhcc.harvard.edu/research/clinical-research-support/.
124. Survey and Data Management Core: Dana-Farber / Harvard Cancer Center; 2018 [cited Accessed on 1/6/18]. Available from: http://www.dfhcc.harvard.edu/research/core-facilities/survey-and-data-management/.
125. Consolidated Framework for Implementation Research: CFIR Research Team; 2009 [Accessed on 1/6/18]. Available from: http://www.cfirguide.org/.
126. Harris PA, Taylor R, Thielke R, Payne J, Gonzalez N, Conde JG. Research electronic data capture (REDCap)--a metadata-driven methodology and workflow process for providing translational research informatics support. J Biomed Inform. 2009;42(2):377-81. PMID: 18929686.
127. REDCap: Research Electronic Data Capture; 2004 [Accessed on 1/6/18]. Available from: https://projectredcap.org/.
128. Caplin M, Saunders T. Utilizing Teach-Back to Reinforce Patient Education: A Step-by-Step Approach. Orthop Nurs. 2015;34(6):365-8; quiz 9-70. PMID: 26575509.
129. Kawachi I. It's All in the Game-The Uses of Gamification to Motivate Behavior Change. JAMA Intern Med. 2017;177(11):1593-4. PMID: 28973152.
130. Chambers DA, Glasgow RE, Stange KC. The dynamic sustainability framework: addressing the paradox of sustainment amid ongoing change. Implement Sci. 2013;8:117. Epub 2013/10/04. PMID: 24088228.
131. Curran GM, Bauer M, Mittman B, Pyne JM, Stetler C. Effectiveness-implementation hybrid designs: combining elements of clinical effectiveness and implementation research to enhance public health impact. Med Care. 2012;50(3):217-26. Epub 2012/02/09. PMID: 22310560.

132. Loudon K, Treweek S, Sullivan F, Donnan P, Thorpe KE, Zwarenstein M. The PRECIS-2 tool: designing trials that are fit for purpose. Bmj. 2015;350:h2147. Epub 2015/05/10. PMID: 25956159.
133. Mohler R, Kopke S, Meyer G. Criteria for Reporting the Development and Evaluation of Complex Interventions in healthcare: revised guideline (CReDECI 2). Trials. 2015;16:204. Epub 2015/05/04. PMID: 25935741.
134. Extensions of the CONSORT Statement: Consort Transparent Reporting of Trials; 2010 [Accessed on 1/6/18]. Available from: http://www.consort-statement.org/extensions.
135. Campbell MK, Piaggio G, Elbourne DR, Altman DG. Consort 2010 statement: extension to cluster randomised trials. Bmj. 2012;345:e5661. Epub 2012/09/07. PMID: 22951546.
136. Grayling MJ, Wason JM, Mander AP. Stepped wedge cluster randomized controlled trial designs: a review of reporting quality and design features. Trials. 2017;18(1):33. Epub 2017/01/23. PMID: 28109321.
137. Thompson M, Tiwari A, Fu R, Moe E, Buckley DI. A Framework To Facilitate the Use of Systematic Reviews and Meta-Analyses in the Design of Primary Research Studies. Rockville (MD)2012.
138. Melamed A, Wright AA. Patient Reported Outcomes: Recent Successes and Future Opportunities. Gynecol Oncol. 2018;148(1):1-2. PMID: 29304953.
139. Wright A, Raman N, Staples P, Schonholz S, Cronin A, Carlson K, Keating N, Onnela J. The HOPE pilot study: harnessing patient-reported outcomes and biometric data to enhance cancer care. . JCO Clin Cancer Inform. In press.
140. Joly F, Hilpert F, Okamoto A, Stuart G, Ochiai K, Friedlander M, of the participants of the 5th Ovarian Cancer Consensus C. Fifth Ovarian Cancer Consensus Conference of the Gynecologic Cancer InterGroup: Recommendations on incorporating patient-reported outcomes in clinical trials in epithelial ovarian cancer. Eur J Cancer. 2017;78:133-8. PMID: 28448857.
141. Reeve BB, Mitchell SA, Dueck AC, Basch E, Cella D, Reilly CM, Minasian LM, Denicoff AM, O'Mara AM, Fisch MJ, et al. Recommended patient-reported core set of symptoms to measure in adult cancer treatment trials. J Natl Cancer Inst. 2014;106(7). PMID: 25006191.
142. Donovan KA, Donovan HS, Cella D, Gaines ME, Penson RT, Plaxe SC, von Gruenigen VE, Bruner DW, Reeve BB, Wenzel L. Recommended patient-reported core set of symptoms and quality-of-life domains to measure in ovarian cancer treatment trials. J Natl Cancer Inst. 2014;106(7). PMID: 25006190.
143. Chen RC, Chang P, Vetter RJ, Lukka H, Stokes WA, Sanda MG, Watkins-Bruner D, Reeve BB, Sandler HM. Recommended patient-reported core set of symptoms to measure in prostate cancer treatment trials. J Natl Cancer Inst. 2014;106(7). PMID: 25006192.
144. Basch E, Rogak LJ, Dueck AC. Methods for Implementing and Reporting Patient-reported Outcome (PRO) Measures of Symptomatic Adverse Events in Cancer Clinical Trials. Clin Ther. 2016;38(4):821-30. Epub 2016/04/06. PMID: 27045992.
145. Cooley ME, Nayak MM, Abrahm JL, Braun IM, Rabin MS, Brzozowski J, Lathan C, Berry DL. Patient and caregiver perspectives on decision support for symptom and

quality of life management during cancer treatment: Implications for eHealth. Psychooncology. 2017;26(8):1105-12. PMID: 28430396.
146. NCI's Comprehensive Database: NIH National Cancer Institute; [Accessed on 1/6/18]. Available from: https://www.cancer.gov/publications/pdq.
147. Symptom & Side Effect Management Cancer Care Ontario; [Accessed on 1/6/18]. Available from: https://www.cancercareontario.ca/en/symptom-management.
148. Navigating Cancer Care: American Society of Clinical Oncology (ASCO); 2005-2018 [1/6/18]. Available from: https://www.cancer.net/navigating-cancer-care/side-effects.
149. Managing Cancer-related Side Effects: American Cancer Society; 2018 [Accessed on 1/6/18]. Available from: https://www.cancer.org/treatment/treatments-and-side-effects/physical-side-effects.html.
150. Kirk MA, Kelley C, Yankey N, Birken SA, Abadie B, Damschroder L. A systematic review of the use of the Consolidated Framework for Implementation Research. Implement Sci. 2016;11:72. Epub 2016/05/18. PMID: 27189233.
151. Sittig DF, Singh H. A new sociotechnical model for studying health information technology in complex adaptive healthcare systems. Qual Saf Health Care. 2010;19 Suppl 3:i68-74. Epub 2010/10/27. PMID: 20959322.
152. Bradley EH, Curry LA, Devers KJ. Qualitative data analysis for health services research: developing taxonomy, themes, and theory. Health Serv Res. 2007;42(4):1758-72. Epub 2007/02/09. PMID: 17286625.
153. Thorne S, Oliffe JL, Stajduhar KI, Oglov V, Kim-Sing C, Hislop TG. Poor communication in cancer care: patient perspectives on what it is and what to do about it. Cancer Nurs. 2013;36(6):445-53. Epub 2013/01/26. PMID: 23348667.
154. Thorne S, Stephens J, Truant T. Building qualitative study design using nursing's disciplinary epistemology. J Adv Nurs. 2016;72(2):451-60. Epub 2015/09/29. PMID: 26412414.
155. CFIR Guide: Consolidated Framework for Implementation Research; 2009 [Accessed on 12/30/17]. Available from: http://www.cfirwiki.net/guide/app/index.html#/.
156. O'Cathain A, Murphy E, Nicholl J. Three techniques for integrating data in mixed methods studies. Bmj. 2010;341:c4587. PMID: 20851841.
157. Wagner EH, Austin BT, Davis C, Hindmarsh M, Schaefer J, Bonomi A. Improving chronic illness care: translating evidence into action. Health Aff (Millwood). 2001;20(6):64-78. PMID: 11816692.
158. Enzinger AC, Wind JK, Frank E, McCleary NJ, Porter L, Cushing H, Abbott C, Cronin C, Enzinger PC, Meropol NJ, et al. A stakeholder-driven approach to improve the informed consent process for palliative chemotherapy. Patient Educ Couns. 2017;100(8):1527-36. PMID: 28359659.
159. Mallow JA, Theeke LA, Theeke E, Mallow BK. Using Multidisciplinary Focus Groups to Inform the Development of mI SMART: A Nurse-Led Technology Intervention for Multiple Chronic Conditions. Nurs Res Pract. 2016;2016:7416728. PMID: 27504199.
160. Rangachari P, Rissing P, Rethemeyer K. Awareness of evidence-based practices alone does not translate to implementation: insights from implementation research. Qual Manag Health Care. 2013;22(2):117-25. PMID: 23542366.

161. Fixsen D, Scott V, Blase K, Naoom S, Wagar L. When evidence is not enough: the challenge of implementing fall prevention strategies. J Safety Res. 2011;42(6):419-22. PMID: 22152258.
162. van Gemert-Pijnen JE, Nijland N, van Limburg M, Ossebaard HC, Kelders SM, Eysenbach G, Seydel ER. A holistic framework to improve the uptake and impact of eHealth technologies. J Med Internet Res. 2011;13(4):e111. PMID: 22155738.
163. Jensen RE, Rothrock NE, DeWitt EM, Spiegel B, Tucker CA, Crane HM, Forrest CB, Patrick DL, Fredericksen R, Shulman LM, et al. The role of technical advances in the adoption and integration of patient-reported outcomes in clinical care. Med Care. 2015;53(2):153-9. PMID: 25588135.
164. Dowding D, Randell R, Gardner P, Fitzpatrick G, Dykes P, Favela J, Hamer S, Whitewood-Moores Z, Hardiker N, Borycki E, et al. Dashboards for improving patient care: review of the literature. Int J Med Inform. 2015;84(2):87-100. PMID: 25453274.
165. Mlaver E, Schnipper JL, Boxer RB, Breuer DJ, Gershanik EF, Dykes PC, Massaro AF, Benneyan J, Bates DW, Lehmann LS. User-Centered Collaborative Design and Development of an Inpatient Safety Dashboard. Jt Comm J Qual Patient Saf. 2017;43(12):676-85. PMID: 29173289.
166. Dykes PC, Rozenblum R, Dalal A, Massaro A, Chang F, Clements M, Collins S, Donze J, Fagan M, Gazarian P, et al. Prospective Evaluation of a Multifaceted Intervention to Improve Outcomes in Intensive Care: The Promoting Respect and Ongoing Safety Through Patient Engagement Communication and Technology Study. Crit Care Med. 2017;45(8):e806-e13. PMID: 28471886.
167. Dykes PC, Stade D, Dalal A, Clements M, Collins S, Chang F, Fladger A, Getty G, Hanna J, Kandala R, et al. Strategies for Managing Mobile Devices for Use by Hospitalized Inpatients. AMIA Annu Symp Proc. 2015;2015:522-31. PMID: 26958185.
168. The pan-Canadian Oncology Symptom Triage and Remote Support (COSTaRS) project: Ottawa Hospital Research Institute; 2017 [Accessed on 1/9/18]. Available from: https://ktcanada.ohri.ca/costars/Research/.
169. Hemming K, Lilford R, Girling AJ. Stepped-wedge cluster randomised controlled trials: a generic framework including parallel and multiple-level designs. Stat Med. 2015;34(2):181-96. Epub 2014/10/28. PMID: 25346484.
170. Girling AJ, Hemming K. Statistical efficiency and optimal design for stepped cluster studies under linear mixed effects models. Stat Med. 2016;35(13):2149-66. Epub 2016/01/11. PMID: 26748662.
171. Hargreaves JR, Copas AJ, Beard E, Osrin D, Lewis JJ, Davey C, Thompson JA, Baio G, Fielding KL, Prost A. Five questions to consider before conducting a stepped wedge trial. Trials. 2015;16:350. Epub 2015/08/19. PMID: 26279013.
172. Salazar MC, Rosen JE, Wang Z, Arnold BN, Thomas DC, Herbst RS, Kim AW, Detterbeck FC, Blasberg JD, Boffa DJ. Association of Delayed Adjuvant Chemotherapy With Survival After Lung Cancer Surgery. JAMA Oncol. 2017;3(5):610-9. PMID: 28056112.
173. Alexander M, Blum R, Burbury K, Coutsouvelis J, Dooley M, Fazil O, Griffiths T, Ismail H, Joshi S, Love N, et al. Timely initiation of chemotherapy: a systematic literature

review of six priority cancers - results and recommendations for clinical practice. Intern Med J. 2017;47(1):16-34. PMID: 27396268.
174. Alexander M, Beattie-Manning R, Blum R, Byrne J, Hornby C, Kearny C, Love N, McGlashan J, McKiernan S, Milar JL, et al. Guidelines for timely initiation of chemotherapy: a proposed framework for access to medical oncology and haematology cancer clinics and chemotherapy services. Intern Med J. 2016;46(8):964-9. PMID: 27553996.
175. Malietzis G, Mughal A, Currie AC, Anyamene N, Kennedy RH, Athanasiou T, Jenkins JT. Factors Implicated for Delay of Adjuvant Chemotherapy in Colorectal Cancer: A Meta-analysis of Observational Studies. Ann Surg Oncol. 2015;22(12):3793-802. PMID: 25777086.
176. Ettinger DS, Wood DE, Aisner DL, Akerley W, Bauman J, Chirieac LR, D'Amico TA, DeCamp MM, Dilling TJ, Dobelbower M, et al. Non-Small Cell Lung Cancer, Version 5.2017, NCCN Clinical Practice Guidelines in Oncology. J Natl Compr Canc Netw. 2017;15(4):504-35. PMID: 28404761.
177. Benson AB, 3rd, Venook AP, Cederquist L, Chan E, Chen YJ, Cooper HS, Deming D, Engstrom PF, Enzinger PC, Fichera A, et al. Colon Cancer, Version 1.2017, NCCN Clinical Practice Guidelines in Oncology. J Natl Compr Canc Netw. 2017;15(3):370-98. PMID: 28275037.
178. Dillman DA SJ, Christian LM. . Internet, mail and mixed mode surveys: the tailored design method 2014 [Accessed on 12/30/17]. Available from: http://www.wiley.com/WileyCDA/WileyTitle/productCd-1118456149.html.
179. Dillman DA, Smyth JD. Design effects in the transition to web-based surveys. Am J Prev Med. 2007;32(5 Suppl):S90-6. PMID: 17466824.
180. CAHPS® Cancer Care Survey: Agency for Healthcare Research and Quality; 2016 [Accessed on 12/30/17]. Available from: https://www.ahrq.gov/sites/default/files/wysiwyg/cahps/surveys-guidance/cancer/drug-eng-cancer-552a.pdf.
181. Cancer Surgery: Agency for Healthcare and Quality; 2016 [Accessed on 12/30/17]. Available from: https://www.ahrq.gov/sites/default/files/wysiwyg/cahps/surveys-guidance/cancer/surg-eng-cancer-553a.pdf.
182. CAHPS®: Assessing Health Care Quality From the Patient's Perspective: Agency for Healthcare Research and Quality; 2016 [Accessed on 12/30/17]. Available from: https://www.ahrq.gov/cahps/about-cahps/cahps-program/cahps_brief.html.
183. Bergeson SC, Gray J, Ehrmantraut LA, Laibson T, Hays RD. Comparing Web-based with Mail Survey Administration of the Consumer Assessment of Healthcare Providers and Systems (CAHPS((R))) Clinician and Group Survey. Prim Health Care. 2013;3. Epub 2013/10/01. PMID: 24078901.
184. Drake KM, Hargraves JL, Lloyd S, Gallagher PM, Cleary PD. The effect of response scale, administration mode, and format on responses to the CAHPS Clinician and Group survey. Health Serv Res. 2014;49(4):1387-99. Epub 2014/01/30. PMID: 24471975.
185. Stucky BD, Hays RD, Edelen MO, Gurvey J, Brown JA. Possibilities for Shortening the CAHPS Clinician and Group Survey. Med Care. 2016;54(1):32-7. Epub 2015/11/05. PMID: 26536332.

186. Fielding the CAHPS® Cancer Care Survey 2017 [Accessed on 12/30/17]. Available from: https://www.ahrq.gov/sites/default/files/wysiwyg/cahps/surveys-guidance/cancer/fielding-cancer-53.pdf.

187. Dudgeon D, King S, Howell D, Green E, Gilbert J, Hughes E, Lalonde B, Angus H, Sawka C. Cancer Care Ontario's experience with implementation of routine physical and psychological symptom distress screening. Psychooncology. 2012;21(4):357-64. Epub 2011/02/11. PMID: 21308858.

188. Key Drivers and Change Package: Oncology Care Model; 2017 [Accessed on 12/30/17]. Available from: https://innovation.cms.gov/Files/x/ocm-keydrivers-changepkg.pdf.

189. Oncology Care Model: U.S. Centers for Medicare & Medicaid Services; 2017 [Accessed on 12/8/2017]. Available from: https://innovation.cms.gov/initiatives/oncology-care.

190. SEDD Database Documentation Rockville, MD: Agency for Healthcare Research and Quality; 2017 [Accessed on 1/9/18]. Available from: https://www.hcup-us.ahrq.gov/db/state/sedddbdocumentation.jsp.

191. SID Database Documentation Rockville, MD: Agency for Healthcare Research and Quality; 2017 [Accessed on 1/9/18]. Available from: https://www.hcup-us.ahrq.gov/db/state/siddbdocumentation.jsp.

192. Central Distributor HCUP: Availability of HCUP Databases: Agency for Healthcare Research and Quality; 2017 [Accessed on 1/9/18]. Available from: https://www.hcup-us.ahrq.gov/db/availability_public.jsp.

193. Proctor E, Silmere H, Raghavan R, Hovmand P, Aarons G, Bunger A, Griffey R, Hensley M. Outcomes for implementation research: conceptual distinctions, measurement challenges, and research agenda. Adm Policy Ment Health. 2011;38(2):65-76. Epub 2010/10/20. PMID: 20957426.

194. Stetler CB, Legro MW, Wallace CM, Bowman C, Guihan M, Hagedorn H, Kimmel B, Sharp ND, Smith JL. The role of formative evaluation in implementation research and the QUERI experience. J Gen Intern Med. 2006;21 Suppl 2:S1-8. Epub 2006/04/28. PMID: 16637954.

195. Evans A GD, Maher L. British National Health Service Sustainability Model Version 1.0 (Hybrid Conjoint): British National Health Service Modernization Agency; 2002.

196. Fetters MD, Curry LA, Creswell JW. Achieving integration in mixed methods designs-principles and practices. Health Serv Res. 2013;48(6 Pt 2):2134-56. PMID: 24279835.

197. Powell BJ, Waltz TJ, Chinman MJ, Damschroder LJ, Smith JL, Matthieu MM, Proctor EK, Kirchner JE. A refined compilation of implementation strategies: results from the Expert Recommendations for Implementing Change (ERIC) project. Implement Sci. 2015;10:21. Epub 2015/04/19. PMID: 25889199.

198. Welcome to RE-AIM.org! : RE-AIM; 2018 [Accessed on 1/8/18]. Available from: http://re-aim.org/.

199. Zhang B LW, Jahanzeb M, and Hassett MJ. Use of patient-reported outcome measures in Quality Onocology Practice Initiative registered practices: Results of a national survey. March, 2017; Orlando, FL.

200. Pusic AL, Matros E, Fine N, Buchel E, Gordillo GM, Hamill JB, Kim HM, Qi J, Albornoz C, Klassen AF, et al. Patient-Reported Outcomes 1 Year After Immediate

Breast Reconstruction: Results of the Mastectomy Reconstruction Outcomes Consortium Study. J Clin Oncol. 2017;35(22):2499-506. PMID: 28346808.
201. Davis J. Epic tops 2017 Best in KLAS awards, securing top spot for 7th straight year; see complete winners: Healthcare IT News; 2017 [Accessed on 1/6/18]. Available from: http://www.healthcareitnews.com/news/epic-tops-2017-best-klas-awards-securing-top-spot-7th-straight-year-see-complete-winners.
202. Rigby M, Magrabi F, Scott P, Doupi P, Hypponen H, Ammenwerth E. Steps in Moving Evidence-Based Health Informatics from Theory to Practice. Healthc Inform Res. 2016;22(4):255-60. PMID: 27895956.
203. Harrington L CHC. Usability Evaluation Handbook for Electronic Health Records: HIMSS; 2014.
204. Daigrepont J E, CAPPM, McGrath D, CRNP. . Complete Guide and Toolkit to Successful EHR Adoption: HIMSS; 2011.
205. Krug S. Rocket Surgery Made Easy: The Do-It-Yourself Guide to Finding and Fixing Usability Problems: New Riders; 2009.
206. Bennett AV, Dueck AC, Mitchell SA, Mendoza TR, Reeve BB, Atkinson TM, Castro KM, Denicoff A, Rogak LJ, Harness JK, et al. Mode equivalence and acceptability of tablet computer-, interactive voice response system-, and paper-based administration of the U.S. National Cancer Institute's Patient-Reported Outcomes version of the Common Terminology Criteria for Adverse Events (PRO-CTCAE). Health Qual Life Outcomes. 2016;14:24. Epub 2016/02/20. PMID: 26892667.
207. Mendoza TR, Dueck AC, Bennett AV, Mitchell SA, Reeve BB, Atkinson TM, Li Y, Castro KM, Denicoff A, Rogak LJ, et al. Evaluation of different recall periods for the US National Cancer Institute's PRO-CTCAE. Clin Trials. 2017;14(3):255-63. Epub 2017/05/27. PMID: 28545337.
208. Ross TR, Ng D, Brown JS, Pardee R, Hornbrook MC, Hart G, Steiner JF. The HMO Research Network Virtual Data Warehouse: A Public Data Model to Support Collaboration. EGEMS (Wash DC). 2014;2(1):1049. PMID: 25848584.
209. Common Data Element (CDE) Resource Portal: NIH U.S. National Library of Medicine; 2012 [Accessed on 1/7/18]. Available from: http://cde.nih.gov.
210. Hussey MA, Hughes JP. Design and analysis of stepped wedge cluster randomized trials. Contemp Clin Trials. 2007;28(2):182-91. Epub 2006/07/11. PMID: 16829207.
211. Hemming K, Haines TP, Chilton PJ, Girling AJ, Lilford RJ. The stepped wedge cluster randomised trial: rationale, design, analysis, and reporting. Bmj. 2015;350:h391. Epub 2015/02/11. PMID: 25662947.
212. Ariti C. Walter W Stroup, Generalized linear mixed models, modern concepts, methods and applications. Stroup Walter W , Generalized linear mixed models, modern concepts, methods and applications. CRC Press: Boca Raton, 2012; pound59.99 p. 555. Stat Methods Med Res. 2017;26(2):1043-4. Epub 2015/01/01. PMID: 25549967.
213. Schafer JL. Multiple imputation: a primer. Stat Methods Med Res. 1999;8(1):3-15. Epub 1999/05/29. PMID: 10347857.
214. Hemming K, Taljaard M. Sample size calculations for stepped wedge and cluster randomised trials: a unified approach. J Clin Epidemiol. 2016;69:137-46. Epub 2015/09/08. PMID: 26344808.

215. Eldridge SM, Costelloe CE, Kahan BC, Lancaster GA, Kerry SM. How big should the pilot study for my cluster randomised trial be? Stat Methods Med Res. 2016;25(3):1039-56. Epub 2015/06/14. PMID: 26071431.
216. Campbell MK, Mollison J, Grimshaw JM. Cluster trials in implementation research: estimation of intracluster correlation coefficients and sample size. Stat Med. 2001;20(3):391-9. Epub 2001/02/17. PMID: 11180309.
217. Yost KJ, Eton DT, Garcia SF, Cella D. Minimally important differences were estimated for six Patient-Reported Outcomes Measurement Information System-Cancer scales in advanced-stage cancer patients. J Clin Epidemiol. 2011;64(5):507-16. Epub 2011/03/31. PMID: 21447427.
218. Temel JS, Greer JA, El-Jawahri A, Pirl WF, Park ER, Jackson VA, Back AL, Kamdar M, Jacobsen J, Chittenden EH, et al. Effects of Early Integrated Palliative Care in Patients With Lung and GI Cancer: A Randomized Clinical Trial. J Clin Oncol. 2017;35(8):834-41. Epub 2016/12/29. PMID: 28029308.
219. Chen CX, Kroenke K, Stump TE, Kean J, Carpenter JS, Krebs EE, Bair MJ, Damush TM, Monahan PO. Estimating minimally important differences for the PROMIS(R) Pain Interference Scales: results from three randomized clinical trials. Pain. 2017. Epub 2017/12/05. PMID: 29200181.
220. Analyzing CAHPS Survey Data: Agency for Healthcare Research and Quality; 2016 [Accessed on 1/3/18]. Available from: https://www.ahrq.gov/cahps/surveys-guidance/helpful-resources/analysis/index.html.
221. Xie T, Waksman J. Design and sample size estimation in clinical trials with clustered survival times as the primary endpoint. Stat Med. 2003;22(18):2835-46. Epub 2003/09/04. PMID: 12953283.
222. Ritchie JP, Spencer L. Qualitative data analysis for applied policy research. London: Analyzing Qualitative Data; 1994.
223. Pope C, Ziebland S, Mays N. Qualitative research in health care. Analysing qualitative data. BMJ. 2000;320(7227):114-6. PMID: 10625273.
224. Richie JP, Spencer L. Qualitative Data Analysis for Applied Policy Research in The Qualitative Researchers Companion. Huberman AMaM, M.B., editor. London: Sage Publications; 2002.
225. Creswell JW. Qualitative Inquiry & Research Design: Choosing among Five Approached: Sage Publications; 2013.
226. Damschroder LJ, Lowery JC. Evaluation of a large-scale weight management program using the consolidated framework for implementation research (CFIR). Implement Sci. 2013;8:51. Epub 2013/05/15. PMID: 23663819.
227. Preacher KJ, Hayes AF. SPSS and SAS procedures for estimating indirect effects in simple mediation models. Behav Res Methods Instrum Comput. 2004 Nov;36(4):717-31.
228. Rao AD, Sugar EA, Chang DT, Goodman KA, Hacker-Prietz A, Rosati LM, Columbo L, O'Reilly E, Fisher GA, Zheng L, et al. Patient-reported outcomes of a multicenter phase 2 study investigating gemcitabine and stereotactic body radiation therapy in locally advanced pancreatic cancer. Pract Radiat Oncol. 2016;6(6):417-24. PMID: 27552809.
229. Ribi K, Luo W, Bernhard J, Francis PA, Burstein HJ, Ciruelos E, Bellet M, Pavesi L, Lluch A, Visini M, et al. Adjuvant Tamoxifen Plus Ovarian Function Suppression Versus

Tamoxifen Alone in Premenopausal Women With Early Breast Cancer: Patient-Reported Outcomes in the Suppression of Ovarian Function Trial. J Clin Oncol. 2016;34(14):1601-10. PMID: 27022111.
230. Lee SJ, Logan B, Westervelt P, Cutler C, Woolfrey A, Khan SP, Waller EK, Maziarz RT, Wu J, Shaw BE, et al. Comparison of Patient-Reported Outcomes in 5-Year Survivors Who Received Bone Marrow vs Peripheral Blood Unrelated Donor Transplantation: Long-term Follow-up of a Randomized Clinical Trial. JAMA Oncol. 2016;2(12):1583-9. PMID: 27532508.
231. Brown CH, Curran G, Palinkas LA, Aarons GA, Wells KB, Jones L, Collins LM, Duan N, Mittman BS, Wallace A, et al. An Overview of Research and Evaluation Designs for Dissemination and Implementation. Annu Rev Public Health. 2017;38:1-22. PMID: 28384085.
232. Glaze J. Epic Systems draws on literature greats for its next expansion: Wisconsin State Journal; 2015 [1/6/18]. Available from: http://host.madison.com/news/local/govt-and-politics/epic-systems-draws-on-literature-greats-for-its-next-expansion/article_4d1cf67c-2abf-5cfd-8ce1-2da60ed84194.html.
233. Cancer Stat Facts: National Cancer Institute; 2017 [Accessed on 12/29/17]. Available from: https://seer.cancer.gov/statfacts/.
234. Traeger L, McDonnell TM, McCarty CE, Greer JA, El-Jawahri A, Temel JS. Nursing intervention to enhance outpatient chemotherapy symptom management: Patient-reported outcomes of a randomized controlled trial. Cancer. 2015;121(21):3905-13. PMID: 26194461.
235. Khullar OV, Rajaei MH, Force SD, Binongo JN, Lasanajak Y, Robertson S, Pickens A, Sancheti MS, Lipscomb J, Gillespie TW, et al. Pilot Study to Integrate Patient Reported Outcomes After Lung Cancer Operations Into The Society of Thoracic Surgeons Database. Ann Thorac Surg. 2017;104(1):245-53. PMID: 28483154.
236. Shi Q, Wang XS, Vaporciyan AA, Rice DC, Popat KU, Cleeland CS. Patient-Reported Symptom Interference as a Measure of Postsurgery Functional Recovery in Lung Cancer. J Pain Symptom Manage. 2016;52(6):822-31. PMID: 27521528.
237. Frick MA, Vachani CC, Hampshire MK, Bach C, Arnold-Korzeniowski K, Metz JM, Hill-Kayser CE. Survivorship after lower gastrointestinal cancer: Patient-reported outcomes and planning for care. Cancer. 2017;123(10):1860-8. PMID: 28055110.
238. Gilbert A, Ziegler L, Martland M, Davidson S, Efficace F, Sebag-Montefiore D, Velikova G. Systematic Review of Radiation Therapy Toxicity Reporting in Randomized Controlled Trials of Rectal Cancer: A Comparison of Patient-Reported Outcomes and Clinician Toxicity Reporting. Int J Radiat Oncol Biol Phys. 2015;92(3):555-67. PMID: 26068490.
239. Cowan RA, Suidan RS, Andikyan V, Rezk YA, Einstein MH, Chang K, Carter J, Zivanovic O, Jewell EJ, Abu-Rustum NR, et al. Electronic patient-reported outcomes from home in patients recovering from major gynecologic cancer surgery: A prospective study measuring symptoms and health-related quality of life. Gynecol Oncol. 2016;143(2):362-6. PMID: 27637366.
240. Chambers DA, Norton WE. The Adaptome: Advancing the Science of Intervention Adaptation. Am J Prev Med. 2016;51(4 Suppl 2):S124-31. PMID: 27371105.

241. Chambers DA, Feero WG, Khoury MJ. Convergence of Implementation Science, Precision Medicine, and the Learning Health Care System: A New Model for Biomedical Research. Jama. 2016;315(18):1941-2. PMID: 27163980.
242. Interactive presentations, workshops and meetings: Mentimeter; [Accessed on 1/6/18]. Available from: https://www.mentimeter.com/.
243. Johnson KE, Tachibana C, Coronado GD, Dember LM, Glasgow RE, Huang SS, Martin PJ, Richards J, Rosenthal G, Septimus E, et al. A guide to research partnerships for pragmatic clinical trials. Bmj. 2014;349:g6826. PMID: 25446054.
244. Mallow JA, Theeke LA, Barnes ER, Whetsel T, Mallow BK. Using mHealth Tools to Improve Rural Diabetes Care Guided by the Chronic Care Model. Online J Rural Nurs Health Care. 2014;14(1):43-65. PMID: 26029005.
245. Mallow JA, Theeke LA, Long DM, Whetsel T, Theeke E, Mallow BK. Study protocol: mobile improvement of self-management ability through rural technology (mI SMART). Springerplus. 2015;4:423. PMID: 26301170.
246. Smeltzer M, Klesges L, Lee YS, Faris N, Fehnel C, Houston-Harris C, Ray M, Robbins E, Osarogiagbon R. PS01.39: Reach and Adoption of a Surgical Intervention to Improve Pathologic Nodal Staging Across Healthcare Systems within the US Mid-South: Topic: Surgery. J Thorac Oncol. 2016;11(11S):S293. PMID: 27969507.
247. Yu X, Klesges LM, Smeltzer MP, Osarogiagbon RU. Measuring improvement in populations: implementing and evaluating successful change in lung cancer care. Transl Lung Cancer Res. 2015;4(4):373-84. PMID: 26380178.
248. Bartels SJ, Pratt SI, Mueser KT, Forester BP, Wolfe R, Cather C, Xie H, McHugo GJ, Bird B, Aschbrenner KA, et al. Long-term outcomes of a randomized trial of integrated skills training and preventive healthcare for older adults with serious mental illness. Am J Geriatr Psychiatry. 2014;22(11):1251-61. PMID: 23954039.
249. Pratt SI, Naslund JA, Wolfe RS, Santos M, Bartels SJ. Automated telehealth for managing psychiatric instability in people with serious mental illness. J Ment Health. 2015;24(5):261-5. PMID: 24988132.
250. Naslund JA, Aschbrenner KA, Kim SJ, McHugo GJ, Unutzer J, Bartels SJ, Marsch LA. Health behavior models for informing digital technology interventions for individuals with mental illness. Psychiatr Rehabil J. 2017;40(3):325-35. PMID: 28182469.
251. Fioretos M, Schersten B. Establishment of research in primary health care in Greece. Experiences of Greek-Swedish collaboration. Fam Pract. 1991;8(4):347-9. PMID: 1800198.

STATISTICAL ANALYSIS PLAN
for
PROTOCOL ACTIVITY 4
Pragmatic stepped-wedge cluster randomized trial

SIMPRO Research Center: Integration and Implementation of PROs for Symptom Management in Oncology Practice.

VERSION NUMBER: 2.0

DATE: *January 9, 2023*

Sponsor and Coordinating Center:

Dana-Farber/Harvard Cancer Center (DF/HCC)
Department of Medical Oncology
Coordinating Center PI and Technology PI: Michael Hassett
Dana-Farber Cancer Institute
Boston, MA 02215
Email Address: Michael_Hassett@dfci.harvard.edu
Telephone Number: 617-632-4587

CO-STUDY CHAIRS:

Deborah Schrag MD, MPH
Chair, Department of Medicine
Memorial Sloan Kettering Cancer Center
New York City, NY 10065

Raymond Osarogiagbon MD
Director, Thoracic Oncology Research Group
Baptist Memorial Hospital
Covington, Tennessee 38019

Sandra Wong MD
Chair, Department of Surgery
Dartmouth College
Lebanon, NH 03756

STUDY STATISTICIAN:

Hajime Uno, PhD
Dana-Farber Cancer Institute
Boston, MA 02215

FUNDING:

National Cancer Institute, UM1CA233080.

SIGNATURE PAGE

Protocol Title:	SIMPRO Research Center: Integration and Implementation of PROs for Symptom Management in Oncology Practice.
Sponsor Protocol Number:	DF/HCC IRB #18-986R/18-734
sIRB Protocol Number:	WIRB Tracking #20182593; Study #1248093
SAP Version:	Version 2.0
Date:	January 9, 2023

Michael Hassett, MD, MPH<br>Study Chair, Coordinating Center PI	Date
Deborah Schrag, MD, MPH<br>Co-Chair, Co-PI	Date
Raymond Osarogiagbon, MD<br>Co-Chair, Co-PI	Date
Sandra Wong, MD<br>Co-Chair, Co-PI	Date
Hajime Uno, PhD<br>Study Statistician	Date

RECORDS ON REVISIONS

Version	Date for creation or modification	Person in charge	Remarks
Ver. 1.0	July 20, 2023	Hajime Uno	
Ver. 2.0	January 9, 2024	Hajime Uno	

ACRONYMS USED THROUGHOUT SAP:

AD	Absolute Difference
ED	Emergency Department
EDTR	Emergency Department Treat/Release
EHR	Electronic health record
ePRO	Electronic patient-reported outcomes
eSyM	Electronic symptom management system
eSyM+	eSyM intervention condition
eSyM-	eSyM control condition
GI	Gastrointestinal
GLMM	Generalized Linear Mixed-effects Model
Gyn	Gynecologic
ICC	Intra Class Correlation
IRD	Incidence Rate Difference
IRR	Incidence Rate Ratio
MO	Medical Oncology
OR	Odds Ratio
SAP	Statistical Analysis Plan
SASS	Self-efficacy, Attainment of information needs, Symptom burden, and Satisfaction (Research questionnaire)
Surg	Surgery
SW-CRT	Stepped Wedge Cluster Randomized Trial
THOR	Thoracic

Table of Contents

Table of Contents

1. INTRODUCTION 6

2. OBJECTIVES OF SW-RCT 6

3. STUDY DESIGN....6

3.1. RANDOMIZATION 7
3.2. ELIGIBILITY OF SW-RCT PARTICIPANTS 8
3.3. NUMBER OF SUBJECTS 8
3.4. DURATION OF SUBJECT'S PARTICIPATION IN THE STUDY 9
3.5. DURATION ANTICIPATED TO ENROLL ALL STUDY SUBJECTS 9
3.6. EXTENSION OF ACCRUAL PERIOD....9

4. ANALYSIS FOR AIM 2A: HEALTHCARE UTILIZATION, MEASURED BY THE NEED FOR EMERGENCY AND ACUTE CARE....10

4.1. OUTCOMES AND SUMMARY MEASURES 10
4.2. GENERAL ANALYTIC APPROACH....11
4.3. DESCRIPTION OF THE STUDY PARTICIPANTS AND CHARACTERISTICS OF ELIGIBLE EPISODES 16
4.4. MULTILEVEL GENERALIZED LINEAR REGRESSION ANALYSIS 16
4.5. HETEROGENEITY OF EFFECTS AND PRE-SPECIFIED SUBGROUP ANALYSES 18
4.6. SENSITIVITY ANALYSES....19
4.7. POWER CONSIDERATIONS....20
4.8. TABLE SHELL FOR PRESENTING THE PRIMARY ANALYSIS RESULTS 21

5. ANALYSIS FOR AIM 2B: IMPACT ON INITIATION OF ADJUVANT CHEMOTHERAPY AND CHEMOTHERAPY DURATION....22

5.1. OUTCOMES AND SUMMARY MEASURES 22
5.2. DATA ANALYSIS 22
5.3. POWER CONSIDERATIONS....22

6. ANALYSIS FOR AIM 2C: PATIENTS' OUTCOMES, INDICATED BY LEVELS OF SELF-EFFICACY AND SYMPTOM BURDEN 24

6.1. OUTCOMES AND SUMMARY MEASURES 24
6.2. DATA ANALYSIS 24

7. ANALYSIS FOR AIM 2D: PATIENTS' SATISFACTION WITH THEIR CANCER CARE....26

7.1. OUTCOMES AND SUMMARY MEASURES 26
7.2. DATA ANALYSIS 26

8. ANALYSIS FOR AIM 3A: PATIENT ADOPTION, CLINICIAN UTILIZATION, AND THEIR PERSPECTIVES ON APPROPRIATENESS AND ACCEPTABILITY 26

8.1. OUTCOMES AND SUMMARY MEASURES 26
8.2. DATA ANALYSIS 27

9. ANALYSIS FOR AIM 3B: THE SUSTAINABILITY OF EPRO SYMPTOM MANAGEMENT WITHIN A HEALTH SYSTEM 27

10. ANALYSIS FOR AIM 3C (PENETRATION AND SCALABILITY OF EPROS FOR SYMPTOM MANAGEMENT) AND AIM 3D (EXTENT OF ADAPTATION OF EPRO SYSTEMS OVER THE COURSE OF THE IMPLEMENTATION PROCESS) 28

11. REFERENCES....29

1. Introduction

This document describes the statistical analysis plan for the multisite pragmatic stepped-wedge cluster randomized trial (SW-CRT) specified as "Activity 4" in the study protocol *"SIMPRO Research Center: Integration and Implementation of PROs for Symptom Management in Oncology Practice."*

The study protocol consists of four activities. The overall research goals are: (1) to create and refine eSyM, a reporting and management system that integrates ePROs with the EHR; (2) to evaluate the impact of eSyM on patient outcomes, treatment delivery, and healthcare system utilization using a pragmatic cluster randomized study design; and (3) to undertake a systematic, deliberative approach to implementation to allow for the identification of barriers and facilitators that contribute to the adoption and sustainability of eSyM in routine oncology care.

Table 1 shows the Aims for the entire study. The SW-CRT conducted in Activity 4 addresses Aims 2 and 3. *This document covers the statistical analysis plan for Aim 2 and Aim 3 only.*

**Table 1: Aims of the project: "SIMPRO Research Center: Integration and Implementation of PROs for Symptom Management in Oncology Practice."**
**Aim 1:** Adapt existing ePRO symptom management systems and integrate them into the EHR and routine clinical workflow at six health systems. Specifically:<br>Aim 1a. Obtain patient, clinician, staff and leadership input on ePRO form and function<br>Aim 1b. Refine the content and algorithms for self-management, alerts, and feedback<br>Aim 1c. Develop ePRO training materials for patients, clinicians, and staff<br>Aim 1d. Pilot an ePRO symptom manager at test and study sites and prepare an implementation strategy
**Aim 2:** Determine the effectiveness of eSyM (an EHR-integrated ePRO symptom management system) on health outcomes. Specifically:<br>Aim 2a. Healthcare utilization, measured by the need for emergency and acute care<br>Aim 2b. Impact on cancer care delivery, specifically chemotherapy treatment duration and delays<br>Aim 2c. Patients' outcomes, indicated by levels of self-efficacy and symptom burden<br>Aim 2d. Patients' satisfaction with their cancer care
**Aim 3:** Evaluate the facilitators and barriers to implementation of an EHR-integrated ePRO symptom management system from the patient, clinician, and organizational perspectives. Specifically:<br>Aim 3a. Patient adoption (including program feedback and experiences via qualitative interviews), clinician utilization, and their perspectives on appropriateness and acceptability<br>Aim 3b. The sustainability of ePRO symptom management within a health system<br>Aim 3c. Penetration and scalability of ePROs for symptom management<br>Aim 3d. Extent of adaptation of ePRO systems over the course of the implementation process

2. Objectives of SW-RCT

This SW-CRT is conducted to determine the effectiveness of eSyM on health outcomes including healthcare utilization, measured by the need for emergency and acute care; impact on cancer care delivery, specifically chemotherapy treatment duration and delays; patients' outcomes, indicated by levels of self-efficacy and symptom burden; and patients' satisfaction with their cancer care.

3. Study Design

This study employs a type II hybrid effectiveness-implementation stepped wedge cluster randomized design. The research team will partner with software developers at Epic to adapt working ePRO symptom management systems, one in surgical and one in medical oncology, and fully integrate them into the EHR at 6 health systems. After pilot testing, we will conduct a pragmatic stepped wedge cluster randomized trial to measure the effectiveness of the ePRO system on outcomes that matter to patients and clinicians. Throughout, we will evaluate the implementation process to optimize sustainability and generate actionable knowledge that facilitates scaling to other settings. The proposed study is a hybrid

effectiveness-implementation type II trial according to the Curran schema.[1] The study meets PRECIS-2 criteria for pragmatic trials based on scores of 4 or higher in each domain. [2] Reports will adhere to the revised Criteria for Reporting the Development and Evaluation of Complex Interventions in healthcare (CReDECI2), [3] the CONSORT PRO[4] and cluster randomized extensions,[5] and the stepped wedge reporting guidelines proposed by Grayling.[6]

3.1. Randomization

This SW-RCT has 6 steps (figure below), and we randomize 6 sites (**Table 2**) to each of these steps. To ensure that two sites with the same region were not assigned to the same rollout step group (medical oncology live before surgery or surgery live before medical oncology), we employed a stratified randomization by region. As a result, each of the two groups has a site from each of the three regions (Northern, Southern, and Metropolitan). The stepped wedge design includes seven time-periods, including a run-in period (Figure).

Figure: SIMPRO Stepped-Wedge Randomization Schema

Abbreviations: BAPT=Baptist, WVU=West Virginia University, MMC=Maine Medical Center, DHMC=Dartmouth Hitchcock Medical Center, LCI=Lifespan Cancer Institute, DFCI=Dana-Farber Cancer Institute, SIMPRO=Symptom Management IMplementation of Patient Reported Outcomes in Oncology

Sequence	Group	Site	eSyM version	Period 1 Mar '19 - Aug '19	Period 2 Sep '19- Feb '20	Period 3 Mar '20- Aug '20	Period 4 Sep '20 – Feb '21	Period 5 Mar '21- Aug '21	Period 6 Sep '21- Feb '22	Period 7 Mar '22- Aug '22
Med Onc live Before Surgery	Southern	BAPT	Med	Pre-live	Live					
			Surg	Pre-live						Live
	Northern	MMC	Med	Pre-live		Live				
			Surg	Pre-live					Live	
	Metro-politan	DFCI	Med	Pre-live			Live			
			Surg	Pre-live				Live		
Surgery live Before Med Onc	Metro-politan	LCI	Med	Pre-live				Live		
			Surg	Pre-live			Live			
	Northern	DHMC	Med	Pre-live					Live	
			Surg	Pre-live		Live				
	Southern	WVU	Med	Pre-live						Live
			Surg	Pre-live	Live					

Note: The timing of medical and surgical rollouts is not the same at each site in this SW-RCT. The timing of medical and surgical rollouts was determined so that the number of subjects on the intervention and control would be the same in each site, when medical and surgical cohorts were combined.

**Table 2: Six participating sites and Region**	
Site	Region
WVU	Southern
BAPT	
DHMC	Northern
MMC	
DFCI	Metropolitan
LCI	

3.2. Eligibility of SW-RCT participants

- Age ≥ 18 years
- Patients who meet one of the following:
    - Suspected thoracic cancer AND is inpatient following thoracic surgery.
    - Suspected gastrointestinal cancer AND is inpatient following gastrointestinal surgery.
    - Suspected gynecologic cancer AND is inpatient following gynecologic surgery.
    - Diagnosis of thoracic cancer AND scheduled to start a new treatment plan for thoracic cancer.
    - Diagnosis of gastrointestinal cancer AND scheduled to start a new treatment plan for gastrointestinal cancer.
    - Diagnosis of gynecologic cancer AND scheduled to start a new treatment plan for gynecologic cancer.

** Note 1: Patients undergoing thoracic, gynecologic, or gastrointestinal surgery may not be diagnosed with cancer. These patients are still eligible for eSyM usage, questionnaire completion, and medical record abstraction.*
** Note 2: Any patient at any participating site is allowed to be enrolled but those patients who did not meet the criteria listed above will be excluded from the primary analysis. Currently, only two sites are enrolling patients who do not meet the criteria listed above. If appropriate, we will perform the analysis with the data from all participants as exploratory analyses.*

3.3. Number of Subjects

Considering the 2016 tumor registry-reported analytic case volume at each study site for thoracic, GI and Gyn cancers, 12 patients per month per site for each of surgical and medical oncology is a highly conservative estimate of accrual. With 6 sites, assuming equal cluster size, 432 patients will be enrolled both for each period and for each cohort. Thus, a minimum of 6048 patients are expected to be enrolled to the SW-CRT. (**Table 3**) We will not have a cap for the number of participants for each site. Thus, the number of participants at the end of the study will be different from site to site.

Table 3: Conservative estimates of the accrual numbers for the SW-CRT

Site	MO/ Surg	Period 1 (Run-in)	Period 2	Period 3	Period 4	Period 5	Period 6	Period 7	**Total**
BAPT	MO	72	72	72	72	72	72	72	**504**
	Surg	72	72	72	72	72	72	72	**504**
MMC	MO	72	72	72	72	72	72	72	**504**
	Surg	72	72	72	72	72	72	72	**504**
DFCI	MO	72	72	72	72	72	72	72	**504**
	Surg	72	72	72	72	72	72	72	**504**
LCI	MO	72	72	72	72	72	72	72	**504**
	Surg	72	72	72	72	72	72	72	**504**
DHMC	MO	72	72	72	72	72	72	72	**504**
	Surg	72	72	72	72	72	72	72	**504**
WVU	MO	72	72	72	72	72	72	72	**504**
	Surg	72	72	72	72	72	72	72	**504**
**Total**	**MO**	**432**	**432**	**432**	**432**	**432**	**432**	**432**	**6048**
	**Surg**	**432**	**432**	**432**	**432**	**432**	**432**	**432**	

Notes:
- The length of each period is 6 months. The run-in period will be at least 6 months, and it will be adjusted so that the total number of eligible episodes under the control condition can be identical to that under the intervention condition.
- The cells colored by blue or grey indicate the periods where the intervention is rolled out in that hospital.

A subset of eSyM+ patients will be asked to complete a research questionnaire called the "SASS Questionnaire (eSyM+ version)" asking about their **S**elf-efficacy, **A**ttainment of information needs, **S**ymptom burden, and **S**atisfaction with care.

Table 4: SASS Questionnaire accrual numbers (Survey Cohort)

Site	Surgery			Medical Oncology			Totals
	eSyM- Version	eSyM+ Version	eSyM+ Non-Responder Version	eSyM- Version	eSyM+ Version	eSyM+ Non-Responder Version	
Site 1	75	75	15	75	75	15	330
Site 2	75	75	15	75	75	15	330
Site 3	75	75	15	75	75	15	330
Site 4	75	75	15	75	75	15	330
Site 5	75	75	15	75	75	15	330
Site 6	75	75	15	75	75	15	330
Totals	450	450	90	450	450	90	1,980

eSyM+ : Intervention condition
eSyM- : Control condition

The questionnaire will stop being administered once a minimum of 1,980 total surveys have been received in accordance with the above breakdown. (**Table 4**) ** *Total number of SASS participants through surveys can be larger or smaller depending on availability*.

A small subset of eSyM- and eSyM+ patients will be invited to take part in a one-time qualitative interview. Patients may or may not have previously completed SASS or eSyM questionnaires. Interviews will continue until thematic saturation is reached, or until 100 interviews are completed, whichever is reached first.

3.4. Duration of subject's participation in the study

1) Per protocol, eSyM usage continues for up to 60-180 days from the 1st trigger event but can continue indefinitely at the site's discretion.
2) The SASS Questionnaire is a one-time, 20-minute survey administered 30-180 days after surgery or first dose of chemotherapy.
3) The patient qualitative interview is a one-time, 30–60-minute interview administered any time after eSyM assignment.
4) The patients will be followed for outcomes for up to 1-year after chemotherapy starts (for MO) and discharge (for Surg).

3.5. Duration anticipated to enroll all study subjects

We anticipate that it will take four years to complete this activity.

3.6. Extension of accrual period

Due to COVID, the number of observed ER events was smaller than the expected number in three SIMPRO sites as of April 2022. The study team decided to extend accrual an extra 6 months (1 additional study period) to allow for additional event collection and patient recruitment. The extension will also allow sites who recently went live to continue program optimization and stabilization. The figure (below) depicts the revised study design with Period 8.

Sequence	Group	Site	eSyM version	Period 1	Period 2	Period 3	Period 4	Period 5	Period 6	Period 7	Period 8
				Mar '19 - Aug '19	Sep '19- Feb '20	Mar '20- Aug '20	Sep '20 – Feb '21	Mar '21- Aug '21	Sep '21- Feb '22	Mar '22- Aug '22	Sept '22 – Feb '23
Med Onc live Before Surgery	Southern	BAPT	Med	Pre-live	Go-Live – 9/10/2019						
			Surg	Pre-live						Go-Live – 4/25/2022	
	Northern	MMC	Med	Pre-live		Go-Live – 3/16/2020					
			Surg	Pre-live					Go-Live – 10/19/2021		
	Metro-polytan	DFCI	Med	Pre-live			Go-Live – 9/22/2020 (2 clinics), 11/17/2020 (1 clinic)				
			Surg	Pre-live				Go-Live – 5/4/2021 (1 clinic), 6/15/2021 (2 clinics), 10/12/2021 (1 clinic)			
Surgery live Before Med Onc	Metro-polytan	LCI	Med	Pre-live				Go-Live – 9/21/2021			
			Surg	Pre-live			Go-Live – 11/24/2020				
	Northern	DHMC	Med	Pre-live					Go-Live – 11/30/2021		
			Surg	Pre-live		Go-Live – 4/28/2020					
	Southern	WVU	Med	Pre-live						Go-Live – 4/18/2022	
			Surg	Pre-live	Go-Live – 10/25/2019						

4. Analysis for Aim 2a: Healthcare utilization, measured by the need for emergency and acute care

4.1. Outcomes and summary measures

- **Table 5** shows the outcomes for Aim 2 and the summary measure to quantify the treatment effect magnitude for each outcome. The treatment effect magnitude on each outcome will be reported in both absolute and relative terms. terms (CONSORT Checklist Item 17b).[7]
- The primary outcome for Aim 2 is Emergency Department Treat/Release (EDTR) event occurrence status at 30-day post-chemotherapy start date (for MO) or at 30-day post-surgical discharge date (for Surg). The summary measure of this outcome is the proportion of the event occurrence at Day 30.
- Day 1 for each outcome is defined as the date of the initiation of chemotherapy for MO or the date of discharge from hospital (for Surg). However, events occurring on the first day of chemotherapy infusion or the day of discharge after surgery will be not counted as an "event," because they could not have been impacted by the eSyM program. By this definition, there will be no event at Day 1.

Table 5: Outcomes of Aim 2a

Outcome	Type of outcome	Summary measure for each group	Estimand (summary measure for between-group difference)	Cohort
Emergency Department Treat/Release (EDTR) event occurrence status at Day 30 [**primary**]	Binary	Proportion	Absolute Difference (AD) and Odds Ratio (OR)	MO and Surg
EDTR event occurrence status at Day 90	Binary	Proportion	AD and OR	MO and Surg
Number of the EDTR event occurrences during 180-day of follow-up	Count in a given follow-up period	Incidence rate on (0, 180d)	Incident Rate Difference (IRD) and Incidence Rate Ratio (IRR)	MO and Surg
Number of the EDTR event occurrences during 365-day of follow-up	Count in a given follow-up period	Incidence Rate on (0, 365d)	IRD and IRR	MO and Surg
ED-hospitalization event occurrence status at Day 30	Binary	Proportion	AD and OR	MO and Surg
ED-hospitalization event occurrence status at Day 90	Binary	Proportion	AD and OR	MO and Surg

Number of the ED-hospitalization event occurrences during 180-day of follow-up	Count in a given follow-up period	Incidence rate on (0, 180d)	IRD and IRR	MO and Surg
Number of the ED-hospitalization event occurrences during 365-day of follow-up	Count in a given follow-up period	Incidence Rate on (0, 365d)	IRD and IRR	MO and Surg
Occurrence of 1st chemotherapy discontinuation during one-year follow-up period	Time to event	Incidence Rate on (0, 365d)	IRD and IRR	All MO
Occurrence of 1st admission during one-year follow-up period	Time to event	Incidence Rate on (0, 365d)	IRD and IRR	All MO
Occurrence of 1st re-operation during one-year follow-up period	Time to event	Incidence Rate on (0, 365d)	IRD and IRR	All Surg
Occurrence of 1st re-admission during one-year follow-up period	Time to event	Incidence Rate on (0, 365d)	IRD and IRR	All Surg
Death during one-year follow-up period	Time to event	Incidence Rate on (0, 365d)	IRD and IRR	MO and Surg
Composite of EDTR, Hospitalization, and Death event occurrence status at Day 30	Binary	Proportion	Absolute Difference (AD) and Odds Ratio (OR)	MO and Surg
Death during 30-day post-chemotherapy start date (for MO) or at 30-day post-surgical discharge date (for Surg)	Time to event	Incidence Rate on (0, 30d)	IRD and IRR	MO and Surg

Notes:

- The Day 1 is defined the chemotherapy start date for MO and the discharge date for Surgery.
- All outcomes are defined in relation to the date of discharge from hospital (for Surg) or the initiation date of a new chemotherapy regimen (for MO).
- Note that events occurring on the first day of chemotherapy or the day of discharge after surgery will be excluded, because they could not have been impacted by the eSyM program.

4.2. General Analytic Approach

<u>*Medical Oncology and Surgery:*</u>

- The primary analysis will include data from both medical oncology (MO) and surgery (Surg) and estimate a common effect of the intervention in MO and SO. An indicator variable (MO vs. Surg), interaction between MO/Surg and Site, and interaction between MO/Surg and background time trend will be included as covariates for adjustment in multilevel generalized linear regression models.
- As a secondary analysis, for each outcome, the heterogeneity of the intervention effect by MO/Surg will also be assessed by the test for interaction between the intervention (eSyM yes/no) and MO/surg using multilevel generalized linear regression models. In this analysis, the interaction between the intervention and MO/Surg will be added to the model for the primary analysis.
- As another secondary analysis, we will analyze the data from MO and Surg separately and estimate the intervention effect for each outcome. Note that this analysis is similar to the aforementioned secondary analysis including the interaction between the intervention and MO/Surg. While the aforementioned secondary analysis assumes common effects of patients' characteristics (such as age, race, gender and so on) across MO and Surg, this analysis consider these are possibly different between MO and Surg.

<u>*Determination of the intervention and length of follow-up for each episode*</u>*:*

- The presence /absence of the intervention (eSyM+ vs. eSyM-) is determined in a cross-sectional way at the time of the occurrence of the trigger event
- The follow-up period for each episode is one year from the chemotherapy start date (**MO**) and the date of discharge (**Surg**)

- o The intervention determined at the trigger event will never change during the one-year of follow-up period unless another trigger event occurs (see Figure below)

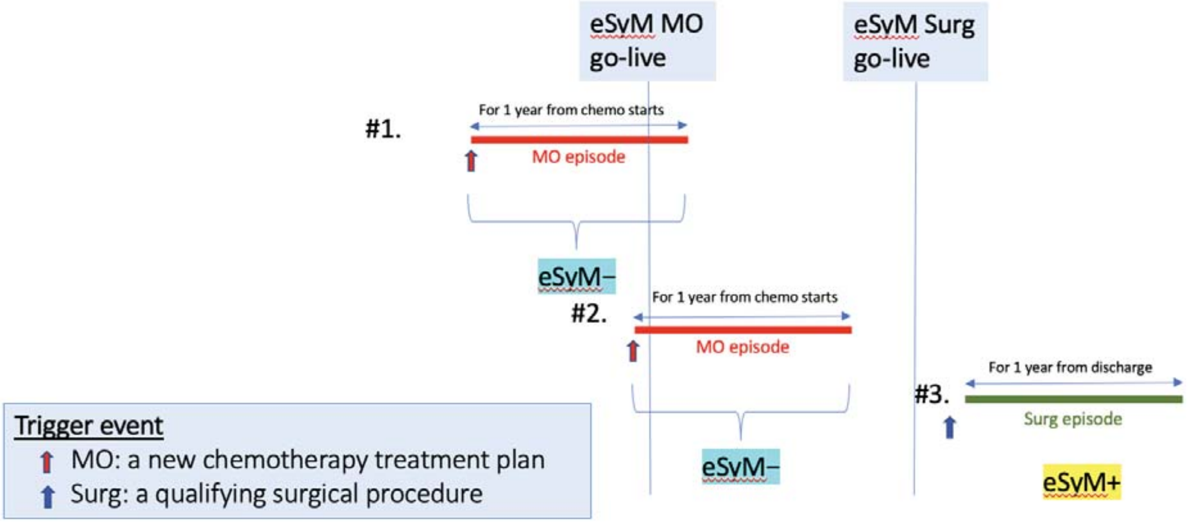

Multiple episodes for medical record data:

- o Some subjects may experience more than one trigger event (i.e., a new chemotherapy treatment plan for **MO** and a qualifying procedure for **Surg**) during the study. Thus, potentially, multiple data points/episodes will be collected from one patient.
- o The primary analysis will include all these episodes other than the following episodes:
  - MO episode where its trigger event occurred after the patient was exposed to eSyM Surg, but before eSyM MO was rolled out in the patient's site.
  - Surg episode where its trigger event occurred after the patient experienced eSyM MO, but before eSyM Surg was rolled out in the patient's site.
  - For example, the 4th episode in the Figure (below) was before go-live of MO in this site and thus this episode is considered eSyM control data(eSyM-) . While this patient did not experience the eSyM MO intervention at that time, this patient already experienced the eSyM Surg intervention (eSyM+) (see the 3rd episode in the figure). Therefore, we do not include the 4th episode as eSyM control (eSyM-) data but exclude it from the primary analysis.

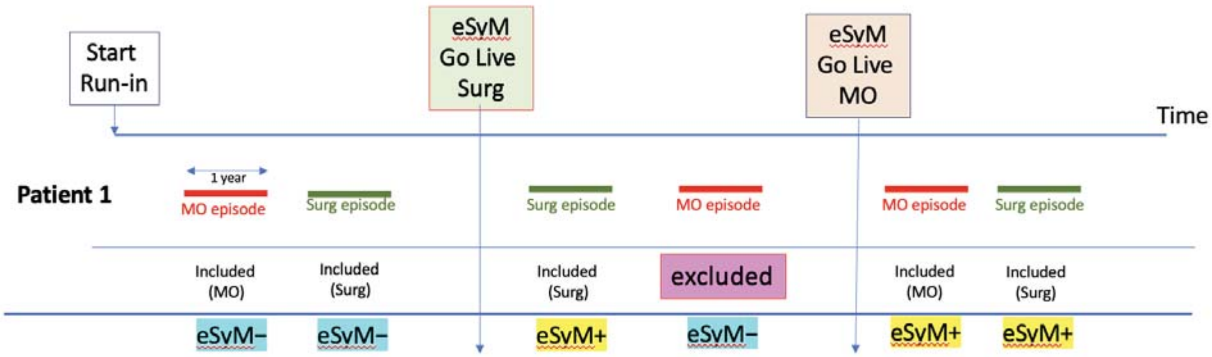

- o As sensitivity analyses, we will perform the analyses using the data consisting of only the first episode from each subject.

<u>Occurrence of another trigger event during the follow-up of an episode</u>:

- If another trigger event (Event B) occurs during the one-year follow-up period of a trigger event (Event A), the data collected after Event B will be included in the aggregation as data of the episode triggered by Event B, not by Event A (Figure below)

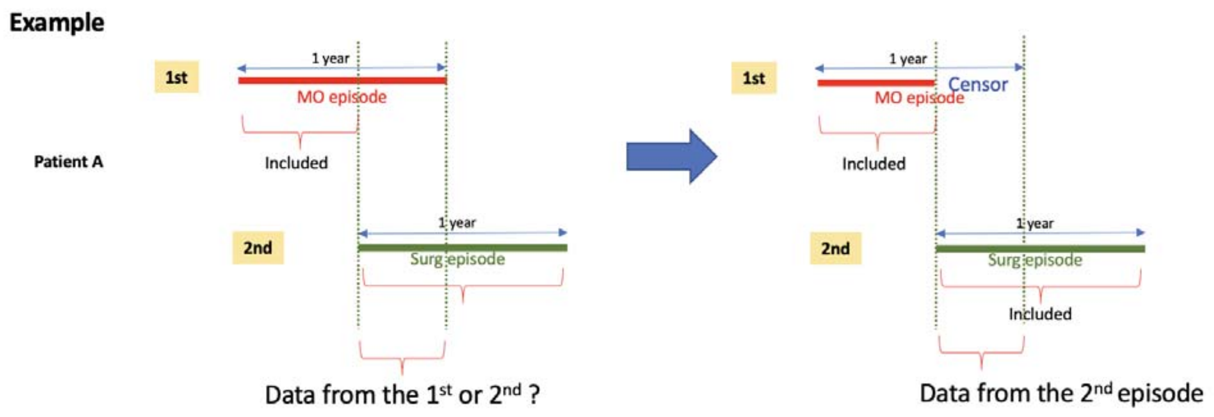

<u>Analysis Populations</u>:

All eligible subjects accrued in this study will be included in the primary analysis. To investigate the impact of patient's engagement with eSyM on clinical outcomes, two additional analysis populations will be considered for secondary and exploratory analyses.

To this end, we consider 3 total groups for analysis:

- Group A: episodes where subjects engaged* with eSyM among the eligible episodes of eligible subjects observed in the eSyM intervention (eSyM+) period.
- Group B: episodes where subjects did not engage with eSyM among the eligible episodes of eligible subjects observed in eSyM+.
- Group C: eligible episodes of eligible subjects observed in the eSyM control (eSyM-) period

* Definition of episodes where patient engaged with eSyM: episodes where patient reports ePRO through eSyM at least once during the follow-up

Table 6 shows the details of each analysis population.

Table 6: Analysis Populations and Objectives

Analysis Population	Comparison	Objective	Notes
1) A+B+C	(A+B) vs. C (Primary analysis)	Estimating the effect of eSyM exposure in a future population similar to the entire study population	Exposed to eSyM vs. Unexposed to eSyM regardless of engagement with the exposure (Effectiveness)<br><br>Clinical outcome data from all groups will be included in the analysis on the basis of the Intention-to-treat principle.
2) A+C*	A vs. C (Pre-specified Secondary)	Estimating the effect of eSyM exposure in a future population that consists of patients who will engage with eSyM upon exposure.	Exposed to eSyM vs. Unexposed to eSyM conditional on engagement with eSyM (Efficacy)<br><br>C* is a subset selected from C, so that the case-mix matches A (i.e., Engagement with eSyM).<br><br>A and B are used to build the propensity score to select C*.
3) A+B	A vs. B (exploratory)	Estimating the effect of eSyM engagement in a future population similar to the study population if everyone engages, under the assumption that everyone in that population engages with eSyM upon exposure.	Engagement with eSyM vs. Unengagement with eSyM, conditioning on everyone being exposed to eSyM<br><br>Propensity score weighting will be used to adjust for case-mix.

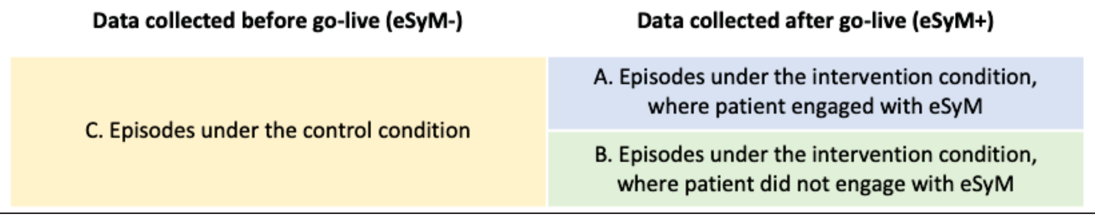

1) The primary analysis population consists of all eligible episodes under the intervention condition (A+B) (i.e., eSyM exposed) and all eligible episodes under the control condition (C) (i.e., eSyM unexposed).
2) The secondary analysis population consists of the episodes where patients engaged with eSyM (A) and the matched episodes during the pre-intervention period (C*). C* is a subset of C. In this analysis, we are interested in estimating the intervention effect among a subgroup of patients who will engage with eSyM if exposed. First, for MO and Surg, a propensity score model (a logistic regression model) to predict engagement with eSyM will be derived using the data from A and B. Next, for each subject in A and C, the predicted probability of engagement will be calculated using the derived propensity score model. The pre-specified variables used for the propensity score models are listed in **Table 7**. For each intervention episode in A, one control episode in C will then be matched, without replacement, based on their predicted probabilities of engagement, using the nearest neighbor matching method stratified by site. The matched dataset is then run through the same outcome model as the primary comparison.
3) As exploratory analyses, we will compare A vs. B. Under the condition that the hospitals have switched on the eSyM system (i.e., everybody is exposed to the eSyM system), we are interested in estimating the effect of the eSyM engagement on the outcomes under the assumption that everyone engages with eSyM, compared to the case where no one engaged with eSyM in spite of eSyM exposure. A propensity score weighting will be used to adjust for the case-mix between engagement with eSyM and non-engagmenet with eSyM. We will build a propensity score model for MO and Surg, separately, using the data from A and B. The pre-specified variables used for the propensity score models are listed in **Table 7**. No outcome model will be involved in this analysis.

**Table 7: Pre-specified variables included in the propensity score models**			
Variable	2) A vs. C*	3) A vs. B	Note
Goal of Chemotherapy (MO with curative goal, MO with palliative goal, Others)	X	X	Only the models for MO
Calendar Time (Secular trends) [continuous with natural cubic spline]		X	
Site (Treating Facility) (DFCI, MMC, DHMC, LCI, BAPT, WVU)	X	X	
Age (year) (fractional polynomials)	X	X	
Sex (male vs. female)	X	X	
Race/Ethnicity (white/non-Hispanic, black/non-Hispanic, Hispanic, Other/Unknown)	X	X	
Employment (Employed, disabled, retired, other)	X	X	
Marital status (married vs. not married)	X	X	
Rurality based on zip code (large metro, small metro, suburban, rural)	X	X	
Insurance type (Medicare only, Medicare and Medicaid, Medicaid only, Private, Other)	X	X	
Cancer type (GI, Gyn, Thoracic)	X	X	
Socio-economic status (quintiles by the zip code-based poverty level)	X	X	
Co-morbidity (Charlson comorbidity index from encounter diagnoses in the 12 months before the first eligibility date) (0, 1, 2+)	X	X	

4.3. Description of the Study Participants and Characteristics of Eligible Episodes

Disposition of the study participants:

Following the CONSORT guideline,[6,8] a CONSORT diagram will be generated to describe the patient population with descriptions of the numbers of participants.

Patient demographic and disease characteristics:

- Baseline patient characteristics and disease characteristics at the start of follow-up will be summarized using descriptive statistics by sequence (i.e., site) .
- The unit of the analysis is not patient but episode. All eligible episodes eSyM- episodes will be included in this analysis.
- The same analysis will be performed for each of the two cohorts (MO and Surg).

The PRECIS score

The Pragmatic-Explanatory Continuum Indicator Summary (PRECIS) score will be calculated and reported.

4.4. Multilevel Generalized Linear Regression Analysis

The primary analysis for the outcomes in Aim 2 (Table 5) will be performed via multilevel generalized linear regression models (or generalized linear mixed-effects model; GLMM).[9] The null hypothesis for the statistical test is no intervention effect on the outcome, which indicates that the regression coefficient for the intervention indicator variable is zero. The intervention effect will be summarized in both absolute and relative terms (CONSORT Checklist Item 17b).[7]

Followings are specifications of the GLMM for the primary and secondary analyses.

- Link function
    - For binary outcomes, the logit-link will be used.
    - For count outcomes, the log-link will be used, and the follow-up time will be included as the offset in the model. (Poisson regression)
    - For time-to-event outcomes, we will use Poisson regression models, where the log-link will be used. The event indicator (1: event, 0: censoring) will be the response variable. Exposure time (i.e., days from the start date of the follow-up to either the date of the event occurrence or the end of follow-up, whichever comes first) will be included as the offset in the model.
- Treatment effect metrics
    - Following the recommendation of the CONSORT guideline, [7] we will report the treatment effect in both absolute and relative metrics.
    - For binary outcomes, we will report odds ratio (OR) derived from the GLMM with the logit-link. In addition, we will calculate absolute difference (AD), using the resulting GLMM with G-computation. Bootstrap will be used to obtain a standard error for the estimated AD.
    - For count outcomes and time-to-event outcomes, we will report the incidence rate ratio (IRR) derived from the GLMM model. In addition, we will calculate absolute difference in incidence rate (i.e., incidence rate difference; IRD) in a similar way to calculate AD.
- Secular trend
    - Primary: Calendar time of the start date of the follow-up of each episode will be included as a continuous variable with natural cubic spline with 3 knots
    - Secondary: Period in which the trigger event occurs will be included as a categorical variable.
- Site (cluster)

    - Since there are only 6 sites in this study, the conventional analysis of including cluster as random-effects is problematic due to this small number.[10]
    - Primary: The primary analysis includes sites as fixed effects.
    - Secondary: Sites will be included as random-effects with a small sample adjustment (the Kenward-Roger method). [11,12]
- Subjects
    - We will potentially have more than one data point from one subject in this study.
    - To take account of within-subject correlation, subjects will be included as random effects.
    - The primary analysis will consider random intercepts only.
- Variables to be included the GLMM
    - **Table 8** lists the variables selected based on the study design (Category A) and clinical importance (Category B and C).
    - The primary analysis includes the variables in Category A and B.
    - As a secondary analysis, we will perform the same analysis including Category A, B and C.

Table 8: Variables that will or may be included in the generalized linear mixed-effects model for the primary analysis

Variable	Category A	Category B	Category C
Intervention indicator: (eSyM+ vs. eSyM-)	X		
Cohort (MO vs. Surgery)	X		
Calendar Time (Secular trends) [continuous with natural cubic spline]	X		
Site (Treating Facility) (DFCI, MMC, DHMC, LCI, BAPT, WVU) [as fixed effects]	X		
Interaction between Cohort (MO vs. Surgery) and Calendar Time (Secular trends)	X		
Interaction between Cohort (MO vs. Surgery) Site (Treating Facility) (DFCI, MMC, DHMC, LCI, BAPT, WVU) [as fixed effects]	X		
Subjects [as random effects (random intercepts only)]	X		
Goal of Chemotherapy (MO with curative goal, MO with palliative goal, Others)		X	
Age (years) (fractional polynomials)		X	
Sex (male vs. female)		X	
Race/Ethnicity (white/non-Hispanic, black/non-Hispanic,<br>Hispanic, Other/Unknown)		X	
Rurality based on zip code (large metro, small metro, suburban, rural)		X	
Cancer/procedure type (GI, Gyn, Thoracic)		X	
Employment (Employed, Disabled, Retired, Other)			X
Marital status (Married vs. Not married)			X
Insurance type (Medicare only, Medicare and Medicaid, Medicaid only, Private, Other)			X
Socio-economic status (quintiles by the zip code-based poverty level)			X
Co-morbidity (Charlson comorbidity index from encounter diagnoses in the 12 months before the first eligibility date) (0, 1, 2+)			X

- Time decay of the intervention effect

- As a sensitivity analysis, we will include the interaction between the intervention and time from the rollout of the intervention condition at the treating site to the time of the trigger event, where we treat the time as a categorical variable in the model.

Considerations on the potential informative censoring

- Death is a competing event for EDTR and Hospitalization. In the primary analyses for EDTR and Admission, those who die without EDTR or Hospitalization will not be excluded from the analyses but included in the denominator when calculating the EDTR rate and Admission rate. Caution should be exercised in interpreting the intervention effect on these non-fatal outcomes. Because those who die before these non-fatal events will never experience it, the intervention effect on these non-fatal events will be biased in favor of either the control or intervention group where more deaths are observed. Therefore, when assessing the effectiveness of the intervention, we will also consider its impact on reducing death (a secondary outcome; see Table 5).
- As a sensitivity analysis for concluding the effectiveness of the intervention, we will analyze the composite of EDTR, Hospitalization, or Death event occurrence status at Day 30 (a secondary outcome; see Table 5), which is not subject to the competing risk problems.

Handling of potentially differentiable follow-up duration across episodes:

- While the planned follow-up duration for each episode is one-year, the actual follow-up durations of some episodes will be shorter than one year due to the occurrence of another trigger event or lost-to-follow-up during the one-year follow-up. Thus, the follow-up duration will be different across the episodes.
- For binary outcomes (i.e., the event status yes/no), the length of follow-up time will not be considered in the analysis. We expect that this will not affect the analysis of the primary endpoint (EDTR at Day 30) because we expect that we will have at least 30 days of follow-up for all episodes. The potential reason of censoring of follow-up is the occurrence of another trigger event during the follow-up time. It is unlikely for a patient to have another trigger event within 30 days after the previous trigger event.
- For count data, we will take the length of the follow-up period for each episode into account. Specifically, we will use Poisson regression models, where the logarithm of follow-up duration of each episode will be included as offset in the models.

4.5. Heterogeneity of effects and pre-specified subgroup analyses

With the small but clinically meaningful effect size we seek to detect, we have insufficient power to evaluate the EDTR rate in all subgroups of interest. Accordingly, subgroup analyses to assess for heterogeneity in treatment effects will be noted as exploratory and limited to pre-specified salient domains. Of greatest interest are:

1. Cohort (MO or Surg);
2. Region of Site (Northern, Southern, or Metropolitan)
3. Age [year] (<70 or >=70);
4. Sex (Male or Female);
5. Race (White/Non-Hispanic or Others);
6. Cancer type (GI, Gyn, or Thoracic); and
7. Rurality inferred from population density in the patient's zip code of residence (large metro, small metro, suburban, or rural).

4.6. Sensitivity analyses

Change in eSyM recall period:

During the study, there was a minor update to the eSyM symptom questionnaire. eSyM originally collected symptom information with a 7-day recall period, but a mid-study update altered this to a 24-hour recall period. Using eSyM+ data only, we will summarize frequencies of severe symptoms reported before the update and after the update. For the evaluation of eSyM intervention, we will not distinguish eSyM intervention before and after the update in the primary analysis. As sensitivity analyses, we will assess the impact of this update on each outcome.

- Sensitivity Analysis 1: We will run the same analysis as the primary analysis, excluding the eSyM+ episodes with a 7-day recall period.
- Sensitivity Analysis 2: We will modify the GLMM model used for the primary analysis. The intervention group variable will be a categorical variable with three levels – control, eSyM+ with 7-day recall, and eSyM+ with 24-hour recall. We will test if there is any difference between the 7-day and 24-hour recall.

Missing data:

Given the short 30-day interval for the Aim 2a primary endpoint and our pragmatic outcome, we expect the dropout fraction and missing data at Day 30 to be negligible. Subjects with no hospital encounters by Day 30 will be included in the analysis as having had no EDTR.

Regarding missing covariates, the primary analysis will be based on the following data handling.

1) Race/Ethnicity: Subjects with missing race/ethnicity are categorized as “Other/Unknown” and included in the analysis.
2) Employment: Subjects with missing employment information are categorized as “Other/Unknown” and included in the analysis.
3) Insurance type: Subjects with missing insurance type are categorized as “Other/Unknown” and included in the analysis.
4) Rurality: Subjects with missing rurality will be excluded from the analysis.
5) Socio-economic status: Subjects with missing socio-economic status are classified as “poverty level 2” and included in the analysis.

Note that the preliminary data check found that the other covariates do not have missing values.

In secondary analysis, we will perform multiple imputations with chained equations for Rurality. Specifically, the imputation models will include the primary outcome (EDTR Day 30), all variables used for the primary analysis (Category A and B), and the variables in C (as auxiliary variables) listed in Table 8. The number of complete datasets we generate will basically the same as the percentage of the fraction of missing. For example, if the missing fraction is 10%, we will generate 10 complete datasets. However, if the fraction of missing is less than 5%, we will generate 5 complete datasets. We will analyze each complete data and obtain the point estimate and the standard error for the intervention effects. We will then pool the results from the multiple complete datasets using Rubin’s method.[13]

Analysis with data from the first episode:

In the primary analysis, all episodes in the analyses except for eSyM- episodes contaminated with the eSyM intervention will be included. We will perform sensitivity analyses using only the first episode from each patient. In these sensitivity analyses, each patient will serve only one data point in the analysis.

Modeling for the COVID19

As a sensitivity analysis, we will include the site-specific COVID19 prevalence as a time-varying covariate in the model.

4.7. Power Considerations

We estimate a minimum of 6048 patients will be enrolled. (see Section 3.3 for details).
Since some subjects may experience more than one trigger event (see Section 4.2 General Analysis Approach: *Multiple episodes for medical record data),* the study participants will potentially contribute multiple data points. The goal of the power calculation for this study is to confirm that the study has sufficient power to assess the effects of the intervention on the outcomes. Therefore, for the power calculations throughout this SAP, we will conservatively estimate the power, assuming that an individual patient provides only one episode.

The probability that a patient who has a trigger event experiences EDTR within 30 days after the trigger event is estimated to vary between 8% to 15% for the control group, based on the HCUP data, institutional data, and early phase analyses from CMMI's Oncology Care Model for Baptist Memorial, the only Oncology Care Model participant among our 6 sites. We hypothesize that the probability of experiencing EDTR by Day 30 will be 3 to 4% lower in the eSyM+ group.

Table 9 shows the required sample sizes for the study to have 80% power to detect the difference between groups, at two-sided alpha level 0.05, using the SW-RCT design.[14] Analyses of inter-institutional variation in hospitalization rates and ED visit rates from AHRQ's statewide databases indicate that low ICC estimates are appropriate.[15,16] Furthermore, we expect a low ICC because: 1) the intervention will be deployed using the same technology across sites; 2) we will adjust for variation in baseline risk via GLMMs such that any potential differences in case-mix among sites will be negligible. Given these factors, the conservative estimate of 6048 participants provides adequate power to address the Aim 2a primary outcome.

Note that we used the sample size formula[14] that assumes exchangeable correlations because we originally had planned to include sites as random effects. However, since the number of sites is only 6 in this study, we modified the analysis plan and decided to include sites as fixed effects in the primary analysis. We acknowledge that our original sample size calculation (above) may not be precise due to this, but it should still work as a conservative estimate.

Table 9. Required total sample size with various scenarios for 80% power

The probability of experiencing EDTR by Day 30		Effect size		N required	
Control (eSyM-)	Intervention (eSyM+)	Absolute	Relative	Low ICC (0.01)	Moderate ICC (0.05)
8%	5%	3%	38%	2192	5844
9%	6%	3%	33%	4485	6816
10%	7%	3%	30%	5047	7721
11%	8%	3%	27%	5598	8606
12%	8%	4%	33%	3246	4810
13%	9%	4%	31%	3538	5283
14%	10%	4%	29%	3823	5746
15%	11%	4%	27%	4103	6198

4.8. Table shell for presenting the primary analysis results

MO and Surgery Combined						
	Number of Observed Events (%)		Odds Ratio		Absolute Difference	
Outcomes	Intervention (#patients=XX) (#episodes = XX)	Control (#patients=XX) (#episodes = XX)	Estimate (0.95 CI)	p-value	Estimate (0.95 CI)	p-value
EDTR Day 30	XX (XX%)	XX (XX%)	X.XX (X.XX to X.XX)	X.XXX	X.XX (X.XX to X.XX)	X.XXX
EDTR Day 90	XX (XX%)	XX (XX%)	X.XX (X.XX to X.XX)	X.XXX	X.XX (X.XX to X.XX)	X.XXX
Admission Day 30	XX (XX%)	XX (XX%)	X.XX (X.XX to X.XX)	X.XXX	X.XX (X.XX to X.XX)	X.XXX
Admission Day 90	XX (XX%)	XX (XX%)	X.XX (X.XX to X.XX)	X.XXX	X.XX (X.XX to X.XX)	X.XXX
MO Only						
	Number of Observed Events (%)		Odds Ratio		Absolute Difference	
Outcomes	Intervention (#patients=XX) (#episodes = XX)	Control (#patients=XX) (#episodes = XX)	Estimate (0.95 CI)	p-value	Estimate (0.95 CI)	p-value
EDTR Day 30	XX (XX%)	XX (XX%)	X.XX (X.XX to X.XX)	X.XXX	X.XX (X.XX to X.XX)	X.XXX
EDTR Day 90	XX (XX%)	XX (XX%)	X.XX (X.XX to X.XX)	X.XXX	X.XX (X.XX to X.XX)	X.XXX
Admission Day 30	XX (XX%)	XX (XX%)	X.XX (X.XX to X.XX)	X.XXX	X.XX (X.XX to X.XX)	X.XXX
Admission Day 90	XX (XX%)	XX (XX%)	X.XX (X.XX to X.XX)	X.XXX	X.XX (X.XX to X.XX)	X.XXX
Surgery Only						
	Number of Observed Events (%)		Odds Ratio		Absolute Difference	
Outcomes	Intervention (#patients = XX) (#episodes = XX)	Control (#patients=XX) (#episodes = XX)	Estimate (0.95 CI)	p-value	Estimate (0.95 CI)	p-value
EDTR Day 30	XX (XX%)	XX (XX%)	X.XX (X.XX to X.XX)	X.XXX	X.XX (X.XX to X.XX)	X.XXX
EDTR Day 90	XX (XX%)	XX (XX%)	X.XX (X.XX to X.XX)	X.XXX	X.XX (X.XX to X.XX)	X.XXX
Admission Day 30	XX (XX%)	XX (XX%)	X.XX (X.XX to X.XX)	X.XXX	X.XX (X.XX to X.XX)	X.XXX
Admission Day 90	XX (XX%)	XX (XX%)	X.XX (X.XX to X.XX)	X.XXX	X.XX (X.XX to X.XX)	X.XXX
Factors used for the adjustment: XXX, XXX, XXX, XXX						

5. Analysis for Aim 2b: Impact on initiation of adjuvant chemotherapy and chemotherapy duration.

5.1. Outcomes and summary measures

We do not have preliminary data to support a specific effect size. However, we expect that patients exposed to eSyM may be able to: 1) initiate adjuvant therapy sooner; and/or 2) remain on their chemotherapy regimens for longer duration. These time intervals are straightforward to measure from EHR encounter and date fields. The outcomes for Aim 2b and the projected minimum sample size are summarized in **Table 10**.

Table 10: Outcomes of Aim 2b

**Outcome**	Type of outcome	Summary measure for each group	Summary measure for between-group difference	**Cohort**	**Projected *minimum* sample size**
Days from chemo regimen start date to stop date	Continuous	Mean*	Absolute Difference (AD)	All MO	504 patients/site; 3024 MO patients in total 1512 eSyM+ and 1512 eSyM-
Days from surgery date to start of adjuvant chemotherapy	Continuous	Mean*	AD	Surg who get adjuvant Rx (roughly 40% of all Surg)	202 patients/site; 1212 surg patients in total 606 eSyM+ and 606 eSyM-

* Because the maximum follow-up period of each episode is 1 year, the data will be truncated at 365 days when the mean value is calculated.

For MO patients, the outcome is time from the first dose to the last dose of a specific regimen. We will censor follow-up at 1 year. For Surg, the denominator population consists of patients who receive *any* adjuvant chemotherapy within 6 postoperative months. Tumor registry stage distribution at our 6 sites indicates that this will be 202 patients per site or 1212 in total.
The type of censoring involved in this analysis is only the truncation at the maximum follow-up period of 1 year. Thus, we will not employ censored time-to-event analysis methods but handle the outcomes as continuous outcomes.

5.2. Data Analysis

Similar analyses to those for Aim 2a (see Section 4) will be performed. GLMMs will be used with the indemnity link function for these continuous variables. The intervention effect will be summarized as the mean difference between eSyM+ and eSyM- and reported with a corresponding 95% confidence interval and p-value.

5.3. Power Considerations

Our general approach is to determine the magnitude of difference detectable at a given sample size. Because we have insufficient preliminary information about the magnitude of the ICCs or standard deviations (SD) for our outcomes, we will consider a broad range.

For MO, assuming the average duration of chemotherapy is 180 days in the eSyM- group, the range of detectable between group differences is shown in **Table 11A**. Based on these very

conservative estimates, we have more than 80% power to detect a 16% increase in chemotherapy duration, which corresponds to nearly 1 month.

For Surg, we assume that the average duration from surgery to adjuvant chemotherapy start date (censored at 6 months) is 56 days (8 weeks) in the eSyM- group. With 1212 patients (see **Table 10**), and conservative parameter estimates, we have >80% power to detect a 25% increase in the interval between surgery and starting adjuvant therapy. This corresponds to 14 days, a difference that is both plausible and meaningful, see **Table 11B.**

Table 11: Power considerations for the outcomes of Aim 2b

**A: MO 3024 patients (1512 per group)**				
Detectable effect size with 80% power	Assumed ICC	Assumed standard deviation [Day]	Difference in time to discontinuation	Corresponding ratio in the eSyM- group
0.138	0.01% (Very low)	202 (conservative)	28 days	1.16
		152	21 days	1.12
		101	14 days	1.08
0.170	1%	165 (conservative)	28 days	1.16
		124	21 days	1.12
		82	14 days	1.08
0.174	5% (conservative)	161 (conservative)	28 days	1.16
		120	21 days	1.12
		80	14 days	1.08
**B: Surg 1212 patients (606 per group)**				
Detectable effect size with 80% power	Assumed ICC	Assumed standard deviation	Difference in time to adjuvant chemo	Corresponding ratio in eSyM- group
0.217	0.01% (Very low)	65 (conservative)	14 days	1.25
		32	7 days	1.13
0.256	1%	55 (conservative)	14 days	1.25
		27	7 days	1.13
0.271	5% (conservative)	52 (conservative)	14 days	1.25
		26	7 days	1.13

6. Analysis for Aim 2c: Patients' outcomes, indicated by levels of self-efficacy and symptom burden

6.1. Outcomes and summary measures

The outcomes investigated in Aim 2c of the SIMPRO study are classified into four groups, depending on the data source and data collection schedule. **Table 12** shows the details of the groups and the disposition of each outcome in Aim 2c.

Table 12: Outcomes in Aim 2c

Type	Outcome Type in the SIMPRO study	Example outcomes in Aim 2c
1	Measured at one single time point only either in post-intervention (the intervention condition) or in pre-intervention (the control condition) for all or selected subjects (patients or providers)	eSyM decline (1st time)
2	Measured at multiple time points (periodically) only in post-intervention period for all or selected subjects (patients or providers)	eSyM logins per patient per month
		Frequency of patient eSyM reports with severe symptoms
3	Measured from selected subjects (patients or providers) at a single time point, which could be during the pre-intervention period or the post-intervention period. For some subjects, data may be collected at both periods.	PROMIS items (self-efficacy for managing symptoms, pain interference, fatigue, and physical function)
4	Measured periodically through the SW-CRT design from all eligible subjects.	MyChart activation (binary outcome)
		MyChart logins per patient per month

6.2. Data Analysis

<u>**Analysis of Outcomes classified as Type 1:**</u>

- For descriptive purposes, we will estimate crude means for continuous outcomes and crude proportions for dichotomous outcomes and calculate corresponding 95% confidence intervals by site. The coefficient of variation is calculated using the site-specific point estimates.
- Next, we estimate the overall mean (or proportion). To this end, a weighted mean and corresponding 95% confidence interval will be calculated using the site-specific point estimates and their standard error estimates, using the inverse of the site-specific variance estimates for the weights.
- In addition, generalized linear models (GLMs) will be used to investigate factors associated with the outcome. In the GLMs, we will account for site variability by treating "site" as a fixed effect. For modeling continuous outcomes, we will use the identity link function. For dichotomous outcomes, the logit link function is used.

<u>**Analysis of Outcomes classified as Type 2:**</u>

- We will calculate the site-period-specific means (or proportions), adjusted for the factors potentially associated with the outcome. We will calculate the coefficient of variations across sites and also across periods, respectively.
- To this end, we will use generalized linear mixed-effects models (GLMMs). In GLMMs, we will account for site variability by treating "site" as a fixed-effect, secular trend by treating "period" as a fixed-effect, and within-subject variability by treating "subject" as random-effects. For modeling continuous outcomes, we will employ the identity link function. For

dichotomous outcomes, the logit link function will be utilized. G-computation will be used to derive the site-period-specific means (or proportions).

- We will also calculate the site-specific, period-specific, and overall means (or proportions) and corresponding 0.95 confidence interval by taking a weighted average of the site-period-specific mean values (or proportions) obtained through GLMMs. The reciprocal of the variance-covariance matrices of the site-period-specific mean values (or proportions) will be used as the weight.

<u>Analysis of Outcomes classified as Type 3:</u>

- For descriptive purposes, we will estimate crude means for continuous outcomes and proportions for dichotomous outcomes and calculate corresponding 95% confidence intervals by intervention group (eSyM+ vs. eSyM-) and site.
- Using these results, the difference between the intervention condition (eSyM+) and the control condition (eSyM-) along with a corresponding 95% confidence interval will be calculated for each site. In addition, using the point estimates from the six sites, we will calculate the coefficient of variation of the difference between conditions.
- We will then estimate the overall between-conditions difference integrating the site-specific differences. Specifically, a weighted average and corresponding 95% confidence interval will be calculated, using the site-specific point estimates their standard error estimates, where the reciprocal of the site-specific variance estimates will be used for the weights.
- In addition, generalized linear models (GLMs) will be used to investigate factors associated with the outcome. In the GLMs, we will include the intervention indicator, “site” and other factors (i.e., age (at cancer diagnosis for medical oncology patients and at index surgery for surgical patients), sex, employment status, education, ability to pay bills, and technology confidence) as independent variables. For modeling continuous outcomes, we will employ the identity link function. For dichotomous outcomes, the logit link function will be utilized. Note that, when more appropriate (where some subjects have data from both pre- and post-intervention periods), we will use GLMMs by treating “subject” as random-effects instead of GLMs for some outcomes.

<u>Analysis of Outcomes classified as Type 4:</u>

- We will estimate the intervention effect on each outcome.
- To this end, analyses similar to those for Aim 2a (see Section 4) will be conducted. GLMMs will be used with the identity link function for continuous outcomes and with the logit link function for dichotomous outcomes. The intervention effect will be summarized as the mean difference and odds ratio for continuous outcomes and dichotomous outcomes, respectively, and reported with a corresponding 95% confidence interval and p-value.

<u>Interpretation of the PROMIS scores:</u> Note that Yost and Cella have reported minimally important difference (MID) ranges for five PROMIS domains including fatigue, pain, depression, anxiety, and physical functioning[17,18] Cella recommends using 0.5 SD as the MID for PROMIS scales[19,20]

<u>Approach to missing data for PROMIS scores:</u> Because a random missing mechanism assumption is not verifiable, we will use several methods to handle missing observations. Specifically, we will perform: (1) mean value; (2) worst-case; (3) best-case; and, (4) multiple imputations.[13]

7. Analysis for Aim 2d: Patients' satisfaction with their cancer care

7.1. Outcomes and summary measures

The questionnaire asks patients to report on satisfaction with their cancer care using the CAHPS Cancer Care Survey. This outcome is classified as the Type 3 outcome in Table 12.

7.2. Data Analysis

Aim 2d survey methods and sampling mirrors Aim 2c (PROMIS items). Following Section 6.2 (Analysis of Outcomes classified as Type 3), we will perform the analysis for these continuous outcomes. We will use the AHRQ's CAHPS Analysis Program.[21,22]

8. Analysis for Aim 3a: Patient adoption, clinician utilization, and their perspectives on appropriateness and acceptability

8.1. Outcomes and summary measures

The outcomes investigated in Aim 3a in the SIMPRO study are classified into four groups, depending on the data source and data collection schedule. **Table 13** shows the details of the groups and the disposition of each outcome in Aim 3a.

Table 13: Outcomes in Aim 3a

Type	Outcome Type in the SIMPRO study	Example outcomes in Aim 3a
1	Measured at one single time point only either in post-intervention (the intervention condition) or in pre-intervention (the control condition) for all or selected subjects (patients or providers)	Qualitative feedback
		Pt satisfication with eSyM tool
		System Usability Items
		FIM item
		AIM items (acceptability)
		NOMAD items
		CSAT items
		# of med onc clinics continuing to use eSyM after stepped wedge period
		# of surg clinics continuing to use eSyM after stepped wedge period
		# of clinics that have >= 1 team conducting eSyM severe symptom outreach (e.g. using IB alerts)
		# of clinics that have >= 1 team conducting eSyM population symptom mgmt (e.g. using reports)
		Clicker questions (readiness + appropriateness for intervention) **[pre-intervention only]**
2	Measured at multiple time points (periodically) only in post-intervention period for all or selected subjects (patients or providers)	% of patients eligible to use eSyM completing 1+ qnr (PRIMARY IMPLEMENTATION OUTCOME)
		# pts assigned to eSyM/total # of pts with index condition on eSyM registry
		# reporting eSyM once, 25%, 50%, 75% of prompts
		Clinical staff responses to eSyM reports (InBasket + Telephone Encounters + Messages)
		Patient eSyM Total Usage Rate (w/MyChart) - up to 1 year after go-live
		Patient eSyM Total Usage Rate (w/ or w/o MyChart) - up to 1 year after go-live
		Patient eSyM Weekly Usage Rate (w/MyChart) - up to 1 year after go-live
		Patient eSyM Weekly Usage Rate (w/ or w/o MyChart) - up to 1 year after go-live
		% of MyChart Patients w/ documented outreach - up to 1 year after go-live
		% of patients with eSyM assigned w/ documented outreach - up to 1 year after go-live
		% of weekly patients responding to eSyM w/ documented outreach - up to 1 year after go-live
		% of weekly responders reporting moderate-severe symptoms - up to 1 year after go-live
		% of weekly responders reporting severe symptoms - up to 1 year after go-live

3	Measured from selected subjects (patients or providers) at a single time point, which could be during the pre-intervention period or the post-intervention period. For some subjects, data may be collected at both periods.	program barriers (stakeholder survery)
		Program facilitators (stakeholder survery)
4	Measured periodically through the SW-CRT design from all eligible subjects.	# of telephone encounter per patient per month

Notes:

- Both patient adoption and clinician utilization can be observed from analyzing EHR data based on eSyM utilization patterns. Clinician utilization can also be measured from the EHR and will be grouped in categories.

- Data for Aim 3a (clinicians) will be collected from the EHR based on system utilization patterns as well as from qualitative and quantitative survey, especially interviews with participating staff and clinicians.

- Appropriateness and acceptability will be ascertained using Weiner's AIM surveys (8-items total) which will be administered along with CAHPS surveys. Appropriateness and acceptability ratings will be defined based on the % of respondents who "agree" or "completely agree" with the survey items compared to the % who are neutral, disagree, or completely disagree and characterized using descriptive statistics.[23]

8.2. Data Analysis

Analysis of Outcomes classified as Type 1: See Section 6.2

Analysis of Outcomes classified as Type 2: See Section 6.2

Analysis of Outcomes classified as Type 3: See Section 6.2

Analysis of Outcomes classified as Type 4: See Section 6.2

9. Analysis for Aim 3b: The sustainability of ePRO symptom management within a health system

Hypotheses:
(1) We hypothesize that 3 or more of our health systems will continue eSyM reporting for MO patients beyond 90 days.
(2) We hypothesize that tapering the dedicated nursing support provided by the study does not affect the effect of eSyM on clinical/utilization outcomes.

Analysis:

We will evaluate sustainability at the patient, clinic and health system level using simple rates and proportions. To evaluate sustainability, we will examine the consequences of withdrawing grant-funded nursing support for symptom management in the post-implementation period. We will compare outcomes from Period 6 (study month 45-50, all sites eSyM+) and the post-Implementation (Post-I; study months 51-56). Sites are trained and empowered to manage eSyM autonomously without research study staff. Then, during post-implementation, dedicated nursing support to monitor eSyM is *tapered* in half the sites (see Figure C2). To examine whether backing off on the study support attenuates the effect, we will perform difference in difference analysis. For each outcome, we will calculate the difference between Post-I and Period 6 outcomes by site. We then calculate the difference between site groups and the corresponding 95% confidence intervals.

10. Analysis for Aim 3c (Penetration and scalability of ePROs for symptom management) and Aim 3d (Extent of adaptation of ePRO systems over the course of the implementation process)

<u>A mixed-methods approach:</u>
Our analytic approach will integrate qualitative and quantitative data to obtain an informative description of factors that influence implementation at the level of 1) the practice site, 2) the health system, and 3) the entire project. The CFIR schema will facilitate comparisons, across settings and timepoints. First, we will analyze the qualitative and quantitative data separately. Next, we will use a weaving approach to integrate each source narrative to report findings on a theme-by-theme basis.[24]

<u>Qualitative:</u>
Analysis of interviews and group discussions will rely on notes and transcribed audiotapes entered in NVivo software. Given the study purpose, we will use a framework analysis[25–27] approach that allows for systematic analysis that is also flexible and iterative in nature.[28] Through indexing, charting, and mapping we will be able to draw comparisons across interviews to identify facilitators and barriers. Lastly, we will use the CFIR qualitative data scoring schema **(Table 14)** to assign a numeric code ranging from -2 to +2 to each construct summarizing whether it was a negative or positive influence on eSyM use. The weighted kappa statistic, with 0.70 as the cut-off will be used to assess agreement between coders. Ratings will be used to make topographic maps to visually convey the relative importance of each CFIR construct.[29]

Table 14: Rating the Influence of CFIR Constructs on Implementation Outcomes

-2	Negative influence, impeding influence in implementation efforts
-1	Negative influence, impeding influence in implementation efforts (general impression but no concrete examples given during interviews; mixed effect)
0	Neutral influence; contradictory interviews
+1	Positive influence, facilitating influence in implementation efforts (general statements but no concrete examples; mixed effect, but generally positive)
+2	Positive influence, facilitating influence (firm examples shown or give)
missing	Lack of interviewee input or absence of evaluable construct

<u>Quantitative:</u>
Clinician surveys will use ordinal response scales to capture the valence (+/- influence) for each CFIR construct and simple descriptive statistics to characterize responses. Finally, variables organized by CFIR domain will be combined with qualitative data on a theme-by-theme basis. This will provide an interpretable numeric summary of the perceived importance of each construct. We will use 95%

confidence intervals as a measure of precision. All analyses at the health system level will adjust for clustering using generalized linear mixed effects models.

11. References

1. Curran GM, Bauer M, Mittman B, Pyne JM, Stetler C. Effectiveness-implementation hybrid designs: combining elements of clinical effectiveness and implementation research to enhance public health impact. Med Care. 2012 Mar;50(3):217–226. PMCID: PMC3731143

2. Loudon K, Treweek S, Sullivan F, Donnan P, Thorpe KE, Zwarenstein M. The PRECIS-2 tool: designing trials that are fit for purpose. BMJ. BMJ; 2015 May 8;350(may08 1):h2147. PMID: 25956159

3. Möhler R, Köpke S, Meyer G. Criteria for Reporting the Development and Evaluation of Complex Interventions in healthcare: revised guideline (CReDECI 2). Trials. Springer Science and Business Media LLC; 2015 May 3;16(1):204. PMCID: PMC4461976

4. Calvert M, Blazeby J, Altman DG, Revicki DA, Moher D, Brundage MD, for the CONSORT PRO Group. Reporting of Patient-Reported Outcomes in Randomized Trials [Internet]. JAMA. 2013. p. 814. Available from: http://dx.doi.org/10.1001/jama.2013.879

5. Campbell MK, Piaggio G, Elbourne DR, Altman DG, for the CONSORT Group. Consort 2010 statement: extension to cluster randomised trials. BMJ. British Medical Journal Publishing Group; 2012 Sep 4;345(sep04 1):e5661–e5661.

6. Grayling MJ, Wason JMS, Mander AP. Stepped wedge cluster randomized controlled trial designs: a review of reporting quality and design features. Trials. 2017 Jan 21;18(1):33. PMCID: PMC5251280

7. Hemming K, Taljaard M, McKenzie JE, Hooper R, Copas A, Thompson JA, Dixon-Woods M, Aldcroft A, Doussau A, Grayling M, Kristunas C, Goldstein CE, Campbell MK, Girling A, Eldridge S, Campbell MJ, Lilford RJ, Weijer C, Forbes AB, Grimshaw JM. Reporting of stepped wedge cluster randomised trials: extension of the CONSORT 2010 statement with explanation and elaboration. BMJ. 2018 Nov 9;363:k1614. PMCID: PMC6225589

8. Hemming K, Taljaard M, Grimshaw J. Introducing the new CONSORT extension for stepped-wedge cluster randomised trials [Internet]. Trials. 2019. Available from: http://dx.doi.org/10.1186/s13063-018-3116-3

9. Ariti C. Walter W Stroup, Generalized linear mixed models, modern concepts, methods and applications. Stroup Walter W , Generalized linear mixed models, modern concepts, methods and applications . CRC Press: Boca Raton, 2012; £59.99 p. 555. Stat Methods Med Res. SAGE Publications; 2017 Apr;26(2):1043–1044. PMID: 25549967

10. Elff M, Heisig JP, Schaeffer M, Shikano S. Multilevel analysis with few clusters: Improving likelihood-based methods to provide unbiased estimates and accurate inference. Br J Polit Sci. Cambridge University Press (CUP); 2021 Jan;51(1):412–426.

11. Kenward MG, Roger JH. Small sample inference for fixed effects from restricted maximum likelihood. Biometrics. 1997 Sep;53(3):983–997. PMID: 9333350

12. Li P, Redden DT. Comparing denominator degrees of freedom approximations for the generalized linear mixed model in analyzing binary outcome in small sample cluster-randomized trials. BMC Med Res Methodol. Springer Nature; 2015 Apr 23;15(1):38. PMCID: PMC4458010

13. Schafer JL. Multiple imputation: a primer. Stat Methods Med Res. SAGE PublicationsSage UK: London, England; 1999 Mar 1;8(1):3–15.

14. Hemming K, Taljaard M. Sample size calculations for stepped wedge and cluster randomised trials: a unified approach. J Clin Epidemiol. Elsevier Inc; 2016 Jan 1;69:137–146.

15. Campbell MK, Mollison J, Grimshaw JM. Cluster trials in implementation research: estimation of intracluster correlation coefficients and sample size. Stat Med. 2001 Feb 15;20(3):391–399. PMID: 11180309

16. Eldridge SM, Costelloe CE, Kahan BC, Lancaster GA, Kerry SM. How big should the pilot study for my cluster randomised trial be? Stat Methods Med Res. SAGE Publications Ltd STM; 2016 Jun 1;25(3):1039–1056.

17. Yost KJ, Eton DT, Garcia SF, Cella D. Minimally important differences were estimated for six Patient-Reported Outcomes Measurement Information System-Cancer scales in advanced-stage cancer patients [Internet]. Journal of Clinical Epidemiology. 2011. p. 507–516. Available from: http://dx.doi.org/10.1016/j.jclinepi.2010.11.018

18. Chambers DA, Glasgow RE, Stange KC. The dynamic sustainability framework: addressing the paradox of sustainment amid ongoing change. Implement Sci. 2013 Oct 2;8:117. PMCID: PMC3852739

19. Temel JS, Greer JA, El-Jawahri A, Pirl WF, Park ER, Jackson VA, Back AL, Kamdar M, Jacobsen J, Chittenden EH, Rinaldi SP, Gallagher ER, Eusebio JR, Li Z, Muzikansky A, Ryan DP. Effects of Early Integrated Palliative Care in Patients With Lung and GI Cancer: A Randomized Clinical Trial. J Clin Oncol. 2017 Mar 10;35(8):834–841. PMCID: PMC5455686

20. Chen CX, Kroenke K, Stump TE, Kean J, Carpenter JS, Krebs EE, Bair MJ, Damush TM, Monahan PO. Estimating minimally important differences for the PROMIS pain interference scales: results from 3 randomized clinical trials. Pain. 2018 Apr;159(4):775–782. PMCID: PMC5860950

21. Hays R, Quigley D, Mendel P, Predmore Z, Chen AY. Use of CAHPS® patient experience survey data as part of a patient-centered medical home quality improvement initiative [Internet]. Journal of Healthcare Leadership. 2015. p. 41. Available from: http://dx.doi.org/10.2147/jhl.s69963

22. Quigley D, Qureshi N, Rybowski L, Shaller D, Edgman-Levitan S, Cleary PD, Ginsberg C, Hays RD. Summary of the 2020 AHRQ research meeting on ‘advancing methods of implementing and evaluating patient experience improvement using consumer assessment of healthcare providers and systems (CAHPS®) surveys’ [Internet]. Expert Review of Pharmacoeconomics & Outcomes Research. 2022. p. 883–890. Available from: http://dx.doi.org/10.1080/14737167.2022.2064848

23. Basch E, Snyder C. Overcoming barriers to integrating patient-reported outcomes in clinical practice and electronic health records. Annals of oncology: official journal of the European Society for Medical Oncology / ESMO. 2017. p. 2332–2333. PMID: 28961852

24. Snyder CF, Herman JM, White SM, Luber BS, Blackford AL, Carducci MA, Wu AW. When using patient-reported outcomes in clinical practice, the measure matters: a randomized controlled trial. J Oncol Pract. 2014 Sep;10(5):e299-306. PMCID: PMC4161731

25. Roess A. The Promise, Growth, and Reality of Mobile Health - Another Data-free Zone. N Engl J Med. 2017 Nov 23;377(21):2010–2011. PMID: 29116869

26. Jensen RE, Snyder CF. PRO-cision Medicine: Personalizing Patient Care Using Patient-Reported Outcomes. Journal of clinical oncology: official journal of the American Society of Clinical Oncology. 2016. p. 527–529. PMID: 26644538

27. Hughes EF, Wu AW, Carducci MA, Snyder CF. What can I do? Recommendations for responding to issues identified by patient-reported outcomes assessments used in clinical practice. J Support Oncol. 2012 May 18;10(4):143–148. PMCID: PMC3384764

28. Cortez NG, Cohen IG, Kesselheim AS. FDA regulation of mobile health technologies. N Engl J Med. 2014 Jul 24;371(4):372–379. PMID: 25054722

29. Brundage M, Blackford A, Tolbert E, Smith K, Bantug E, Snyder C, PRO Data Presentation Stakeholder Advisory Board (various names and locations). Presenting comparative study PRO results to clinicians and researchers: beyond the eye of the beholder. Qual Life Res. 2018 Jan;27(1):75–90. PMCID: PMC5770492

ADDENDUM TO THE STATISTICAL ANALYSIS PLAN

for

PROTOCOL ACTIVITY 4

Pragmatic stepped-wedge cluster randomized trial

SIMPRO Research Center: Integration and Implementation of PROs for Symptom Management in Oncology Practice.

DATE: *June 12, 2024*

Sponsor and Coordinating Center:

Dana-Farber/Harvard Cancer Center (DF/HCC)
Department of Medical Oncology
Coordinating Center PI and Technology PI: Michael Hassett
Dana-Farber Cancer Institute
Boston, MA 02215
Email Address: Michael_Hassett@dfci.harvard.edu
Telephone Number: 617-632-4587

CO-STUDY CHAIRS:

Deborah Schrag MD, MPH
Chair, Department of Medicine
Memorial Sloan Kettering Cancer Center
New York City, NY 10065

Raymond Osarogiagbon MD
Director, Thoracic Oncology Research Group
Baptist Memorial Hospital
Covington, Tennessee 38019

Sandra Wong MD
Chair, Department of Surgery
Dartmouth College
Lebanon, NH 03756

STUDY STATISTICIAN:

Hajime Uno, PhD
Dana-Farber Cancer Institute
Boston, MA 02215

FUNDING:

National Cancer Institute, UM1CA233080.

SIGNATURE PAGE

Protocol Title:	SIMPRO Research Center: Integration and Implementation of PROs for Symptom Management in Oncology Practice.
Sponsor Protocol Number:	DF/HCC IRB #18-986R/18-734
sIRB Protocol Number:	WIRB Tracking #20182593; Study #1248093
SAP Version:	Addendum to Version 2.0
Date:	June 12, 2024

Michael Hassett, MD, MPH<br>Study Chair, Coordinating Center PI	Date
Deborah Schrag, MD, MPH<br>Co-Chair, Co-PI	Date
Raymond Osarogiagbon, MD<br>Co-Chair, Co-PI	Date
Sandra Wong, MD<br>Co-Chair, Co-PI	Date
Hajime Uno, PhD<br>Study Statistician	Date

Introduction

This addendum outlines modifications to the Statistical Analysis Plan (SAP) version 2.0 (January 9, 2024) for the pragmatic stepped-wedge cluster randomized trial at SIMPRO Research Center due to challenges encountered in the original analysis plan.

In this study, follow-up for clinical outcomes for each patient starts from the occurrence of a trigger event (i.e., initiation of a new chemotherapy for Medical Oncology and discharge after receiving a qualifying surgical procedure for Surgery). Therefore, we potentially have more than one data point (i.e., episode) from one subject in this study.

The primary analysis has been adjusted from including all episodes observed from each patient during the study period (see Pages 12 of the SAP ver. 2.0) to including only the first episode observed from each patient. Accordingly, the primary analysis has been changed from including subjects as random effects in generalized linear mixed effects models to take account of within-subject correlation (see Page 17 on the SAP ver. 2.0) to using genialized linear models because the analysis data will not involve within-subject correlation.

This change is necessitated by the observation that the majority of patients did not experience multiple episodes, leading to convergence issues with the generalized linear mixed-effects models initially planned as the primary analysis. Especially in Surgery cohort, only 2.8% subjects had multiple episodes under the intervention condition.

Changes to the Statistical Analysis Plan

1. Multiple episodes for medical record data (page 12)

Original:
- The primary analysis will include all these episodes other than the contaminated episodes.
- As sensitivity analyses, we will perform the analyses using the data consisting of only the first episode from each subject.

Revised:
- The primary analysis will include only the first episode from each subject.
- As sensitivity analyses, we will perform the analyses include all episodes other than the contaminated episodes.

2. Analysis Populations (page 15)

Original:
- 1) The primary analysis population consists of all eligible episodes under the intervention condition (A+B) (i.e., eSyM exposed) and all eligible episodes under the control condition (C) (i.e., eSyM unexposed).

- 2) The secondary analysis population consists of the episodes where patients engaged with eSyM (A) and the matched episodes during the pre-intervention period (C*).

Revised:

- 1) The primary analysis population consists of all 1st eligible episodes under the intervention condition (A+B) (i.e., eSyM exposed) and all 1st eligible episodes under the control condition (C) (i.e., eSyM unexposed).
- 2) The secondary analysis population consists of the 1st episodes where patients engaged with eSyM (A) and the matched 1st episodes during the pre-intervention period (C*).

<u>3. Patient demographic and disease characteristics (Page 16)</u>

Original:

- The unit of the analysis is not patient but episode. All eligible episodes eSyM- episodes will be included in this analysis.

Revised:

- The unit of the analysis is patient in the primary analysis. All eligible 1st episodes will be included in this analysis.

<u>4. Multilevel Generalized Linear Regression Analysis (Page 16)</u>

Original:

- The primary analysis for the outcomes in Aim 2 (Table 5) will be performed via multilevel generalized linear regression models (or generalized linear mixed-effects model; GLMM).
- Subjects
  - We will potentially have more than one data point from one subject in this study.
  - To take account of within-subject correlation, subjects will be included as random effects.
  - The primary analysis will consider random intercepts only.

Revised:

- The primary analysis for the outcomes in Aim 2 (Table 5) will be performed via generalized linear regression models (or generalized linear model; GLIM).

- Subjects
  - We will potentially have more than one episode from one subject in this study. However, only the 1st episode from each patient will be included in the primary analysis.
  - If possible, as a sensitivity analysis, we will perform multilevel generalized linear regression models (or generalized linear mixed-effects model; GLMM) by including all episodes. To take account of within-subject correlation, subjects will be included as random effects.

<u>5. Sensitivity analyses (Page 19):</u>

Original:

- Analysis with data from the first episode
    - In the primary analysis, all episodes in the analyses except for eSyM- episodes contaminated with the eSyM intervention will be included. We will perform sensitivity analyses using only the first episode from each patient. In these sensitivity analyses, each patient will serve only one data point in the analysis.

Revised:

- Analysis with data from all episodes
    - In the primary analysis, only the first episode from each patient will be used. In the primary analysis, each patient will serve only one data point in the analysis. We will perform sensitivity analyses using all episodes in the analyses except for eSyM- episodes contaminated with the eSyM intervention will be included.

Implementation and Documentation

These changes will be implemented immediately and documented in all relevant study documents and communications. The SAP addendum will be reviewed and approved by the IRB. All analyses will be conducted based on this SAP addendum.
