# Supplementary material for: Longitudinal Patterns of Symptoms in Patients Undergoing Chemotherapy: A Secondary Analysis of a Cluster Randomized Clinical Trial
Source: JAMA Netw Open. 2026 Apr 6;9(4):e264996. doi: 10.1001/jamanetworkopen.2026.4996 (PMC13054620; doi:10.1001/jamanetworkopen.2026.4996)
Supplement: Supplement 2. — eFigure 1. An Illustration of Start and Stop Counting Process for Time-Varying Cox Proportional Hazards Model eTable. Frequencies and Percentages of Symptom Alerts and Composite Scores by Week eFigure 2. Distribution of 18 Optional Symptoms by Scores** eFigure 3. Symptom Scores Stratified by Cancer Type eFigure 4. Unique Co-Occurrences of Moderate to Severe Symptoms Across All Time Points eFigure 5. CONSORT SIMPRO eFigure 6. Distribution of 12 Required Symptoms, Physical Function and Overall Wellbeing by Severity for Patients with GYN Cancer (N=9474 Surveys) eFigure 7. Distribution of 12 Required Symptoms by Severity for Patients with Thoracic Cancer (N=9967 Surveys) [file jamanetwopen-e264996-s002.pdf]

Supplemental Online Content

Paudel R, Wright AA, Cronin C, et al. Longitudinal patterns of symptoms in patients undergoing chemotherapy. *JAMA Netw Open*. 2026;9(4):e264996. doi:10.1001/jamanetworkopen.2026.4996

eFigure 1. An Illustration of Start and Stop Counting Process for Time-Varying Cox Proportional Hazards Model
eTable. Frequencies and Percentages of Symptom Alerts and Composite Scores by Week
eFigure 2. Distribution of 18 Optional Symptoms by Scores**
eFigure 3. Symptom Scores Stratified by Cancer Type
eFigure 4. Unique Co-Occurrences of Moderate to Severe Symptoms Across All Time Points
eFigure 5. CONSORT SIMPRO
eFigure 6. Distribution of 12 Required Symptoms, Physical Function and Overall Wellbeing by Severity for Patients with GYN Cancer (N=9474 Surveys)
eFigure 7. Distribution of 12 Required Symptoms by Severity for Patients with Thoracic Cancer (N=9967 Surveys)

This supplemental material has been provided by the authors to give readers additional information about their work.

© 2026 Paudel R et al. *JAMA Network Open.*

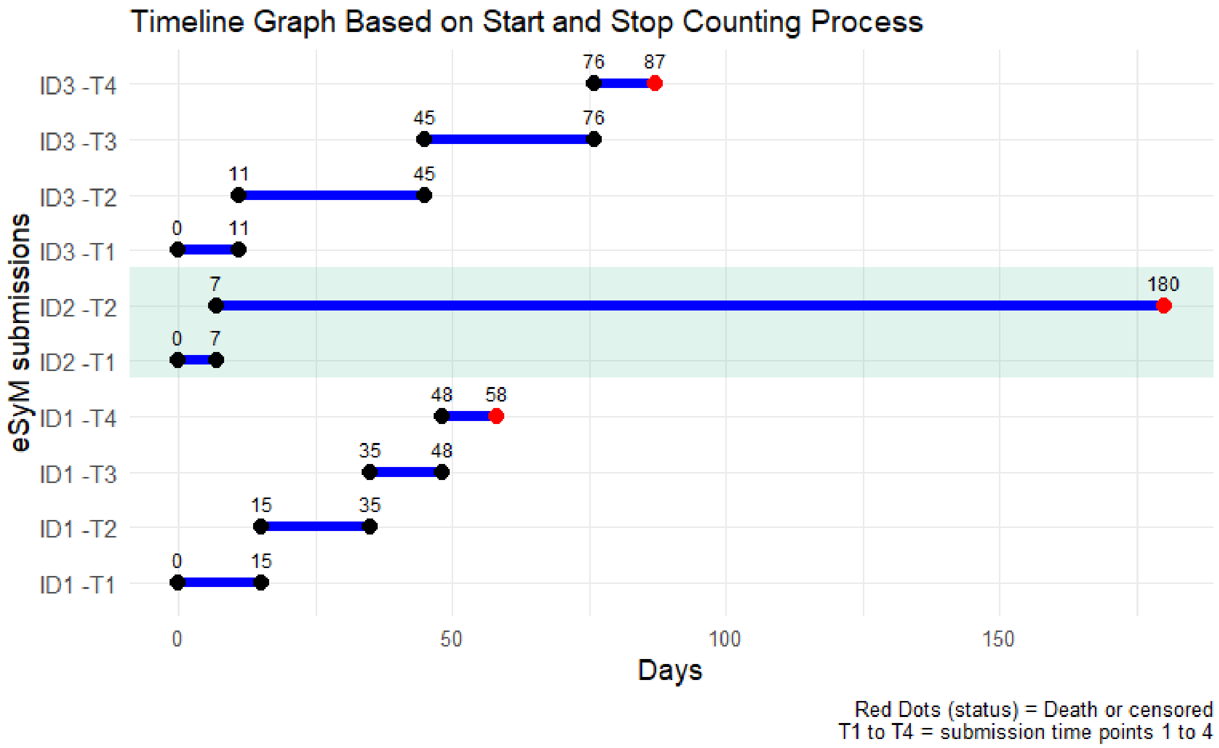

eFigure 1. An Illustration of Start and Stop Counting Process for Time-Varying Cox Proportional Hazards Model

© 2026 Paudel R et al. *JAMA Network Open.*

eTable 1. Frequencies and Percentages of Symptom Alerts and Composite Scores by Week

Week	Patients	Surveys Completed	Symptom Alerts[a]	Total Symptoms Reported	None	Mild	Moderate	Severe
1	1,886	2,555	717 (28%)	30,660	17,367 (57%)	8,331 (27%)	3,522 (11%)	1,440 (5%)
2	1,484	2,092	473 (23%)	25,104	14,184 (57%)	7,130 (28%)	2,873 (11%)	917 (4%)
3	1,442	1,934	325 (17%)	23,208	13,718 (59%)	6,531 (28%)	2,322 (10%)	637 (3%)
4	1,305	1,679	299 (18%)	20,148	11,424 (57%)	5,919 (29%)	2,216 (11%)	589 (3%)
5	1,260	1,787	298 (17%)	21,444	12,396 (58%)	6,223 (29%)	2,277 (11%)	548 (3%)
6	1,285	1,849	247 (13%)	22,188	13,392 (60%)	6,198 (28%)	2,138 (10%)	460 (2%)
7	1,185	1,654	262 (16%)	19,848	11,600 (58%)	5,780 (29%)	2,010 (10%)	458 (2%)
8	1,058	1,314	194 (15%)	15,768	9,266 (59%)	4,595 (29%)	1,538 (10%)	369 (2%)
9	1,131	1,588	186 (12%)	19,056	11,616 (61%)	5,340 (28%)	1,745 (9%)	355 (2%)
10	1,095	1,561	188 (12%)	18,732	11,414 (61%)	5,220 (28%)	1,731 (9%)	367 (2%)
11	1,012	1,370	171 (12%)	16,440	10,038 (61%)	4,600 (28%)	1,515 (9%)	287 (2%)
12	948	1,208	129 (11%)	14,496	8,995 (62%)	3,991 (28%)	1,277 (9%)	233 (2%)
13	961	1,358	156 (11%)	16,296	10,081 (62%)	4,527 (28%)	1,422 (9%)	266 (2%)
14	909	1,306	146 (11%)	15,672	9,860 (63%)	4,256 (27%)	1,312 (8%)	244 (2%)
15	903	1,238	129 (10%)	14,856	9,329 (63%)	4,028 (27%)	1,275 (9%)	224 (2%)
16	807	1,022	100 (10%)	12,264	7,612 (62%)	3,438 (28%)	1,055 (9%)	159 (1%)
17	803	1,149	120 (10%)	13,788	8,748 (63%)	3,725 (27%)	1,118 (8%)	197 (1%)
18	831	1,204	117 (10%)	14,448	9,302 (64%)	3,787 (26%)	1,165 (8%)	194 (1%)
19	785	1,070	113 (11%)	12,840	8,218 (64%)	3,421 (27%)	1,013 (8%)	188 (1%)
20	741	928	107 (12%)	11,136	7,140 (64%)	2,897 (26%)	915 (8%)	184 (2%)
21	771	1,074	98 (9%)	12,888	8,354 (65%)	3,401 (26%)	984 (8%)	149 (1%)
22	752	1,069	106 (10%)	12,828	8,296 (65%)	3,319 (26%)	1,028 (8%)	185 (1%)
23	716	974	100 (10%)	11,688	7,613 (65%)	2,941 (25%)	969 (8%)	165 (1%)
24	676	841	90 (11%)	10,092	6,506 (64%)	2,655 (26%)	778 (8%)	153 (2%)
25	635	932	83 (9%)	11,184	7,257 (65%)	2,934 (26%)	851 (8%)	142 (1%)
26	275	294	24 (8%)	3,528	2,320 (66%)	903 (26%)	265 (8%)	40 (1%)

a. A single symptom alert is generated per survey if one or more severe symptoms are reported

© 2026 Paudel R et al. *JAMA Network Open.*

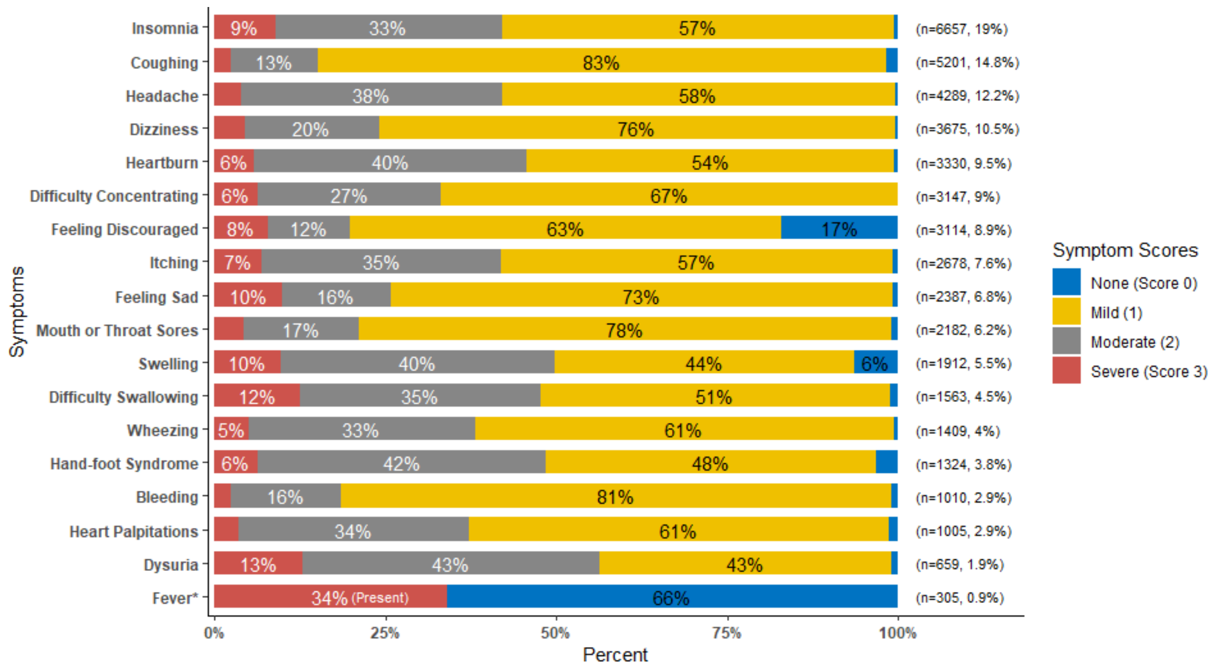

eFigure 2. Distribution of 18 Optional Symptoms by Scores**

**Fever is binary "Present" or "Absent"*

**Numbers and percentages in parentheses represent the total number of questionnaires with an optional symptom reported. For instance, 6,657 questionnaires included insomnia, which accounted for 19% of all questionnaires received.

© 2026 Paudel R et al. *JAMA Network Open.*

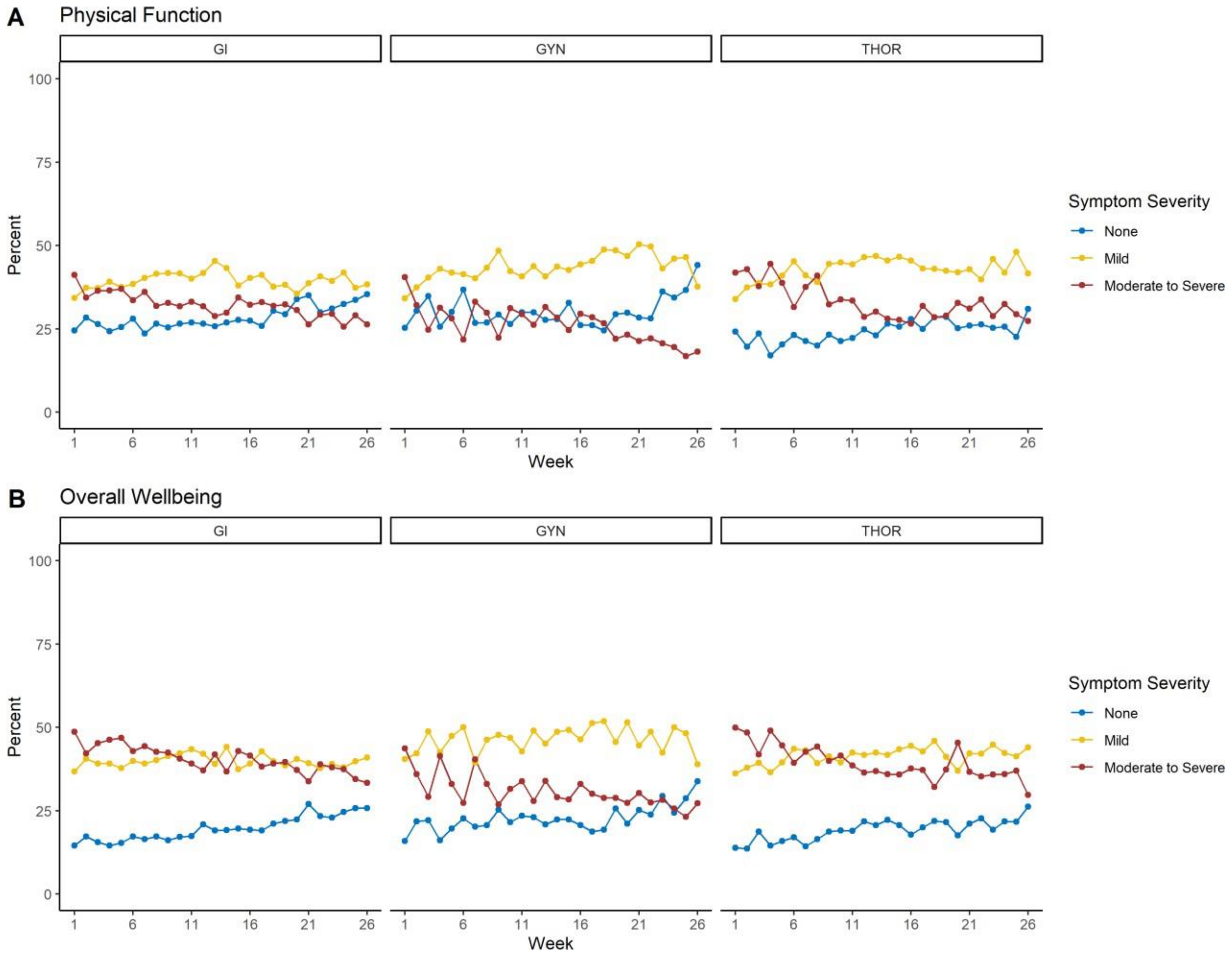

eFigure 3. Symptom Scores Stratified by Cancer Type

A-B. Physical Function and Overall Wellbeing Deficits Stratified by Cancer Type
GI: Gastrointestinal; GYN: Gynecologic, THOR: Thoracic.
Moderate or severe symptoms are collapsed into one category for visualization purposes

© 2026 Paudel R et al. *JAMA Network Open.*

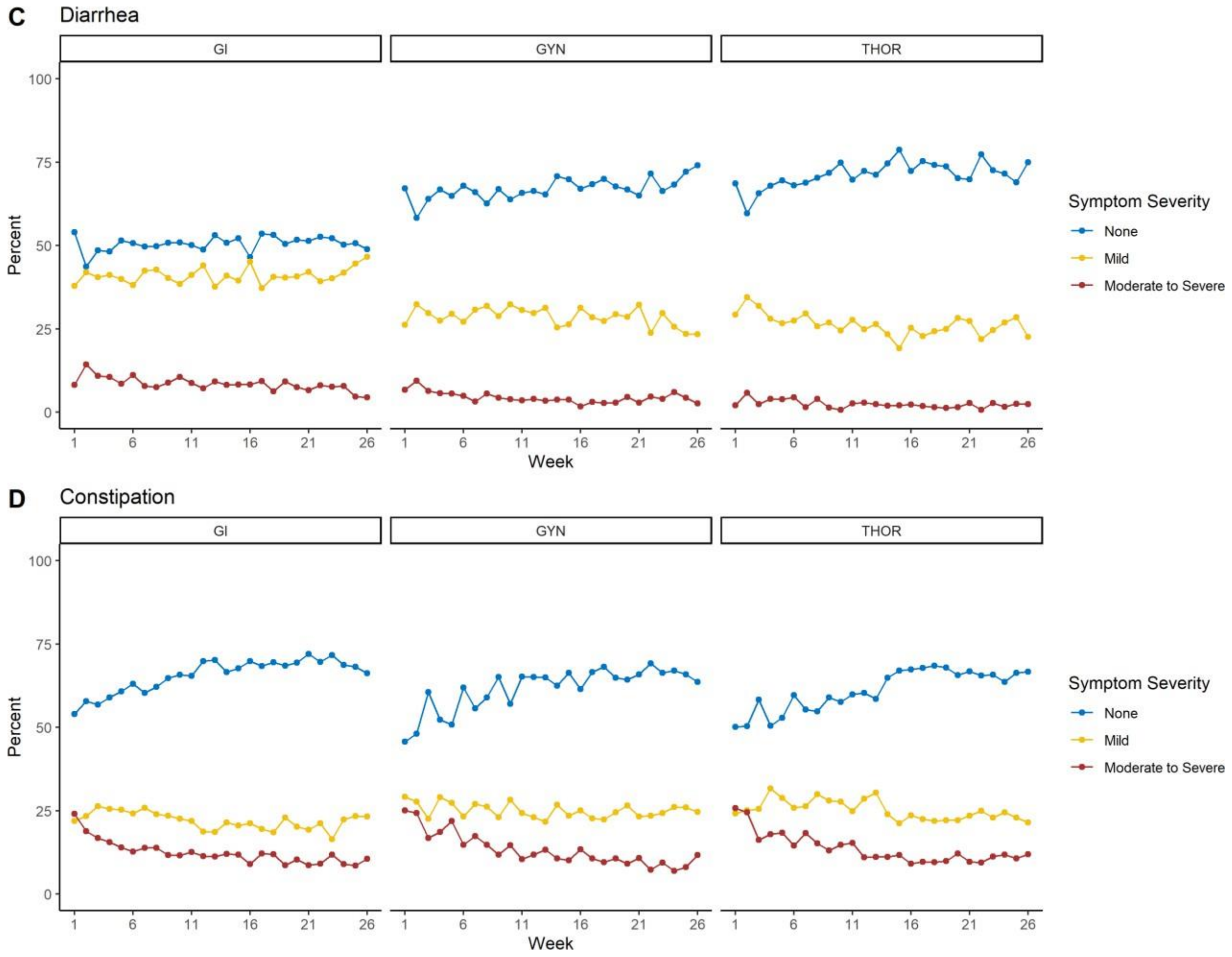

C-D. Moderate or severe symptoms are collapsed into one category for visualization purposes. GI: Gastrointestinal; GYN: Gynecologic, THOR: Thoracic.

© 2026 Paudel R et al. *JAMA Network Open.*

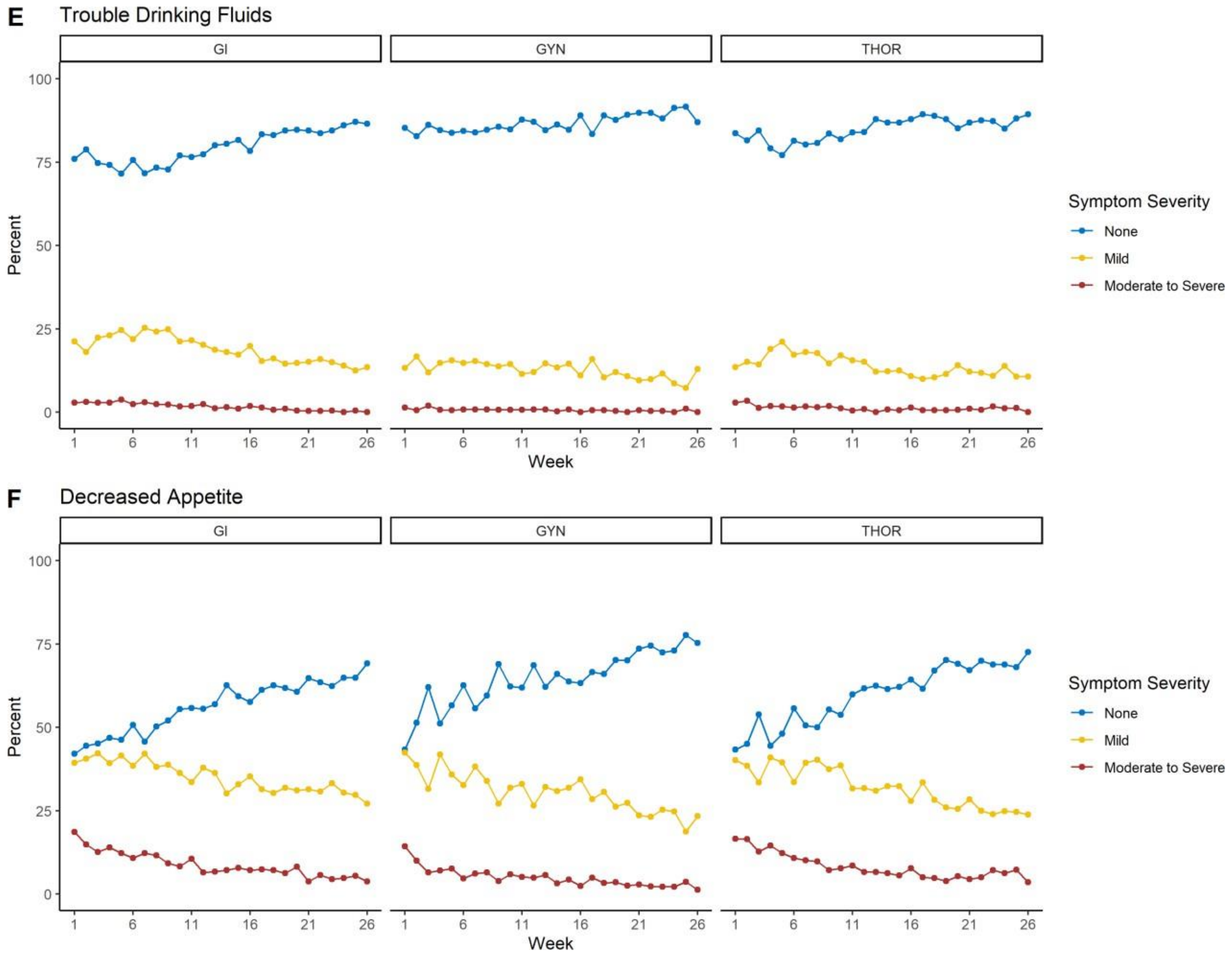

E-F. Trouble Drinking Fluids and Decreased Appetite Stratified by Cancer Type
GI: Gastrointestinal; GYN: Gynecologic, THOR: Thoracic.
Moderate or severe symptoms are collapsed into one category for visualization purposes

© 2026 Paudel R et al. *JAMA Network Open.*

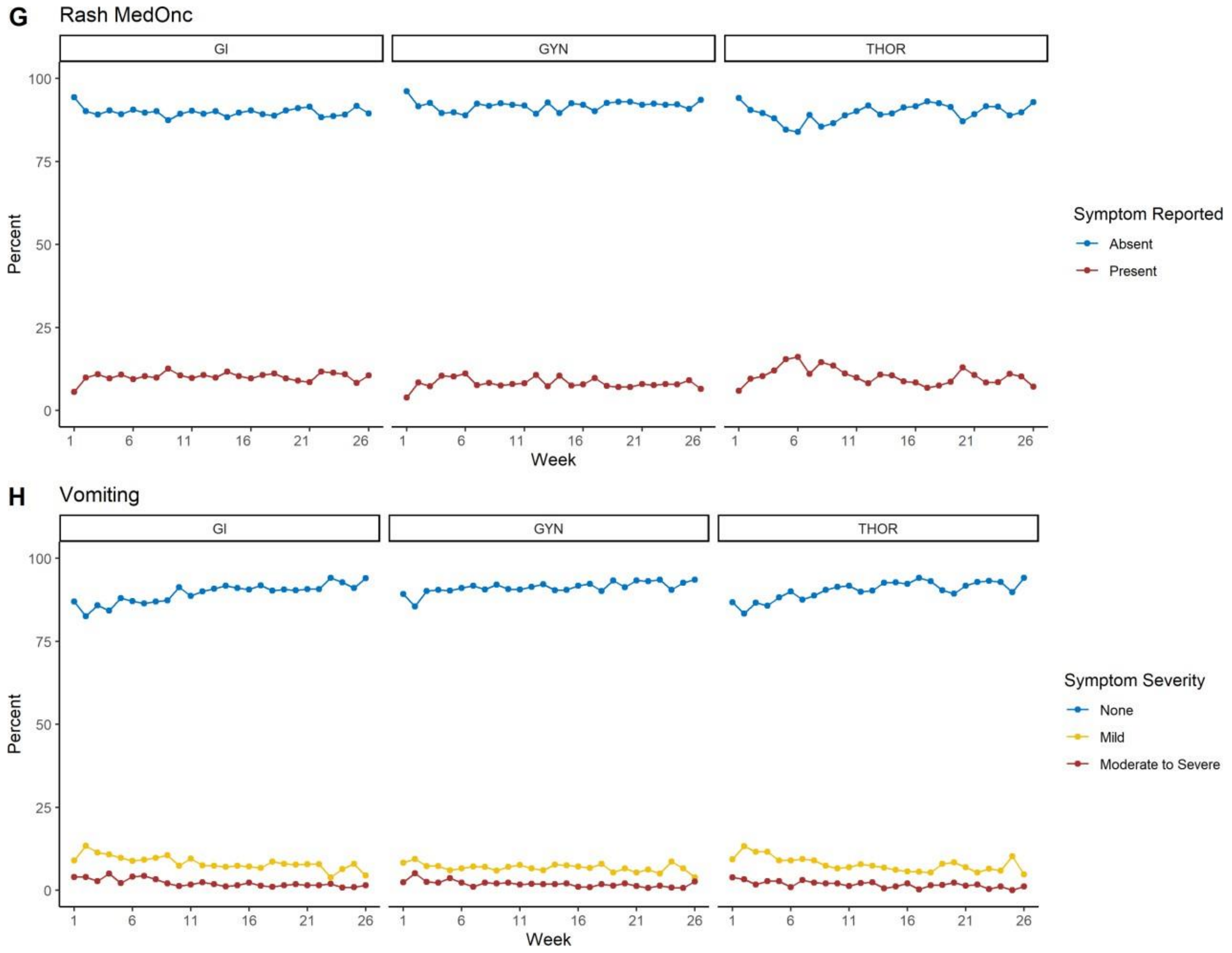

G-H. Rash and Vomiting by Cancer Type

GI: Gastrointestinal; GYN: Gynecologic, THOR: Thoracic.

Moderate or severe symptoms are collapsed into one category for visualization purposes

© 2026 Paudel R et al. *JAMA Network Open.*

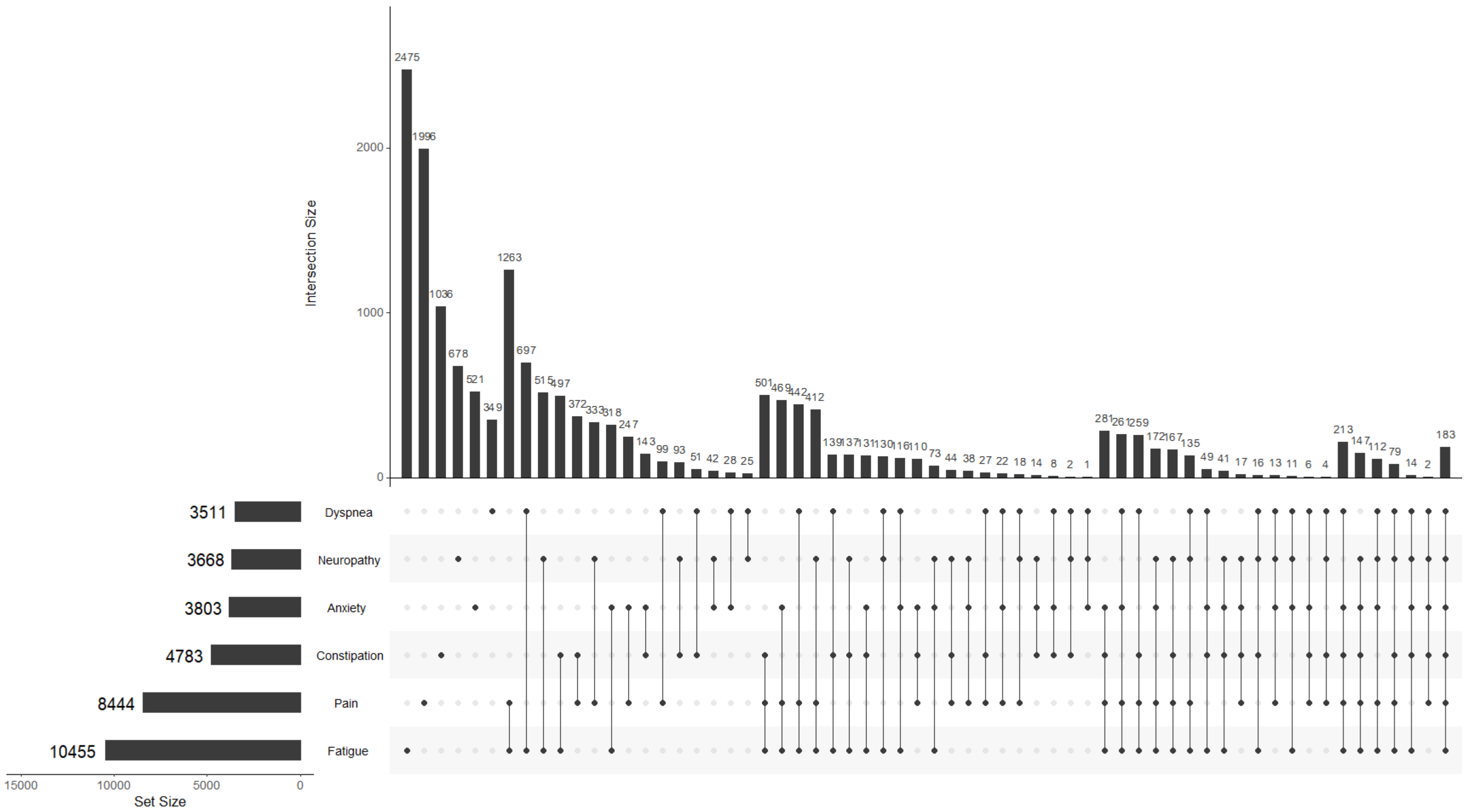

eFigure 4. Unique Co-Occurrences of Moderate to Severe Symptoms Across All Time Points.
Six most common symptoms are plotted on the x-axis. A black dot represents an instance of the symptom or the co-occurrence of more than one symptom. Conversely, a gray dot represents an exclusion, where a symptom does not occur or co-occur for that particular symptom or combination. Vertical lines represent the connection between the black dots, illustrating the unique co-occurrences. The horizontal bars (*set size*) represent the number of instances a moderate to severe symptom was reported (e.g., moderate to severe fatigue appeared in 10,455 questionnaires). The vertical bars (*intersection size*) represent the instances each symptom (e.g., moderate to severe fatigue appeared alone in 2,475 questionnaires) and co-occurrences (e.g., moderate to severe fatigue and pain co-occurred in 1,263 surveys) appeared. Similarly, moderate to severe fatigue, pain, and constipation appeared in 501 questionnaires. Lastly, all six moderate-to-severe symptoms were reported in 183 surveys.[23] The above plot visualizes the unique combinations of symptoms; therefore, each distinct set of co-occurring symptoms is counted only once.

© 2026 Paudel R et al. *JAMA Network Open.*

eFigure 5. CONSORT SIMPRO

	Period 1 (June 2018 – August 2019)	Period 2 (September 2019 - Feb 2020)	Period 3 (March 2020 – August 2020)	Period 4 (September 2020 – Feb 2021)	Period 5 (March 2021 – August 2021)	Period 6 (September 2021 – Feb 2022)	Period 7 (March 2022 - Feb 2023)
Sequence 1<br>Clusters allocated (N=1)<br>Baptist	MO control: 1075<br>MO intervention: 3	MO control: 18<br>MO intervention: 590	MO control: 0<br>MO intervention: 434	MO control: 0<br>MO intervention: 380	MO control: 0<br>MO intervention: 433	MO control: 0<br>MO intervention: 359	MO control: 0<br>MO intervention: 697
Sequence 2<br>Clusters allocated (N=1)<br>Maine	MO control: 321<br>MO intervention: 0	MO control: 213<br>MO intervention: 0	MO control: 11<br>MO intervention: 194	MO control: 0<br>MO intervention: 196	MO control: 0<br>MO intervention: 177	MO control: 0<br>MO intervention: 121	MO control: 0<br>MO intervention: 276
Sequence 3<br>Clusters allocated (N=1)<br>DFCI	MO control: 881<br>MO intervention: 0	MO control: 305<br>MO intervention: 0	MO control: 336<br>MO intervention: 0	MO control: 39<br>MO intervention: 300	MO control: 0<br>MO intervention: 349	MO control: 0<br>MO intervention: 332	MO control: 0<br>MO intervention: 768
Sequence 4<br>Clusters allocated (N=1)<br>Brown	MO control: 105<br>MO intervention: 0	MO control: 78<br>MO intervention: 0	MO control: 65<br>MO intervention: 0	MO control: 94<br>MO intervention: 0	MO control: 261<br>MO intervention: 0	MO control: 22<br>MO intervention: 277	MO control: 0<br>MO intervention: 544
Sequence 5<br>Clusters allocated (N=1)<br>DHMC	MO control: 134<br>MO intervention: 0	MO control: 54<br>MO intervention: 0	MO control: 59<br>MO intervention: 0	MO control: 62<br>MO intervention: 0	MO control: 126<br>MO intervention: 0	MO control: 83<br>MO intervention: 61	MO control: 0<br>MO intervention: 334
Sequence 6<br>Clusters allocated (N=1)<br>WVU	MO control: 303<br>MO intervention: 0	MO control: 100<br>MO intervention: 0	MO control: 64<br>MO intervention: 0	MO control: 95<br>MO intervention: 0	MO control: 106<br>MO intervention: 0	MO control: 115<br>MO intervention: 0	MO control: 25<br>MO intervention: 205

Total Patients = 39942
Medical Oncology Patients = 12180
Patients in Intervention = 7030
Patients in Control Condition = 5150
MO: Medical Oncology

© 2026 Paudel R et al. *JAMA Network Open.*

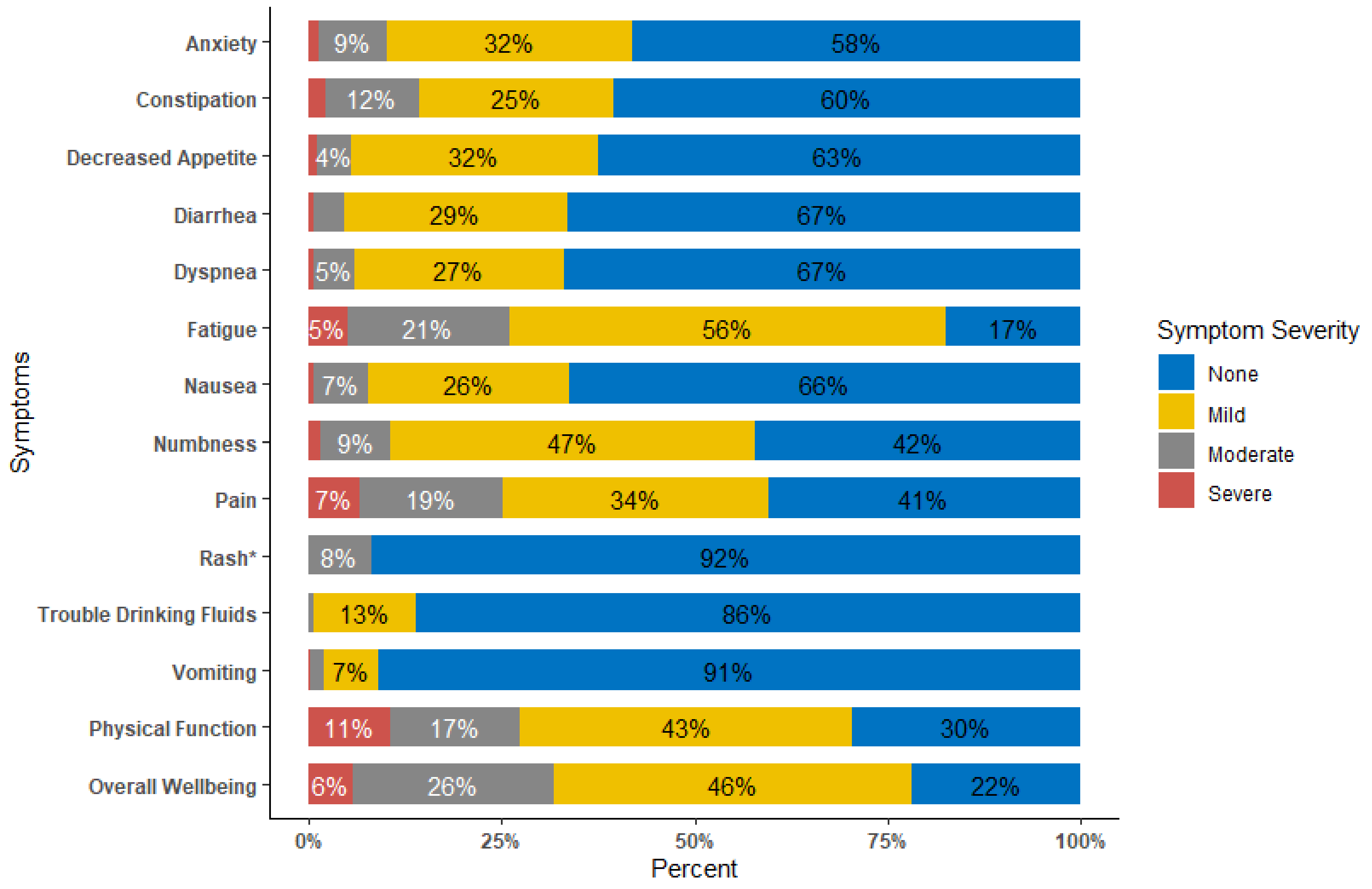

eFigure 6. Distribution of 12 Required Symptoms, Physical Function and Overall Wellbeing by Severity for Patients with GYN Cancer (N=9474 Surveys)
**Rash is binary "Present (Score 2)" or "Absent (Score 0)"*

© 2026 Paudel R et al. *JAMA Network Open.*

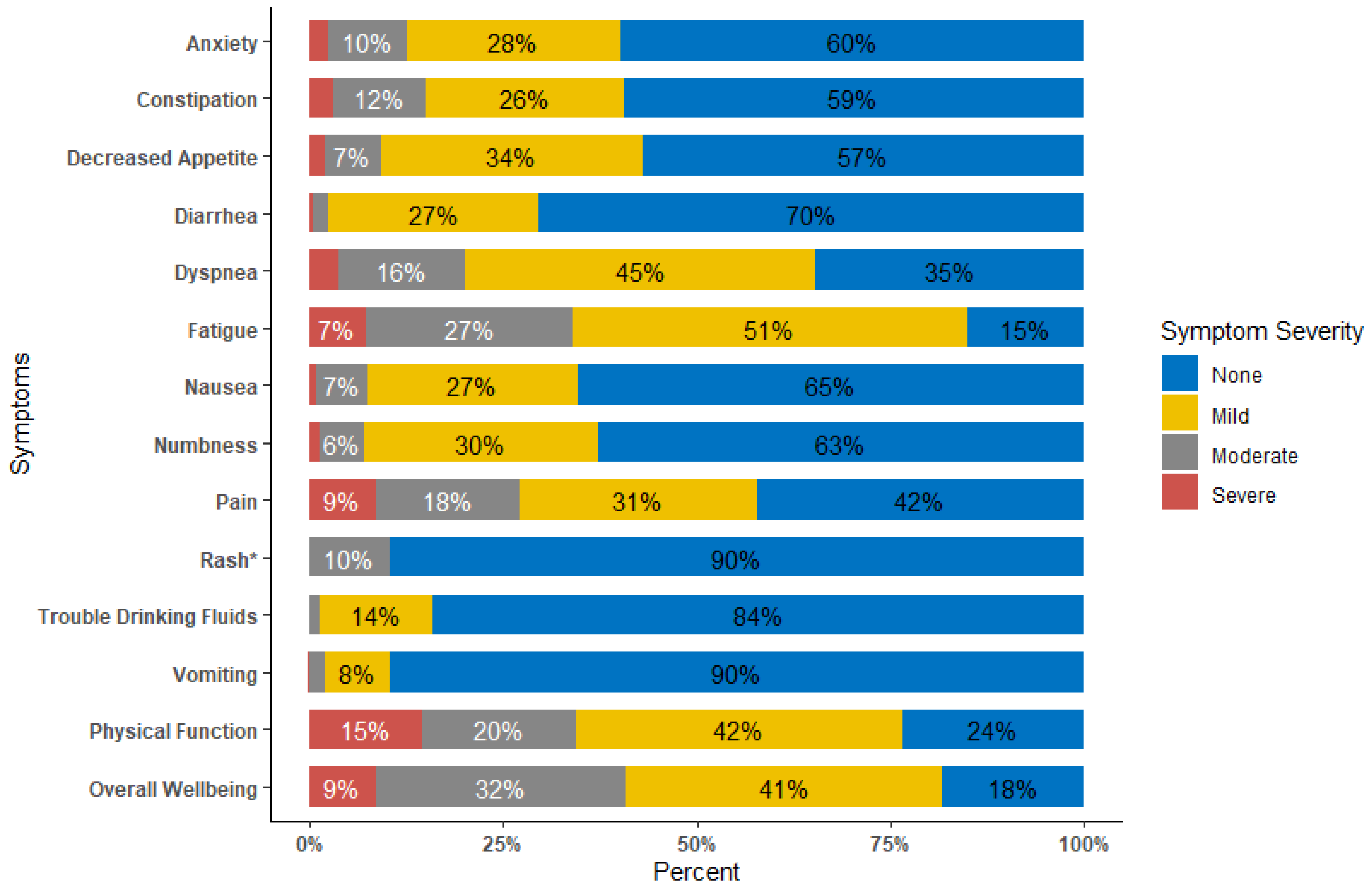

eFigure 7. Distribution of 12 Required Symptoms by Severity for Patients with Thoracic Cancer (N=9967 Surveys)
**Rash is binary "Present (Score 2)" or "Absent (Score 0)"*

© 2026 Paudel R et al. *JAMA Network Open.*
