# Supplementary material for: Longitudinal Patterns of Symptoms in Patients Undergoing Chemotherapy: A Secondary Analysis of a Cluster Randomized Clinical Trial
Source: JAMA Netw Open. 2026 Apr 6;9(4):e264996. doi: 10.1001/jamanetworkopen.2026.4996 (PMC13054620; doi:10.1001/jamanetworkopen.2026.4996)
Supplement: Supplement 3. — Data Sharing Statement [file jamanetwopen-e264996-s003.pdf]

Data Sharing Statement

Paudel. Longitudinal Patterns of Symptoms in Patients Undergoing Chemotherapy. *JAMA Netw Open*. Published April 06, 2026. doi:10.1001/jamanetworkopen.2026.4996

Data
Additional Information: SIMPRO Research Center: Integration and Implementation of PROs for Symptom Management in Oncology Practice. https://cdn.clinicaltrials.gov/large-docs/12/NCT03850912/Prot_000.pdf.
Data available: Yes
Data types: Deidentified participant data, Data dictionary
How to access data: Data will be available via the Harvard Dataverse in early 2026. Request for data can also be sent to Michael_Hassett@dfci.harvard.edu
When available: With publication

Supporting Documents
Document types: Other (please specify)
Additional Information: Study protocol and statistical analysis plan
How to access documents: clinicaltrials.org Data will be available via the Harvard Dataverse in early 2026.
When available: With publication

Additional Information
Who can access the data: Researchers whose proposed use of the data has been approved
Types of analyses: For scientific inquiries and public good.
Mechanisms of data availability: With a signed data access agreement
